# An annotated checklist of Myanmar orchid flora

**DOI:** 10.3897/phytokeys.138.36144

**Published:** 2020-01-10

**Authors:** Ye Lwin Aung, Aye Thin Mu, Mung Htoi Aung, Qiang Liu, Xiaohua Jin

**Affiliations:** 1 State Key Laboratory of Systematic and Evolutionary Botany, Institute of Botany, Chinese Academy of Sciences, Beijing 100093, China Institute of Botany, Chinese Academy of Sciences Beijing China; 2 Southeast Asia Biodiversity Research Institute, Chinese Academy of Sciences, Yezin, Nay Pyi Taw 05282, Myanmar Forest Research Institute, Forest Department Yezin Myanmar; 3 Yunnan Forestry Technological College, Kunming, Yunnan, China Yunnan Forestry Technological College Yunnan China; 4 Forest Research Institute, Forest Department, Yezin, Nay Pyi Taw 05282, Myanmar Southeast Asia Biodiversity Research Institute, Chinese Academy of Sciences Yezin China

**Keywords:** Orchidaceae, Checklist, herbarium specimens, Myanmar

## Abstract

Myanmar is situated in Southeast Asia, where species richness and diversity are very high. Myanmar orchid flora is very rich, but still poorly known because botanical explorations have sharply decreased in Myanmar since 1950. The present study provides a checklist of Myanmar orchid flora which includes 1040 species and 151 genera currently known from Myanmar, based on the herbarium specimens, literature and online databases. The number of species is increased by approximately 200 species more than that given in the checklist of Kress et al. (2003), mainly due to recent discoveries of new species to science and new records for Myanmar. There are 76 endemic species of Orchidaceae in Myanmar. It is estimated that ca. 150–300 species still remain as unidentified and are expected to be discovered in further studies on Myanmar orchid flora.

## Introduction

Southeast Asia is a region of high species richness and endemism, encompassing four major global biodiversity hotspots, namely Indo-Burma hotspot, Sundaland hotspot, Wallacea hotspot and Phillipines hotspot (Myers et al. 2000, Mittermeier et al. 2011). Biodiversity in this region is under various threats to species survival, such as habitat loss and fragmentation, land use change, climate change and deforestation. Under such circumstances, Southeast Asian countries face various challenges for biodiversity conservation and the lack of financial and technical assets (Myers et al. 2000, Sodhi et al. 2004, 2010, Mittermeier et al. 2011).

Myanmar is situated in Southeast Asia and is also part of the Indo-Burma biodiversity hotspot, with high species richness and diversity (Myers et al. 2000, Mittermeier et al. 2011). Due to its broad latitudinal range (tropical to subtropical), topographical (mostly mountainous) and climatic (monsoonal) factors, there are various types of ecosystems in Myanmar, from southern tropical evergreen rainforest ecosystem to northern subtropical montane forest ecosystem through central dry deciduous forests.

On the other hand, as biodiversity research is very limited in Myanmar, many species of fauna and flora still remain unidentified. Biodiversity conservation is urgently needed to secure the sustainability of existing biodiversity resources in Myanmar (Giam et al. 2010, Webb et al. 2010, Forest Department 2015, Jin et al. 2018a). According to the checklist of Myanmar flora (Kress et al. 2003), there are 273 families, 2371 genera and over 11,800 species of vascular plants, including ca. 800 species of Orchidaceae recorded from Myanmar (Kress et al. 2003, Kurzweil and Lwin 2014).

Botanical explorations have sharply decreased in Myanmar since 1950, leading to a large gap of knowledge on flora of Myanmar (Kress et al. 2003). Unlike neighbouring countries with intensive botanic investigation and a modern taxonomic treatment on orchid biodiversity, the orchid flora of Myanmar is very poorly known and lacks a modern taxonomic treatment (see Pearce and Cribb 2002, Ormerod and Sathish Kumar 2003, Ormerod 2005, 2012, Chen et al. 2009, Pedersen et al. 2011, 2014, Kurzweil and Lwin 2014).

At the start of the 21^st^ century, botanical explorations resumed in Myanmar, with the support of international cooperation for biodiversity conservation and research. As a result, there are discoveries of species new to science and new species records for Myanmar orchid flora over recent years (Ormerod 2005, Tanaka et al. 2010, 2015, 2018, Kurzweil et al. 2010, Kurzweil and Lwin 2012a, b, 2014, 2015, Kurzweil 2013, Watthana et al. 2015, Aung et al. 2017, Jin and Kyaw 2017, Liu et al. 2017, Yang et al. 2017, Aung and Jin 2018, Aung et al. 2018, Kurzweil and Ormerod 2018, Liu et al. 2018, Kang et al. 2019, Mu et al. 2019, Ya et al. 2019). In the review on Orchidaceae in the checklist of Kress et al. (2003), there are many species needed to be revised taxonomically due to recent advances in orchid taxonomy and systematics. For example, the genera *Drymoda*Lindley (1838: 8), *Ione*Lindley (1853: 1), *Monomeria*Lindley (1830b: 61), *Sunipia*Lindley (1826a: 14, 21, 25), *Trias*Lindley (1830a: 60) have been merged into *Bulbophyllum*Thouars (1822: 3), leading to nomenclatural changes in *Bulbophyllum* (Pridgeon et al. 2014, Vermeulen et al. 2014).

In addition, there is a sharp increase in the number of species of Orchidaceae due to recent discoveries of species new to science and new species records for Myanmar. For example, one new species of *Bulbophyllum*Thouars (1822: 3), two new species of *Calanthe*Brown (1821: 573), three new species of *Coelogyne*Lindley (1821: 33), two new species of *Dendrobium*Swartz (1799: 2, 6: 82), two new species of *Gastrodia*Brown (1810: 330), one new species of *Pinalia*Lindley (1826b: 14, 21, 23), one new species of *Odontochilus*Blume (1858: 79) and one new species of *Vanda* Jones ex Brown (1820: 6: 506) have been described from Myanmar over recent years (Roberts et al. 2008, Ormerod and Wood 2010, Tanaka et al. 2010, Kurzweil 2013, Aung et al. 2017, 2018, Jin and Kyaw 2017, Liu et al. 2017, 2018, Yang et al. 2017, Aung and Jin 2018, Kurzweil and Ormerod 2018, Zhou et al. 2018a). Recently Kurzweil and Ormerod (2018) also reported 38 new records for Myanmar, also leading to an increase in the number of species of Myanmar orchid flora. Under the main theme of biodiversity conservation and research, the present study investigates the species richness of Myanmar orchid flora, based on the field investigation, herbarium specimens, literature and online databases. The present study will contribute to the floristic studies and biodiversity conservation in Myanmar.

## Material and methods

### Study areas

**Figure 1.**
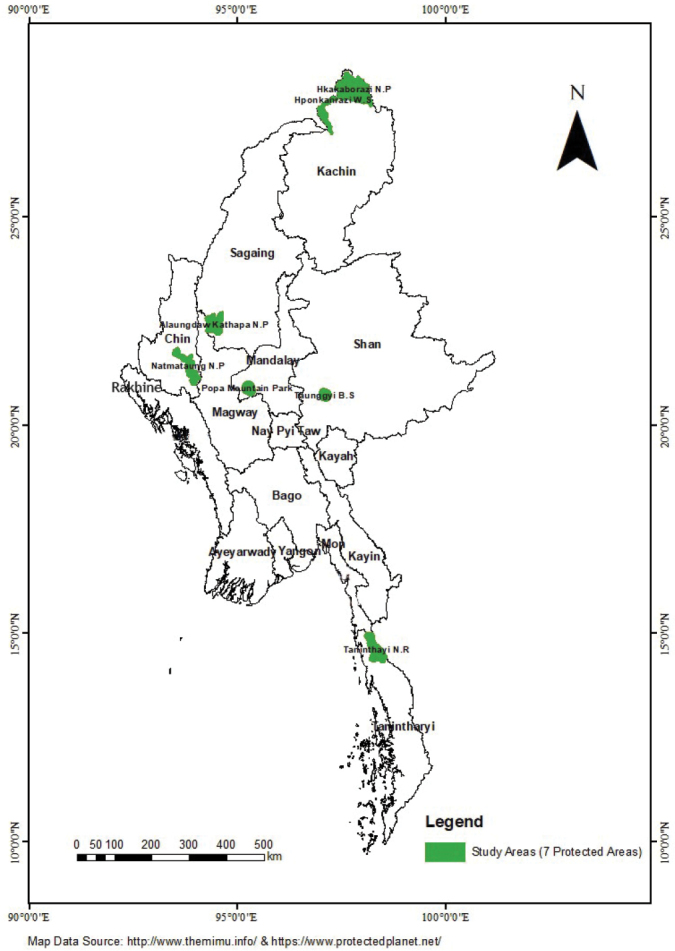
Map of Myanmar, showing the location of study areas for orchid survey.

In order to investigate species richness of Myanmar orchid flora, a programme of fieldwork has been conducted in almost all ecosystems across Myanmar. Seven protected areas across Myanmar, namely Hponkanrazi Wildlife Sanctuary and Hkakaborazi National Park of Kachin State, Popa Mountain Park of Mandalay Region, Nat Ma Taung National Park of Chin State, Alaungdaw Kathapa National Park of Sagaing Region, Taunggyi Bird Sanctuary and its adjacent areas of Shan State and Tanintharyi Nature Reserve of Tanintharyi Region, have been investigated two to five times in each protected area during 2014–2018 (Fig. 1).

### Specimen collections and identification

In total, approximately 1,000 specimens of orchids were collected for vouchers, kept in PE and RAF (Thiers 2019, http://sweetgum.nybg.org/ih/). More than 75% of the total specimens were collected from Hponkanrazi Wildlife Sanctuary and Hkakaborazi National Park, Putao District, Kachin State, Northern Myanmar. Putao is the northernmost district of Myanmar where there are vast areas of primary forest with high species richness and diversity. The remaining proportions were collected from other study areas. In the collections, most species are *Dendrobium* species, *Bulbophyllum* species and *Coelogyne* species. The remaining proportions are species of common genera *Eria*, *Liparis*, and *Oberonia*.

All collected specimens were taxonomically identified based on relevant literature, field notes, photographs taken during fieldwork, herbarium specimens (PE) and online herbarium specimens such as Kew Herbarium Catalogue and Chinese Virtual Herbarium (Seidenfaden 1992, Dressler 1993, Pedersen 1995, Pridgeon et al. 2001, 2005, 2014, Pedersen et al. 2002, 2011, 2014, Kress et al. 2003, Chen et al. 2009, Kurzweil and Lwin 2014, Chase et al. 2015, Chinese Virtual Herbarium 2018, Royal Botanic Gardens Kew 2018). For verification of taxonomic status of all species, literature and online databases, such as WCSP, were reviewed to confirm its respective taxonomic status (Pridgeon et al. 2001, 2005, 2014, Chen et al. 2009, Gardiner 2012, Gardiner et al. 2013, Vermeulen et al. 2014, Jin et al. 2014, Chase et al. 2015, Raskoti et al. 2016, 2017, Ng et al. 2018, WCSP 2019).

### Investigation of herbarium specimens

In total, there were ca. 3,000 herbarium specimens examined, including specimens of our own collections (PE), Kew herbarium specimens (K) and specimen records from online herbaria: AMES, BM, E, GH, K, L, NY, P, US and W (Thiers 2019, http://sweetgum.nybg.org/ih/).

All available datasets of herbarium specimens (ca. 1500 specimens) and specimen photographs were downloaded from online herbaria and examined to enumerate the number of species and to investigate the species occurrences in Myanmar. The following are specimen records downloaded from each online herbarium, AMES (12 records), BM (420 records), E (496 records), GH (3 records), K (324 records), L (39 records), NY (116 records), P (87 records), US (81 records) and W (11 records) (Natural History Museum 2014, Royal Botanic Gardens Kew 2018, The BioPortal of Naturalis Biodiversity Center 2019, Smithsonian National Museum of Natural History 2019, New York Botanical Garden 2019, Royal Botanic Garden Edinburgh 2019, The Index of Botanical Specimens, Harvard University Herbaria and Libraries 2019, The Vascular Plants, Muséum National d’Histoire Naturelle 2019, The Virtual Herbaria, Naturhistorisches Museum Wien 2019). During our field trips in Myanmar, some old herbarium specimens at RAF (ca. 200 specimens) were also examined at herbarium (RAF) of Forest Research Institute in Yezin, Nay Pyi Taw.

As for species occurrences, the herbarium specimens (BM, E, K) provide the information on past record of species occurrences in Myanmar, for example, old collections (past 100 years) by some well-known plant collectors, such as Charles Parish, George Forrest, J. H. Lace, Frank Kingdon-Ward, W. A. Robertson, F. G. Dickason and C. W. D. Kermode during last half of the 19^th^ century and first half of the 20^th^ century. The herbarium specimens (PE) provide information on the current status of species occurrences across Myanmar.

In addition, some literature provides information on specimens from Myanmar that can be cited as specimen-based species occurrences in Myanmar (Kurzweil and Lwin 2015, Tanaka et al. 2015, 2018, Watthana et al. 2015, Aung et al. 2017, 2018, Jin and Kyaw 2017, Liu et al. 2017, 2018, Yang et al. 2017, Aung and Jin 2018, Kurzweil and Ormerod 2018, Zhou et al. 2018a, Ding et al. 2019, Kang et al. 2019, Mu et al. 2019, Ya et al. 2019).

### Verification of species occurrence and taxonomic status

The number of species in the updated checklist is a result of taxonomic attempts, mainly based on our own collections, all available herbarium specimens, checklist of Kress et al. (2003) and all relevant literature. As for taxonomic status, we followed the updated classification of Orchidaceae (Chase et al. 2015) and all relevant papers of orchid taxonomy and systematics (Pridgeon et al. 2001, 2005, 2014, Chen et al. 2009, Gardiner 2012, Gardiner et al. 2013, Jin et al. 2014, Vermeulen et al. 2014, Raskoti et al. 2016, Raskoti et al. 2017, Govaerts 2018, Ng et al. 2018). In addition, all papers of new species descriptions and new records of Myanmar orchid flora were reviewed to identify the species occurrences and distribution in Myanmar (Kress et al. 2003, Roberts et al. 2008, Chen et al. 2009, Tanaka et al. 2010, 2015, 2018, Ormerod 2012, Pedersen et al. 2011, 2014, Kurzweil and Lwin 2012a, b, Kurzweil 2013, Kurzweil and Lwin 2014, Aung et al. 2017, 2018, Jin and Kyaw 2017, Koopowitz et al. 2017, Liu et al. 2017, 2018, Yang et al. 2017, Aung and Jin 2018, Kurzweil and Ormerod 2018, Zhou et al. 2018a, Ding et al. 2019, Kang et al. 2019, Mu et al. 2019, Ya et al. 2019).

Both the number of species and their taxonomic status were also verified with reliable online databases such as the World Checklist of Selected Plant Families (WCSP 2019), International Plant Names Index (IPNI 2012) and the Plant List (The Plant List 2013). Global Biodiversity Information Facility (GBIF 2019) and relevant online herbaria (AMES, BM, E, GH, K, L, NY, P, US and W) were also consulted to check the species occurrences of Orchidaceae in Myanmar.

## Results

**Figure 2.**
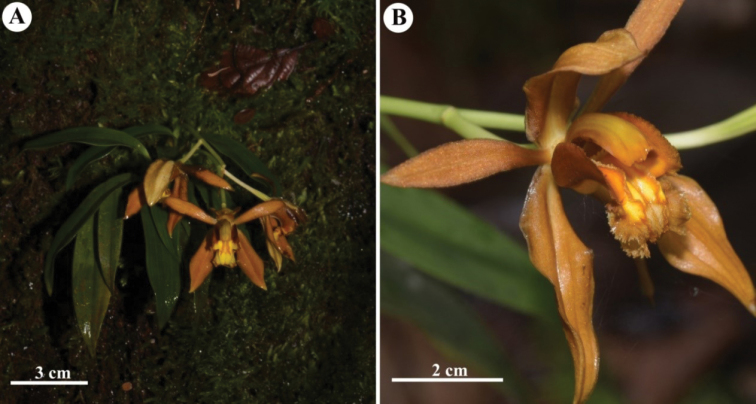
*Coelogyne putaoensis* X.H.Jin, L.A.Ye & Schuit., new species discovered from Myanmar **A** habit of *Coelogyne putaoensis***B** close-up of flower of *Coelogyne putaoensis.* Photos by X.H. Jin.

**Figure 3.**
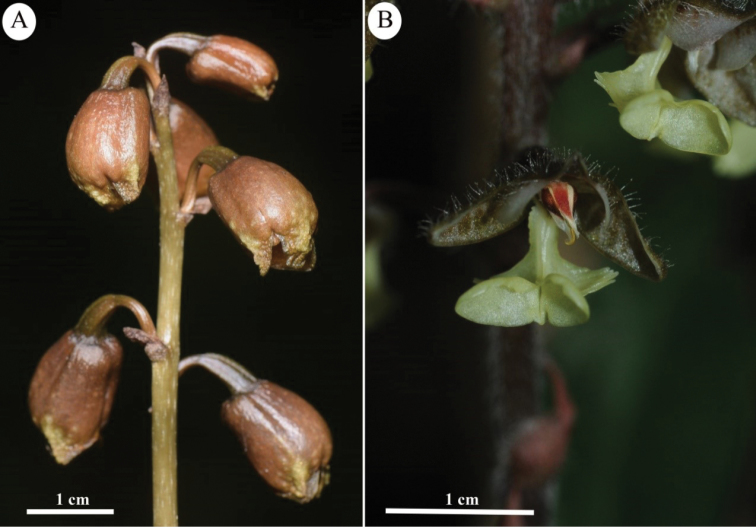
New species discovered from Myanmar **A***Gastrodia kachinensis* X.H.Jin & L.A.Ye. **B***Odontochilus putaoensis* X.H.Jin, L.A.Ye & A.T.Mu. Photos by X.H. Jin.

**Figure 4.**
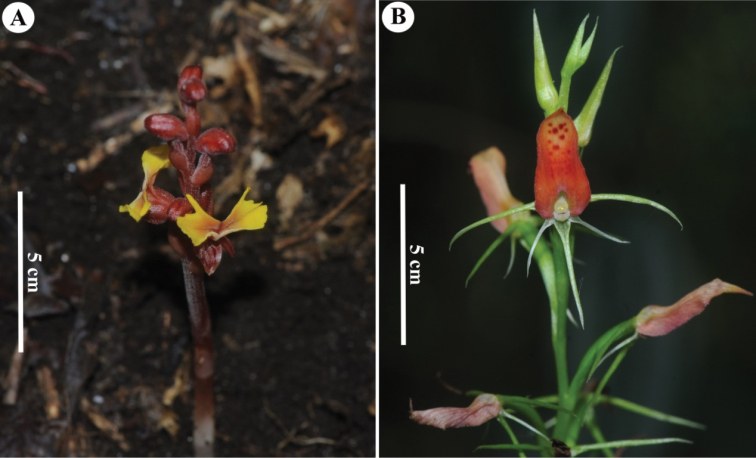
New records discovered from Myanmar **A***Odontochilus poilanei* (Gagnep.) Ormerod **B***Cryptostylis arachnites* (Blume) Hassk. Photos by Ye Lwin Aung.

The present study results in a checklist of Myanmar orchid flora which includes 1040 species in 151 genera, with the number of species increased by ca. 200 species more than that given in the checklist of Kress et al. (2003). The increase in number of species is mainly due to the recent discoveries of species new to science, as well as new records for Myanmar (Figs 2–4). In the last few years, there were 19 new species of Orchidaceae discovered from Myanmar, preliminarily assigned the conservation status based on the IUCN Red List Categories and Criteria (Table 1). In Myanmar, there are 76 endemic species of Orchidaceae which need high conservation attention (IUCN 2012, IUCN Standards and Petitions Subcommittee 2019) (Table 2).

**Table 1.** New species of Orchidaceae discovered from Myanmar.

No.	Species	Conservation status (IUCN)
1	*Biermannia burmanica* Y.H. Tan & Bin Yang	LC
2	*Bulbophyllum putaoensis* Q.Liu	LC
3	*Calanthe kermodei* Ormerod & Kurzweil	LC
4	*Calanthe punctata* Kurzweil	LC
5	*Coelogyne magnifica* Y.H.Tan, S.S.Zhou & B.Yang	EN
6	*Coelogyne putaoensis* X.H.Jin, L.A.Ye & Schuit.	EN
7	*Coelogyne victoria-reginae* Q.Liu & S.S.Zhou	EN
8	*Cylindrolobus glabriflorus* X.H.Jin & J.D.Ya	LC
9	*Dendrobium hkinhumense* Ormerod & Kumar	EN
10	*Dendrobium koyamae* Nb. Tanaka, T. Yuakawa & J. Murata	LC
11	*Dendrobium naungmungense* Q.Liu & X.H.Jin	CR
12	*Gastrodia kachinensis* X.H.Jin & L.A.Ye	EN
13	*Gastrodia putaoensis* X.H.Jin	EN
14	*Liparis popaensis* X.H.Jin, A.T.Mu & L.A.Ye	LC
15	*Odontochilus putaoensis* X.H. Jin, L.A. Ye & A.T. Mu	EN
16	*Paphiopedilum myanmaricum* Koop., Iamwir. & S.Laohap.	EN
17	*Phalaenopsis natmataungensis* (T.Yukawa, Nob.Tanaka & J.Murata) Dalstrom & Ormerod	EN
18	*Pinalia shiuyingiana* Ormerod & Wood	NT
19	*Vanda longitepala* D.L.Roberts, L.M.Gardiner & Mote	EN

**Table 2.** Endemic species of Orchidaceae in Myanmar.

No.	Species endemic to Myanmar	Conservation status (IUCN)
1	*Biermannia burmanica* Y.H. Tan & Bin Yang	LC
2	*Bletilla chartacea* (King & Pantl.) Tang & Wang	EN
3	*Bulbophyllum birmense* Schltr.	DD
4	*Bulbophyllum kachinense* (Seidenf.) J.J.Verm., Schuit. & de Vogel	EN
5	*Bulbophyllum oligoglossum* Rchb. f.	DD
6	*Bulbophyllum putaoensis* Q.Liu	LC
7	*Bulbophyllum sillemianum* Rchb.f.	DD
8	*Calanthe punctata* Kurzweil	LC
9	*Cheirostylis pubescens* Parish & Rchb. f.	DD
10	*Coelogyne magnifica* Y.H.Tan, S.S.Zhou & B.Yang	EN
11	*Coelogyne picta* Schltr.	EN
12	*Coelogyne putaoensis* X.H.Jin, L.A.Ye & Schuit.	EN
13	*Coelogyne victoria-reginae* Q.Liu & S.S.Zhou	EN
14	*Cymbidium parishii* Rchb. f.	CR
15	*Dendrobium aphrodite* Rchb. f.	LC
16	*Dendrobium calothyrsos* Schltr.	EN
17	*Dendrobium hirtulum* Rolfe	EN
18	*Dendrobium hkinhumense* Ormerod & Kumar	EN
19	*Dendrobium koyamae* Nb. Tanaka, T. Yuakawa & J. Murata	LC
20	*Dendrobium laterale* L.O. Williams	EN
21	*Dendrobium leucochlorum* Rchb. f.	EN
22	*Dendrobium luteolum* Bateman	EN
23	*Dendrobium marmoratum* Rchb. f.	DD
24	*Dendrobium naungmungense* Q.Liu & X.H.Jin	CR
25	*Dendrobium pedilochilum* Schltr.	EN
26	*Dendrobium praetermissum* Seidenf.	EN
27	*Dendrobium rhodocentrum* Rchb. f.	EN
28	*Dendrobium sarmentosum* Rolfe	DD
29	*Dilochia subsessilis* (Rolfe) S. Thomas	DD
30	*Eria sicaria* Lindl.	DD
31	*Gastrochilus pechei* Schltr.	DD
32	*Gastrodia kachinensis* X.H.Jin & L.A.Ye	EN
33	*Gastrodia putaoensis* X.H.Jin	EN
34	*Goodyera myanmarica* Ormerod & C.S.Kumar	EN
35	*Habenaria corticicola* W.W. Sm.	EN
36	*Habenaria ditricha* Hook. f.	NT
37	*Habenaria linearis* King & Pantl.	EN
38	*Habenaria massoniana* King & Pantl.	EN
39	*Habenaria mientienensis* Tang & F.T. Wang	NT
40	*Habenaria prazeri* King & Pantl.	EN
41	*Habenaria shweliensis* W.W. Sm. & Banerji	NT
42	*Habenaria spatulifolia* Parish & Rchb. f.	EN
43	*Habenaria triquetra* Rolfe	EN
44	*Habenaria yomensis* Gage	EN
45	*Liparis forrestii* Rolfe	EN
46	*Liparis popaensis* X.H.Jin, A.T.Mu & L.A.Ye	LC
47	*Liparis stenoglossa* Parish & Rchb. f.	DD
48	*Luisia amesiana* Rolfe	NT
49	*Luisia cantharis* Rolfe	NT
50	*Luisia primulina* Parish & Rchb. f.	NT
51	*Micropera secunda* (Rolfe) T. Tang & F.T. Wang	LC
52	*Neottia flabellata* (W.W.Sm.) Szlach.	EN
53	*Neottia unguiculata* (W.W.Sm.) Szlach.	EN
54	*Odontochilus putaoensis* X.H. Jin, L.A. Ye & A.T. Mu	EN
55	*Oreorchis aurantiaca* Pearce & Gibbs	EN
56	*Paphiopedilum myanmaricum* Koop., Iamwir. & S.Laohap.	EN
57	*Papilionanthe sillemiana* (Rchb. f.) Garay	EN
58	*Phalaenopsis natmataungensis* (T.Yukawa, Nob.Tanaka & J.Murata) Dalstrom & Ormerod	EN
59	*Pholidota advena* Parish & Rchb. f.	LC
60	*Pinalia brownei* (Braid) Ormerod	LC
61	*Pinalia dasypus* (Rchb.f.) Kuntze	LC
62	*Pinalia rimannii* (Rchb.f.) Kuntze	LC
63	*Pinalia shanensis* (King & Pantl.) Ormerod	DD
64	*Pinalia shiuyingiana* Ormerod & Wood	NT
65	*Platanthera longibracteata* Lindl.	EN
66	*Renanthera hennisiana* Schltr.	EN
67	*Renanthera pulchella* Rolfe	EN
68	*Rhomboda wardii* Ormerod	LC
69	*Stereochilus laxus* (Rchb. f.) Garay	LC
70	*Tainia hennisiana* (Schltr.) P.F.Hunt	DD
71	*Thunia brymeriana* Rolfe	EN
72	*Thunia candidissima* Rchb. f.	EN
73	*Uncifera verrucosa* Summerh.	DD
74	*Vanda longitepala* D.L.Roberts, L.M.Gardiner & Mote	EN
75	*Vanda vipanii* Rchb. f.	EN
76	*Vandopsis shanica* (Phillimore & W.W. Sm.) Garay	LC

Local distribution information is provided for almost all species, usually at Region/State (Provincial) level distribution ranges (Table 3). Specific distribution locality information is also provided if known for some species. Voucher specimen citations are also provided for almost all species. In cases where herbarium specimens are lacking, the species occurrences are mainly based on the most reliable references of Kress et al. (2003) and Kurzweil and Lwin (2014). By the number of species, most genera consist of one to ten species per genus, while genera *Dendrobium* and *Bulbophyllum* consist of more than 100 species in each (Table 4). The updated checklist is mentioned in the following detail.

**Table 3.** Local distribution of Orchidaceae in Myanmar.

No.	Administrative Provinces	Number of species known
1	Ayeyarwaddy Region	5
2	Bago Region	52
3	Chin State	227
4	Kachin State	394
5	Kayah State	4
6	Kayin State	30
7	Magway Region	29
8	Mandalay Region	219
9	Mon State	187
10	Nay Pyi Taw Union Territory	16
11	Rakhine State	29
12	Sagaing Region	105
13	Shan State	146
14	Tanintharyi Region	307
15	Yangon Region	32

**Table 4.** Number of species in each genus in the updated checklist of Myanmar orchid flora.

No.	Genera	Number of species	No.	Genera	Number of species	No.	Genera	Number of species
1	* Acampe *	5	52	* Diplomeris *	2	103	* Phalaenopsis *	14
2	* Acanthephippium *	1	53	* Diploprora *	2	104	* Pholidota *	11
3	* Acriopsis *	2	54	* Disperis *	1	105	* Pinalia *	33
4	* Aerides *	5	55	* Epipactis *	4	106	* Platanthera *	16
5	* Aeridostachya *	1	56	* Epipogium *	2	107	* Pleione *	10
6	* Agrostophyllum *	6	57	* Eria *	8	108	* Podochilus *	4
7	* Ania *	2	58	* Eriodes *	1	109	* Pogonia *	1
8	* Anoectochilus *	5	59	* Erythrodes *	2	110	* Polystachya *	1
9	* Anthogonium *	1	60	* Erythrorchis *	1	111	* Pomatocalpa *	2
10	* Aphyllorchis *	1	61	* Eulophia *	14	112	* Ponerorchis *	5
11	* Apostasia *	3	62	* Galearis *	3	113	* Porpax *	12
12	* Appendicula *	1	63	* Galeola *	2	114	* Pteroceras *	3
13	* Arachnis *	3	64	* Gastrochilus *	10	115	* Renanthera *	5
14	* Arundina *	1	65	* Gastrodia *	4	116	* Rhomboda *	3
15	* Bambuseria *	1	66	* Geodorum *	5	117	* Rhynchostylis *	2
16	* Biermannia *	1	67	* Goodyera *	11	118	* Risleya *	1
17	* Bletilla *	4	68	* Grammatophyllum *	1	119	* Robiquetia *	3
18	* Brachycorythis *	6	69	* Grosourdya *	1	120	* Saccolabiopsis *	1
19	* Bromheadia *	2	70	* Gymnadenia *	1	121	* Sarcoglyphis *	3
20	* Bryobium *	1	71	* Habenaria *	49	122	* Satyrium *	1
21	* Bulbophyllum *	119	72	* Hemipilia *	3	123	* Schoenorchis *	2
22	* Bulleyia *	1	73	* Herminium *	10	124	* Seidenfadenia *	1
23	* Calanthe *	30	74	* Herpysma *	1	125	* Sirindhornia *	1
24	* Callostylis *	3	75	* Hetaeria *	4	126	* Smitinandia *	2
25	* Cephalanthera *	2	76	* Holcoglossum *	4	127	* Spathoglottis *	4
26	* Cephalantheropsis *	3	77	* Lecanorchis *	3	128	* Spiranthes *	1
27	* Ceratostylis *	4	78	* Liparis *	29	129	* Stereochilus *	3
28	* Cheirostylis *	8	79	* Ludisia *	1	130	* Stereosandra *	1
29	* Chiloschista *	4	80	* Luisia *	14	131	* Stichorkis *	1
30	* Chrysoglossum *	1	81	* Malaxis *	2	132	* Strongyleria *	1
31	* Cleisomeria *	2	82	* Micropera *	5	133	* Taeniophyllum *	1
32	* Cleisostoma *	15	83	* Microsaccus *	1	134	* Tainia *	4
33	* Coelogyne *	45	84	* Mycaranthes *	3	135	* Thecostele *	1
34	* Collabium *	2	85	* Myrmechis *	2	136	* Thelasis *	5
35	* Corymborkis *	1	86	* Neogyna *	1	137	* Thrixspermum *	5
36	* Cremastra *	1	87	* Neottia *	6	138	* Thunia *	5
37	* Crepidium *	8	88	* Nephelaphyllum *	3	139	* Thuniopsis *	1
38	* Cryptochilus *	4	89	* Nervilia *	6	140	* Tipularia *	1
39	* Cryptostylis *	2	90	* Oberonia *	29	141	* Trachoma *	1
40	* Cylindrolobus *	7	91	* Odontochilus *	8	142	* Trichoglottis *	5
41	* Cymbidium *	25	92	* Oreorchis *	5	143	* Trichotosia *	8
42	* Cypripedium *	3	93	* Otochilus *	4	144	* Tropidia *	2
43	* Cyrtosia *	1	94	* Oxystophyllum *	1	145	* Tuberolabium *	1
44	* Dendrobium *	144	95	* Pachystoma *	1	146	* Uncifera *	2
45	* Dendrochilum *	2	96	* Panisea *	6	147	* Vanda *	17
46	* Dendrolirium *	3	97	* Paphiopedilum *	14	148	* Vandopsis *	3
47	* Dickasonia *	1	98	* Papilionanthe *	5	149	* Vanilla *	3
48	* Didymoplexis *	1	99	* Pecteilis *	4	150	* Vrydagzynea *	2
49	* Dienia *	2	100	* Pelatantheria *	3	151	* Zeuxine *	9
50	* Diglyphosa *	1	101	* Peristylus *	10	**Total**		**1040**
51	* Dilochia *	1	102	* Phaius *	6			

## Discussion

Botanical collections are still needed to cover the whole floristic diversity of Myanmar, because botanical explorations have sharply decreased in Myanmar since 1950 (Kress et al. 2003). There is a large gap of knowledge on Myanmar flora. Perhaps there are many species to be discovered in Myanmar. The fundamental data on the number of plant species and its distribution range is still not fully known so that it needs much more research for effective plant conservation in Myanmar. In addition, it needs modern taxonomic treatments for each family or genus so as to update their classification.

The orchid flora of Myanmar is very species-rich but still poorly known until now. The present study resulted in a checklist of Myanmar orchid flora which includes 1040 species and 151 genera. Botanical investigations are, however, still needed to better understand the orchid biodiversity of Myanmar. Compared with neighbouring countries with intensive orchid studies, Myanmar orchid flora have lagged behind being well-documented and well-studied. In this regard, it can be estimated that about 150–300 species still remain unexplored and are expected to be discovered in future studies on Myanmar orchid flora. As for conservation, all orchid species are legally protected by national legislation but most orchids are still under various threats for their survival, such as habitat loss due to deforestion and land use change. Orchids are, however, regarded as special plants in Myanmar society in terms of cultural ornamental purposes. Historically, *Bulbophyllum auricomum* Lindl. has been recognised as a royal flower in Myanmar during the period of Konbaung dynasty (1752–1885). Nowadays, it has been recognised as one of the national flowers of Myanmar, indicating the special attention of Myanmar society on orchids (Hinsley et al. 2018). Thus, it is obvious that the special role of orchids might enhance the orchid biodiversity conservation in Myanmar.

As for plant species richness in Myanmar, there are many new species described from Myanmar over recent years, for example, two new species of Annonaceae, one new species of Aristolochiaceae, one new species of Asteraceae, three new species of Balsaminaceae, one new species of Begoniaceae, two new species of Lamiaceae, one new species of Magnoliaceae, 10 new species of Orchidaceae, one new species of Phyllanthaceae and one new species of Zingiberaceae (Aung et al. 2017, 2018, Jin and Kyaw 2017, Liu et al. 2017, Tan et al. 2017, Tseng et al. 2017, Yang et al. 2017, 2018a, b, Aung and Jin 2018, Ding et al. 2018, Liu et al. 2018, Li and Ren 2018, Ruchisansakun et al. 2018, Tan et al. 2018, Ya et al. 2019, Yao et al. 2018, Zhou et al. 2018a, b, Mu et al. 2019).

Recently there are discoveries of two new plant familial records for Myanmar, namely Petrosaviaceae and Triuridaceae (Jin and Mint 2018, Jin et al. 2018b). It is very obvious that species richness is very high in Myanmar, with evidence of recent discoveries of species new to science and new records. With many more botanical explorations, the knowledge gap on the flora of Myanmar can be filled in the future.

## Checklist

The checklist consists of the following data (Table 5):

**Table 5.** Checklist of Myanmar orchid flora.

No.	Species	Local distribution	Specimen citations
1	*Acampe carinata* (Griff.) Panigr.	Listed as occurrence in Myanmar	Kurzweil and Lwin 2014
2	*Acampe cephalotes* Lindl.	Putao, Kachin; Taunggyi, Shan	Xiaohua Jin et al. PT-5240 (PE!); Ye Lwin Aung PT-7601 (PE!)
3	*Acampe joiceyana* (J.J. Sm.) Seidenf.	Mt. Popa, Mandalay	Khin Myo Htwe 102 (spirit collection-MBK, TNS)
4	*Acampe ochracea* (Lindl.) Hochr.	YePhyu, Tanintharyi; Mt. Victoria, Chin	Ye Lwin Aung PT- 7307, PT-7542, PT-7356 (PE!)
5	*Acampe praemorsa* (Roxb.) Blatter & McCann	Mt. Popa, Mandalay; Bago (Pegu); Tanintharyi	s.coll. s.n. (P!); Khin Myo Htwe 13 (spirit collection-MBK, TNS)
6	*Acanthephippium sylhetense* Lindl.	Chin; Kachin; Magway; Mon; Sagaing; Taunggyi, Shan	Ye Lwin Aung PT-7594 (PE!)
7	*Acriopsis indica* Wight	Tanintharyi Nature Reserve, YePhyu,Tanintharyi; Mt. Victoria, Chin	Ye Lwin Aung PT-7269, PT-7350, PT-7565 (PE!); Hlaing Ko 19 (NY!)
8	*Acriopsis liliifolia* (Koen.) Ormerod	YePhyu, Tanintharyi	Ye Lwin Aung PT-7333 (PE!)
9	*Aerides crassifolia* Parish ex Burb.	Mt. Popa, Mandalay	Khin Myo Htwe 14 (spirit collection-TNS), Tanaka et al. 036165 (MBK)
10	*Aerides falcata* Lindl. & Paxton	Mt. Popa, Mandalay	Lace 6204 (E!); Khin Myo Htwe 33 (spirit collection-TNS)
11	*Aerides multiflora* Roxb.	Mon	Herb. Helfer 5222 (NY!)
12	*Aerides odorata* Lour.	Reported from Myanmar	Kress et al. 2003
13	*Aerides rosea* Lodd. ex Lindl. & Paxton	Putao, Kachin	Xiaohua Jin et al. PT-6998 (PE!)
14	*Aeridostachya crassipes* (Ridl.) Rauschert	Putao, Kachin	Xiaohua Jin et al. PT-2045, PT-2071, PT-5283 (PE!)
15	*Agrostophyllum brevipes* King & Pantl.	Putao, Kachin	Xiaohua Jin et al. PT-5243 (PE!), Ye Lwin Aung PT-7043 (PE!)
16	*Agrostophyllum callosum* Rchb. f.	Chin, Kachin, Magway, Mandalay, Sagaing, Shan, Tanintharyi	Ye Lwin Aung PT-7178 (PE!)
17	*Agrostophyllum glumaceum* Hook.f.	YePhyu, Tanintharyi	Ye Lwin Aung PT-7325, PT-7346 (PE!)
18	*Agrostophyllum planicaule* (Wall. ex Lindl.) Rechb.f.	Mandalay; YePhyu & Dawei (Tavoy), Tanintharyi	Ye Lwin Aung PT-7291, PT-7293 (PE!); R.C.F. Swinhoe 71 (K!); Keenan et al. 5094 (E!)
19	*Agrostophyllum stipulatum* (Griff.) Schltr.	Myeik (Mergui), Tanintharyi	Griffith 5220 (K!)
20	*Agrostophyllum superpositum* Schltr.	Putao, Kachin	Ye Lwin Aung PT-7116 (PE!)
21	*Aniaangustifolia Lindl.*	Dawei (Tavoy), Tanintharyi	Wallich 3740 (E!) & (BM!); Keenan et al. 1596 (E!); Kingdon-Ward 22717 (BM!)
22	*Ania viridifusca* (Hook.) Tang & W.T.Wang ex Summerh.	Chin; Kachin; Kyaikkahmi (Amherst), Mon	Lace 5622 (E!); Keenan et al. 3817 (E!); Kingdon-Ward 21737 (BM!)
23	*Anoectochilus albolineatus* Parish & Rchb. f.	Mt. Victoria, Chin; Moulmein, Mon; Tanintharyi	Parish 325 (K); Kingdon-Ward 22872 (BM!)
24	*Anoectochilus burmannicus* Rolfe	Kadat Reserve, Bago (Pegu)	Roger s.n. (K!)
25	*Anoectochilus chapaensis* Gagnep	Putao, Kachin	Qiang Liu 319 (HTIBC!)
26	*Anoectochilus lylei* Rolfe ex Downie	Kachin; Katha, Sagaing	Kingdon-Ward 20499 (BM!); Lace s.n. (E!)
27	*Anoectochilus roxburghii* Lindl.	Chin; Putao, Kachin	Xiaohua Jin et al. PT-ET 246, PT-ET 1110 (PE!); Kate Armstrong 2331 (NY!); Kingdon-Ward 21519 (BM!)
28	*Anthogonium gracile* Lindl.	Putao, Kachin; Mogok, Mandalay; Shan; Mon; Tanintharyi	Xiaohua Jin et al. PT-ET 454 (PE!); Lace 6016 (E!); MacGregor 818 (E!)
29	*Aphyllorchis montana* Rchb. f.	Chin	Kingdon-Ward 22609 (BM!)
30	*Apostasia nuda* R. Br.	Myeik (Mergui), Tanintharyi	Griffith 100 (K!)
31	*Apostasia odorata* Blume	Putao, Kachin	Xiaohua Jin et al. PT-2544 (PE!)
32	*Apostasia wallichii* R. Br.	Mt. Popa, Mandalay; Putao, Kachin	Xiaohua Jin et al. PT-ET 156 (PE!); Prazer 101 (E!); Keenan et al. 983 (E!); Khin Myo Htwe 030019 (MBK)
33	*Appendicula cornuta* Blume	Putao, Kachin; Tanintharyi	Ye Lwin Aung PT-7171, PT-7180 (PE!)
34	*Arachnis clarkei* J.J.Sm.	Chin; Shan	Kingdon-Ward 22876 & 22718 (BM!); Phillimore s.n. (P!)
35	*Arachnis labrosa* (Lindl. & Paxton) Rchb. f.	Putao, Kachin; Mon; Tanintharyi; Mt. Popa, Mandalay	Xiaohua Jin et al. PT-5342 (PE!); Tanaka et al. 036150 (MBK)
36	*Arachnis siamensis* (Schltr.) Tang & F.T.Wang	Mt. Popa, Mandalay	Khin Myo Htwe 108 (spirit collection-MBK, TNS)
37	*Arundina graminifolia* (D. Don) Hochr.	Chin; Putao, Kachin; Magway; Mandalay; Mon; Sagaing; Shan	Xiaohua Jin et al. PT-6771 (PE!); G. Forrest 24662 (E!); Prazer 102 (E!); MacGregor 823 (E!); Keenan et al. 3928 (E!); Kate Armstrong 2281 (NY!); Kingdon-Ward 22509 (BM!)
38	*Bambuseria bambusifolia* (Lindl.) Schuit., Y.P.Ng & H.A.Pedersen	Sumprabum, Kachin; Southern Shan State	Keenan et al. 3823 (K!) & (E!); W.A. Robertson 84 (K!); Kingdon-Ward 21664 (BM!)
39	***Biermannia burmanica* Y.H. Tan & Bin Yang**	Putao, Kachin	Y.H. Tan, B. Yang, H.B. Ding, M.B. Maw & T.S. Tin M1593 (holotype, HITBC)
40	***Bletilla chartacea* (King & Pantl.) Tang & Wang**	Listed as occurrence in Myanmar	Kurzweil and Lwin 2014
41	*Bletilla foliosa* (King & Pantl.) Tang & Wang	Pyin Oo Lwin (Maymyo), Mandalay	Lace 5883 (E!); English 145 (E!)
42	*Bletilla formosana* (Hayata) Schltr.	Listed as occurrence in Myanmar	Kurzweil and Lwin 2014
43	*Bletilla striata* (Thunb.) Rchb. f.	Kachin; Mon; Taunggyi, Shan	Farrer 994 (E!); Forrest 29728 (E!); Kingdon-ward 16780 (E!); Maung Po Khant 16337 (E!); Dickason 837 (P!)
44	*Brachycorythis acuta* (Rchb.f.) Summerh.	Paungbyin Reserve, Upper Chindwin, Sagaing; Mindat, Chin	Lace 4222 (K!) & (E!); Prazer 89 (E!); Kingdon-Ward 22426 (BM!)
45	*Brachycorythis galeandra* (Rchb. f.) Summerh.	Myikyina, Kachin; Ayeyarwaddy	Mokim 22 (BM!) & 82 (P!) & (US); Belcher 9480 (US)
46	*Brachycorythis helferi* (Rchb. f.) Summerh.	Mt. ZweKaBin, Kayin; Mon	Xiaohua Jin et al. PT-2570 (PE!); Parish s.n. (K!); Helfer 244 (K!); Keenan et al. 890 (E!)
47	*Brachycorythis henryi* (Schltr.) Summerh.	Shan	Maung Po Khant 16312 (E!)
48	*Brachycorythis neglecta* Pedersen	Pyin Oo Lwin (Maymyo), Mandalay; Lashio, Shan	Lace 4827, 5304 & 5837 (E!); Keenan et al. 575 (E!); MacGregor 678 (E!)
49	*Brachycorythis obcordata* (Lindl.) Summerh.	Listed as occurrence in Myanmar	Kurzweil and Lwin 2014
50	*Bromheadia aporoides* Rchb. f.	Mon, Tanintharyi	Kress et al. 2003
51	*Bromheadia finlaysoniana* (Lindl.) Miq.	Listed as occurrence in Myanmar	Kurzweil and Lwin 2014
52	*Bryobium pudicum* (Ridl.) Y.P.Ng & P.J.Cribb	Putao, Kachin	Xiaohua Jin et al. PT-2444 (PE!), Ye Lwin Aung PT-7068, PT-7073, PT-7080, PT-7099, PT-7111 (PE!)
53	*Bulbophyllum affine* Lindl.	Recorded from Myanmar	s.coll.s.n. (E!)
54	*Bulbophyllum alcicorne* Parish & Rchb. f.	Mon, Tanintharyi	Kress et al. 2003
55	*Bulbophyllum amplifolium* (Rolfe) Balakr. & Chowdhury	Listed as occurrence in Myanmar	Kurzweil and Lwin 2014
56	*Bulbophyllum andersonii* (Hook.f.) J.J.Sm.	Putao, Kachin	Ye Lwin Aung PT-7114 (PE!)
57	*Bulbophyllum apodum* Hook.f.	Shan	s.coll. s.n. (K!)
58	*Bulbophyllum auricomum* Lindl.	Taunggoo, Bago; Haka, Chin; Kachin; Mon; Rakhine; Tanintharyi; Yangon	Venning 6 (K!); Wallich 1985 (K!); s.coll. s.n. (K!); Lace s.n. (E!); Reynaud 60 (P!); Griffith s.n. (P!)
59	*Bulbophyllum bifurcatoflorens* (Fukuy.) J.J.Verm., Schuit. & de Vogel	Recorded from Myanmar	Kress et al. 2003
60	***Bulbophyllum birmense* Schltr.**	Reported from Myanmar	Kress et al. 2003
61	*Bulbophyllum blepharistes* Rchb. f.	YePhyu, Tanintharyi	Ye Lwin Aung PT-7245, PT-7341, PT-7252, PT-7260 (PE!)
62	*Bulbophyllum candidum* (Lindl.) Hook.f.	Putao Township, Kachin State	Kurzweil & Saw Lwin KL 2637 (SING, spirit collection)
63	*Bulbophyllum canlaonense* Ames	Putao, Kachin	Ye Lwin Aung PT-7199, PT-7198, PT-7118 (PE!)
64	*Bulbophyllum capillipes* Parish & Rchb. f.	Chin, Mon, Tanintharyi	Kress et al. 2003
65	*Bulbophyllum capnophyton* J.J.Verm., Schuit. & de Vogel	YePhyu, Tanintharyi	Saw Lwin et al. TNRO 162 (SING, SING(spirit), Herbarium of Tanintharyi Nature Reserve Education Centre)
66	*Bulbophyllum careyanum* Spreng.	Paunglaung Creek, Nay Pyi Taw-Shan Yoma; Alaungdaw Kathapa National Park, Sagaing; Mt. Victoria, Chin; Kalaw, Shan	Ye Lwin Aung PT-7383, PT-7384, PT-7452, PT-7453, PT-7577 (PE!); Lois & Soren Egerod B-82 (K!)
67	*Bulbophyllum cariniflorum* Rchb.f.	Listed as occurrence in Myanmar	Kurzweil and Lwin 2014
68	*Bulbophyllum caudatum* Lindl.	Putao, Kachin	Xiaohua Jin et al. PT-2500 (PE!)
69	*Bulbophyllum cauliflorum* Hook. f.	Putao, Kachin; Mon	Ye Lwin Aung PT-7052, PT-7159, PT-7164, PT-7051, PT-7202, PT-7212 (PE!)
70	*Bulbophyllum clandestinum* Lindl.	Myeik (Mergui), Tanintharyi; Andaman Island	S. Kurz s.n. (K!); Griffith 5295 (K!)
71	*Bulbophyllum collettii* King & Pantl.	Listed as occurrence in Myanmar	Kurzweil and Lwin 2014
72	*Bulbophyllum comosum* Collett & Hemsl.	Taunggyi, Shan	H. Collett 100 (K!); s.coll. s.n. (K!)
73	*Bulbophyllum crabro* (C.S.P.Parish & Rchb.f.) J.J.Verm., Schuit. & de Vogel	Putao, Kachin; Kayin; Mon; Tanintharyi	Ye Lwin Aung PT-2264 (PE!); s.coll. s.n. (K!); Kingdon-Ward 10181 (BM!)
74	*Bulbophyllum crassipes* Hook. f.	Kayin; Moulmein, Mon; Rakhine; Alaungdaw Kathapa National Park, Sagaing	Ye Lwin Aung PT-7440, PT-7441 (PE!); A. Meebold 17424 (K!)
75	*Bulbophyllum cupreum* Lindl.	Kachin; Bago; Mon; Tanintharyi	Bull 48 (K!); Lace 6097 (E!)
76	*Bulbophyllum cylindraceum* Wall. ex Lindl.	Putao, Kachin	Xiaohua Jin et al. PT-2389, PT-2547 (PE!), Ye Lwin Aung PT-7107 (PE!)
77	*Bulbophyllum dayanum* Rchb. f.	Mon, Tanintharyi	Kress et al. 2003
78	*Bulbophyllum delitescens* Hance	Putao, Kachin	Qiang Liu 500 (HITBC!)
79	*Bulbophyllum dickasonii* Seidenf.	Putao, Kachin	Xiaohua Jin et al. PT-6143 (PE!); Dickason 8444 (AMES)
80	*Bulbophyllum drymoda* J.J.Verm., Schuit. & de Vogel	Kayin, Tanintharyi	Kress et al. 2003
81	*Bulbophyllum drymoglossum* Maxim.	Putao, Kachin	Xiaohua Jin et al. PT-6891 (PE!)
82	*Bulbophyllum elatum* (Hook.f.) J.J. Sm.	Nogmung Township, Kachin State	Kingdon-Ward 7408 (K)
83	*Bulbophyllum emarginatum* (Finet) J.J.Sm.	Kachin	Kingdon-Ward 73 (BM!) & (NY!)
84	*Bulbophyllum eublepharum* Rchb.f.	Listed as occurrence in Myanmar	Kurzweil and Lwin 2014
85	*Bulbophyllum farreri* (W.W. Sm.) Seidenf.	Htawgaw, Upper Burma	Kingdon-Ward 1560 (E!)
86	*Bulbophyllum forrestii* Seidenf.	Putao & Hpimaw, Kachin	Xiaohua Jin et al. PT-5279 (PE!); Kingdon-Ward 1554 (E!); Forrest 26609 (P!) & (US)
87	*Bulbophyllum gracillimum* (Rolfe) Rolfe	Recorded from Myanmar	s.coll. s.n. (K!)
88	*Bulbophyllum guttulatum* Wall. ex Hook. f.	Reported from Myanmar	Kress et al. 2003
89	*Bulbophyllum haniffii* Carr	Mon, Tanintharyi	Kress et al. 2003
90	*Bulbophyllum helenae* (Kuntze) J.J. Sm.	Mt. Victoria, Chin	Ye Lwin Aung PT-7516, PT-7551 (PE!); Forrest 26609 (E!)
91	*Bulbophyllum hirtum* Lindl.	Putao, Kachin; Chin; Shan; Tanintharyi	Xiaohua Jin et al. PT-5280 (PE!); Keenan et al. 3033 (E!); MacGregor 2524 (E!); Kate Armstrong 2408 (NY!)
92	*Bulbophyllum intricatum* Seidenf.	Putao, Kachin; southwestern part of Shan; Webula, Falam, Chin	Qiang Liu M16-29 (HITBC!); Fujikawa et al. 101848 (MBK); Daun 44 (K!)
93	***Bulbophyllum kachinense* (Seidenf.) J.J.Verm., Schuit. & de Vogel**	Kachin	Kress et al. 2003
94	*Bulbophyllum kanburiense* Seidenf.	Reported from Myanmar	Kress et al. 2003
95	*Bulbophyllum khasyanum* Griff.	Putao, Kachin; Tanintharyi	Qiang Liu 477 (HITBC!)
96	*Bulbophyllum kingii* Hook.f.	Recorded from Myanmar	Kingdon-Ward 13507 (BM!)
97	*Bulbophyllum lasiochilum* Parish & Rchb. f.	Reported from Myanmar	Kress et al. 2003
98	*Bulbophyllum laxiflorum* (Blume) Lindl.	Mon, Tanintharyi	Kress et al. 2003
99	*Bulbophyllum lemniscatum* Parish ex Hook. f.	Mon; Tanintharyi; Taunggoo, Bago	Lhin 4411 (K!); Parish 211 (K!)
100	*Bulbophyllum leopardinum* (Wall.) Lindl.	Mt. Victoria, Chin	Ye Lwin Aung PT-7530 (PE!)
101	*Bulbophyllum lepidum* (Blume) J.J.Sm.	Chin; YePhyu, Tanintharyi	Ye Lwin Aung PT-7358 (PE!)
102	*Bulbophyllum limbatum* Parish & Rchb. f.	Reported from Myanmar	Kress et al. 2003
103	*Bulbophyllum lindleyanum* Griff.	Reported from Myanmar	Kress et al. 2003
104	*Bulbophyllum lineatum* (Teijsm. & Binn.) J.J. Sm.	Bago, Tanintharyi, Yangon	s.coll. 89 (K!)
105	*Bulbophyllum lobbii* Lindl.	Kayin, Rakhine, Tanintharyi	Lace s.n. (E!)
106	*Bulbophyllum longipes* Rchb.f.	Mon, Tanintharyi	Kress et al. 2003
107	*Bulbophyllum lopalanthum* J.J.Verm., Schuit. & de Vogel	Mandalay	Xiaohua Jin et al. PT-5235 (PE!)
108	*Bulbophyllum macphersonii* Rupp	Tanintharyi	Kress et al. 2003
109	*Bulbophyllum macranthum* Hook. f.	Bago, Tanintharyi	Kress et al. 2003
110	*Bulbophyllum microtepalum* Rchb. f.	Mon, Tanintharyi	Kress et al. 2003
111	*Bulbophyllum moniliforme* Parish & Rchb. f.	Mt. Victoria, Chin; Mon; YePhyu, Tanintharyi; Alaungdaw Kathapa National Park, Sagaing	Ye Lwin Aung PT-7407, PT-7337, PT-7480 (PE!); Parish 96 (K!)
112	*Bulbophyllum muscarirubrum* Seidenf.	Listed as occurrence in Myanmar	Kurzweil and Lwin 2014
113	*Bulbophyllum nasutum* Rchb.f.	Mon, Tanintharyi	Parish 263 (K!)
114	*Bulbophyllum nigrescens* Rolfe	Myitkyina, Kachin; PyinOoLwin (Maymyo), Mandalay; Shan	Kermode 17332 (AMES) & (K!); A. Samuel 13537 (K!); Egerod B-17 (US)
115	*Bulbophyllum oblongum* Rchb.f.	Mon, Tanintharyi	Parish 264 (K!); Griffith s.n. (P!)
116	*Bulbophyllum odoratissimum* Lindl.	Myitkyina & Putao, Kachin; Webula, Falam, Chin; Myeik(Mergui), Tanintharyi; Taunggoo, Bago	Xiaohua Jin et al. PT-2043, PT-2073, PT-2385, PT-2457 (PE!); Ye Lwin Aung PT-7042, PT-7117, PT-7161, PT-7162, PT-7165, PT-7048, PT-7194 (PE!); C.Bagg B-13 (K!); Su Kae 10032 (K!); Daun 35 (K!); C.W.D. Kermode 17329 (K!); A.F.G. Kerr 01005 (K!); Inokum s.n. (K!); Mokum s.n. (US)
117	***Bulbophyllum oligoglossum* Rchb. f.**	Reported from Myanmar	Kress et al. 2003
118	*Bulbophyllum orientale* Seidenf.	Recorded from Myanmar	SBGO 3651 (SING, spirit collection)
119	*Bulbophyllum ornatissimum* (Rchb. f.) J.J. Sm.	Reported from Myanmar	Kress et al. 2003
120	*Bulbophyllum parviflorum* Parish & Rchb. f.	Putao, Kachina; Mon; Tanintharyi	Qiang Liu M16-4 (HITBC!)
121	*Bulbophyllum pectinatum* Finet	Mt. Victoria, Chin; Near Black Rock, Myitkyina, Kachin	Ye Lwin Aung PT-7477, PT-7537, PT-7539, PT-7553 (PE!); R.C.F. Swinhoe R 113, 35 (K!); C.W.D. Kermode 17328 (K!); Bull 135 (K!)
122	*Bulbophyllum penicillium* Parish & Rchb. f.	Mon, Tanintharyi	Ye Lwin Aung PT-7330 (PE!); s.coll. s.n. (K!); Parish 303 (K!)
123	*Bulbophyllum pictum* C.S.P.Parish & Rchb.f.	Mon, Tanintharyi	Kress et al. 2003
124	*Bulbophyllum picturatum* (Lodd.) Rchb. f.	Mt. Popa, Mandalay	Khin Myo Htwe 133 (spirit collection-TNS)
125	*Bulbophyllum polliculosum* Seidenf.	Listed as occurrence in Myanmar	Kurzweil and Lwin 2014
126	*Bulbophyllum polyrrhizum* Lindl.	Webula, Kalaymyo, Chin; Shan; Mt. Popa, Mandalay	R.C.F. Swinhoe 91 & 94 (K!); F.G. Dickason 7225 (K!); Egerod B-225 (US); Khin Myo Htwe 98 (spirit collection-MBK)
127	*Bulbophyllum protractum* Hook. f.	Tanintharyi	Kress et al. 2003
128	*Bulbophyllum pseudopicturatum* (Garay) Sieder & Kiehn	Listed as occurrence in Myanmar	Kurzweil and Lwin 2014
129	*Bulbophyllum psittacoglossum* Rchb. f.	Tanintharyi	Kress et al. 2003
130	*Bulbophyllum pteroglossum* Schltr.	Putao, Kachin	Xiaohua Jin et al. PT-ET 1060 (PE!)
131	*Bulbophyllum pumilio* Parish & Rchb. f.	Kalamataung, Mon; Tanintharyi	Parish 220 (K!)
132	*Bulbophyllum purpureofuscum* J.J.Verm., Schuit. & de Vogel	Putao, Kachin	Xiaohua Jin et al. PT-2442 (PE!)
133	***Bulbophyllum putaoensis* Q.Liu**	Wasadam village, Putao County, Kachin State	Q. Liu 330 (holotype, HITBC!)
134	*Bulbophyllum reclusum* Seidenf.	Mt. Popa, Mandalay	Khin Myo Htwe 53 (spirit collection-MBK, TNS), Khin Myo Htwe 25 (spirit collection-MBK, TNS)
135	*Bulbophyllum refractum* (Zoll.) Rchb. f.	Chin, Tanintharyi	Kress et al. 2003
136	*Bulbophyllum reichenbachii* (Kuntze) Schltr.	Kayin, Mon	Parish 200 (K!); s.coll. s.n. (K!)
137	*Bulbophyllum repens* Griff.	Tanintharyi	Kress et al. 2003
138	*Bulbophyllum reptans* Griff.	Putao, Kachin; Mt. Victoria, Chin	Xiaohua Jin et al. PT-2548 (PE!), Ye Lwin Aung PT-7535, PT-7536 (PE!); Dackason 7500 (K!); Forrest 26516 (E!) & (US!); Kate Armstrong 1951 (NY!); Forrest 26516 (NY!); Kingdon-Ward 384 (NY!)
139	*Bulbophyllum retusiusculum* Rchb. f.	Mt. Victoria, Chin; Dawei (Tavoy), Tanintharyi	Keenan et al. 5615 (E!); Cooper 6082 (E!); Forrest 26603 (US)
140	*Bulbophyllum rimannii* (Rchb.f.) J.J.Verm., Schuit. & de Vogel	Shan	Phillimore s.n. (K!)
141	*Bulbophyllum rolfei* (Kuntze) Seidenf.	Nogmung Township, Kachin State	Saw Lwin KSL 1080, 1089 (RAF)
142	*Bulbophyllum roseopictum* J.J.Verm., Schuit. & de Vogel	Putao, Kachin	Kate Armstrong 2063 (NY!)
143	*Bulbophyllum roseum* Ridl.	Mon, Rakhine, Tanintharyi	Kress et al. 2003
144	*Bulbophyllum rufilabrum* Parish	Tanintharyi	Kress et al. 2003
145	*Bulbophyllum rufinum* Rchb. f.	Mandalay; Tanintharyi	R.C.F. Swinhoe K77 (K!)
146	*Bulbophyllum sarcophyllum* (King & Pantl.) J.J. Sm.	Listed as occurrence in Myanmar	Kurzweil and Lwin 2014
147	*Bulbophyllum scabratum* Rchb.f.	Nogmung Township, Chipwi Township, Northeastern Part, Kachin State	Toppin 6265 (K!); Forrest 26603 (K & E!); Saw Lwin KSL 1082 (RAF)
148	*Bulbophyllum scaphiforme* Verm.	Kentung, Shan State	Dickason 9693 (AMES)
149	*Bulbophyllum secundum* Parish & Rchb. f.	Mt. Victoria, Chin; Kachin; Magway; PyinOoLwin (Maymyo), Mandalay; Sagaing; Shan	A. Samuel 13549 (K!); R.C.F. Swinhoe 122 (K!); White 380 (US)
150	*Bulbophyllum serratotruncatum* Rchb. f.	Reported from Myanmar	Kress et al. 2003
151	*Bulbophyllum shanicum* King & Pantl.	Shan	Abdul Huk s.n. (K!)
152	*Bulbophyllum sicyobulbon* Parish & Rchb. f.	Mt. Popa, Mandalay; Mon; Tanintharyi	Ye Lwin Aung PT-7458 (PE!); R.C.F. Swinhoe 95 (K!)
153	***Bulbophyllum sillemianum* Rchb.f.**	Recorded from Myanmar	Perrin s.n. (P!)
154	*Bulbophyllum spathulatum* (Rolfe ex Cooper) Seidenf.	Shan	s.coll. s.n. (K!)
155	*Bulbophyllum stenobulbon* Parish & Rchb. f.	Putao, Kachin; Mon; Tanintharyi	Ye Lwin Aung PT-7197, PT-7186, PT-7192, PT-7218 (PE!)
156	*Bulbophyllum sterile* (Lam.) Suresh	Paunglaung Creek, Nay Pyi Taw-Shan Yoma; Mon	Ye Lwin Aung PT-7375 (PE!)
157	*Bulbophyllum striatum* (Griff.) Rchb.f.	Nogmung Township, Kachin State	Saw Lwin KSL 1063 (RAF)
158	*Bulbophyllum suavissimum* Rolfe	Chin; Kachin; Magway; Mandalay; Kyaikkami(Amherst), Mon; Sagaing; Shan	C.E. Parkinson 5114 (K!); Brassen s.n. (K!); Cooper s.n. (K!); Lace 5606 (E!)
159	*Bulbophyllum sulcatum* (Blume) Lindl.	Putao, Kachin; Mt. Victoria, Chin; YePhyu, Tanintharyi	Ye Lwin Aung PT-7324, PT-7361, PT-7517, PT-7187, PT-7219, PT-7360 (PE!)
160	*Bulbophyllum sunipia* J.J.Verm., Schuit. & de Vogel	Putao, Kachin; Mt. Victoria, Chin; Mon; Taunggyi, Shan; Tanintharyi	Xiaohua Jin et al. PT-2050, PT-2074, PT-2099 (PE!); Ye Lwin Aung PT-7557, PT-7579 (PE!); Su Lay 10014 (K!); Kingdon-Ward 3028 (E!)
161	*Bulbophyllum taeniophyllum* Parish & Rchb. f.	Mon, Shan, Tanintharyi	Kingdon-Ward 20634 (BM!)
162	*Bulbophyllum thaiorum* J.J. Sm.	Listed as occurrence in Myanmar	Kurzweil and Lwin 2014
163	*Bulbophyllum tripudians* Parish & Rchb. f.	Mt. Victoria, Chin	Ye Lwin Aung PT-7518, PT-7519 (PE!)
164	*Bulbophyllum triste* Rchb. f.	Mt. Victoria, Chin; Taunggyi, Shan; Bago; Tanintharyi	Ye Lwin Aung PT-7538 (PE!); Parish 207 (K!); MacKee 6184 (P!)
165	*Bulbophyllum umbellatum* Lindl.	Near Kangfang, Myitkyina & Putao, Kachin; Falam, Chin	Kermode 17288 (K!); Daun 73 (K!); Kingdon-Ward 9348 (BM!)
166	*Bulbophyllum wallichii* Rchb.f.	Kachin; Hkamti, Sagaing	Mokim 5 (E!); Kate Armstrong 3107 (NY!)
167	*Bulbophyllum wendlandianum* (Krzl.) Dammar	Reported from Myanmar	Kress et al. 2003
168	*Bulbophyllum wightii* Rchb. f.	Reported from Myanmar	Kress et al. 2003
169	*Bulbophyllum xylophyllum* Parish & Rchb. f.	Mon, Rakhine, Tanintharyi	Parish 82 (K!)
170	*Bulbophyllum yoksunense* J.J.Sm.	Putao, Kachin	Kingdon-Ward 13304 (BM!)
171	*Bulbophyllum yunnanense* Rolfe	Putao, Kachin	Ye Lwin Aung PT-7035 (PE!)
172	*Bulleyia yunnanensis* Schltr.	Chipwi Township, Kachin State	Kingdon-Ward 1837 & s.n. (E!)
173	*Calanthe alismifolia* Lindl.	Putao Township, Kachin State	Xiaohua Jin et al. PT-2082, PT-2203, PT-2381, PT-2382 (PE!); Kurzweil & Lwin 2693 (SING, spirit)
174	*Calanthe alpina* Hook.f. ex Lindl.	Upper Myanmar, Kachin State; valley of the Dichu	Kingdon-Ward 21170 (AMES); F.K.W. 7126 (K!); Kingdon-Ward 1653 (E); s.coll. S.n. (E!)
175	*Calanthe arcuata* Rolfe	valley of the Dichu; Chipwi Township, Kachin State	Kingdon-Ward 7177 (K!); Kingdon-Ward 1654, 1696, 1721 (E); s.coll. s.n. (E!)
176	*Calanthe baliensis* J.J.Wood & J.B.Comber	Putao, Kachin	Ye Lwin Aung PT-7166 (PE!)
177	*Calanthe biloba* Lindl.	Chin, Kachin, Magway, Mandalay, Sagaing, Shan	Kress et al. 2003
178	*Calanthe brevicornu* Lindl.	Kachin	Kingdon-Ward 3048 (E!)
179	*Calanthe ceciliae* Rchb. f.	Shan	Imschort s.n. (K!)
180	*Calanthe clavata* Lindl.	Recorded from Myanmar	Kingdon-Ward 9088 & 10222 (BM!)
181	*Calanthe densiflora* Lindl.	Putao, Kachin	Ye Lwin Aung PT-7124 (PE!); s.coll. s.n. (E!); Kingdon-Ward 21351 (BM!)
182	*Calanthe griffithii* Lindl.	Upper Myanmar	Forrest 26671 (K!)
183	*Calanthe hancockii* Rolfe	Kangfang-hlawpaw, Myitkyina, Kachin	Lulay 9862 (K!)
184	*Calanthe herbacea* Lindl.	Putao, Kachin	Xiaohua Jin et al. PT-2070 (PE!); Toppin 2736 (E!)
185	*Calanthe kermodei* Ormerod & Kurzweil	Laikam to Fengshuiling road, southeastern part of Myitkyina District, Kachin State	Kermode 17210 (Holotype, AMES!; Isotype, K)
186	*Calanthe labrosa* (Rchb. f.) Rchb. f.	Tanintharyi	Richard Abbott 27951 (NY!)
187	*Calanthe lamellosa* Rolfe	Kachin (Upper Burma)	Forrest 26655 & 26671 (E!) & (US)
188	*Calanthe lyroglossa* Rchb. f.	Chin, Kachin, Magway, Mandalay, Sagaing, Shan	Kress et al. 2003
189	*Calanthe mannii* Hook.f.	Near Htawgaw & Kangfang-Black Rock Road, Myitkyina, Kachin	Kermode 17024 & 17325 (K!); s.coll. s.n. (E!); Kingdon-Ward 20645 & 9456 (BM!)
190	*Calanthe masuca* (D.Don) Lindl.	Kyungdaing-ywa-kyeni-in, MayMyo township, Mandalay	Maung Po Khant 16379 (K!)
191	*Calanthe odora* Griff.	Mandalay, Shan	Kress et al. 2003
192	*Calanthe plantaginea* Lindl.	Listed as occurrence in Myanmar	Kurzweil and Lwin 2014
193	*Calanthe puberula* Lindl.	collected place 6.44 km far from Kang-fang, Myitkyina & Putao, Kachin	Ye Lwin Aung PT-7208 (PE!); Naw Mu Pa 17436 (K!)
194	***Calanthe punctata* Kurzweil**	Yae Kan Taung, Dawei, Tanintharyi	Saw Lwin et al. TNRO 153 (Holotype, SING; Isotype, Myanmar Floriculturist Association)
195	*Calanthe rosea* (Lindl.) Benth.	Mon, Tanintharyi	Kress et al. 2003
196	*Calanthe simplex* Seidenf.	Haka District, Chin State	Daun 101 (K!); Venning 1 (K!)
197	*Calanthe tricarinata* Lindl.	Sunghku laung	Kingdon-Ward 6817 (K!); s.coll. s.n. (E!)
198	*Calanthe trifida* Tang & F.T. Wang	Upper Chindwin, Sagaing	Sulay 9045 (K!)
199	*Calanthe triplicata* (Willem.) Ames	MayMyo Plateau & Mt. Popa, Mandalay; Shan State	Mg Sein 13507 (K!); forest botanist 1554 (K!); Hugh s.n. (K!); Kingdon-Ward 21144 (BM!); Khin Myo Htwe 28 (spirit collection-MBK)
200	*Calanthe trulliformis* King & Pantl.	Listed as occurrence in Myanmar	Kurzweil and Lwin 2014
201	*Calanthe vestita* Lindl.	Mon, Tanintharyi	Kress et al. 2003
202	*Calanthe whiteana* King & Pantl.	Chin; collected place 6.44 km from Kangfang, Myitkyina, Kachin; Magway; Mandalay; Sagaing; Shan	Naw Mu Pa 17417 (K!); Kingdon-ward 1661, 1734 (E!) & (BM!); Farrer 1040 (E!)
203	*Callostylis bambusifolia* (Lindl.) Chen & Wood	Putao, Kachin	Xiaohua Jin et al. PT-5255 (PE!)
204	*Callostylis pulchella* (Lindl.) S.C.Chen & Z.H.Tsi	Kachin, Mandalay, Tanintharyi	Kress et al. 2003
205	*Callostylis rigida* Blume	Myitkyina, Kachin; MayMyo, Mandalay	C.E. Parkinson 2455 (K!); A. Samuel 13528 (K!)
206	*Cephalanthera damasonium* (Mill.) Druce	Kachin (Upper Burma); Kanpetlet, Chin	Forrest 26554 (E!); Cooper 6122 & 6131 (E!)
207	*Cephalanthera pusilla* (Hook. f.) Seidenf.	Bago	s.coll. s.n. (K!)
208	*Cephalantheropsis halconensis* (Ames) S.S.Ying	Putao, Kachin	Ye Lwin Aung PT-7131 (PE!)
209	*Cephalantheropsis longipes* (Hook. f.) Ormerod	Nam Tamai valley, Putao, Kachin	Kingdon-Ward 13443 (E!) & (BM!); Kate Armstrong 2347 (NY!)
210	*Cephalantheropsis obcordata* (Lindl.) Ormerod	Chin, Kachin, Magway, Mandalay, Sagaing, Shan	Griffith 5279 (K!); Kingdon-Ward 21635 & 9141 (BM!)
211	*Ceratostylis himalaica* Hook.f.	Putao, Kachin; Htawgaw, Upper Myanmar	Xiaohua Jin et al. PT-5344 (PE!), Ye Lwin Aung PT-7148 (PE!); Forrest 27015 (K!) & (E!)
212	*Ceratostylis pleurothallis* (Parish & Rchb. f.) Seidenf.	Kayin, Tanintharyi	s.n. (K!)
213	*Ceratostylis radiata* J.J. Sm.	Putao, Kachin	Ye Lwin Aung PT-7076 (PE!); Kurzweil & Lwin 2609 (SING, SING[spirit])
214	*Ceratostylis subulata* Blume	Putao, Kachin	Kate Armstrong 2280 (NY!); Kurzweil & Lwin 2379 (SING [spirit])
215	*Cheirostylis chinensis* Rolfe	Listed as occurrence in Myanmar	Kurzweil and Lwin 2014
216	*Cheirostylis flabellata* (A.Rich.) Wight	Mon	Parish 104 & 123 (K!)
217	*Cheirostylis griffithii* Lindl.	Taunggoo, Bago; Kachin	Parish 236 (K!); Kingdon-Ward 487 (NY!)
218	*Cheirostylis montana* Blume	Tanintharyi	Kress et al. 2003
219	***Cheirostylis pubescens* Parish & Rchb. f.**	Mon, Tanintharyi	Kress et al. 2003
220	*Cheirostylis pusilla* Lindl.	Tanintharyi	Kress et al. 2003
221	*Cheirostylis spathulata* J.J. Sm.	Mindat, Chin; Mt. Popa, Mandalay	Kingdon-Ward 21776 (BM!); Tsujita et al. 036251 (spirit collection-TNS); Khin Myo Htwe 90 (spirit collection-TNS); Tetsuo Ohi-Toma 035000 (MBK)
222	*Cheirostylis yunnanensis* Lindl.	Tamu-Chipwi New Road, Myitkyina, Kachin	Kermode 16649 (K!)
223	*Chiloschista lunifera* (Rchb. f.) J.J. Sm.	Kachin, Mandalay; Mawlamyine (Moulmein), Mon; Tanintharyi	Forrest 12326 (E!); Parish 26237 (W!)
224	*Chiloschista parishii* Seidenf.	Alaungdaw Kathapa National Park, Sagaing	Ye Lwin Aung PT-7398 (PE!)
225	*Chiloschista usneoides* (D. Don) Lindl.	Mt. Popa, Mandalay	Tsujita et al. 036178 (spirit collection-TNS), Khin Myo Htwe 035053 (MBK)
226	*Chrysoglossum assamicum* Hook.f.	Putao, Kachin	Xiaohua Jin et al. PT-ET-190 (PE!)
227	*Chrysoglossum ornatum* Blume	Putao & Nogmung Township, Kachin State	Xiaohua Jin et al. PT-2047, PT-2443 (PE!); Kingdon-Ward 6692 (K)
228	*Cleisomeria lanatum* (Lindl.) Lindl. ex G. Don	Moulmein, Mon; Tanintharyi	s.coll. s.n. (E!)
229	*Cleisomeria pilosulum* (Gagnep.) Seidenf. & Garay	Mt. Popa, Mandalay	Khin Myo Htwe 25, 39 (spirit collection-TNS)
230	*Cleisostoma appendiculatum* (Lindl.) Benth. & Hook. f.	Mon, Tanintharyi	Kress et al. 2003
231	*Cleisostoma arietinum* (Rchb.f.) Garay	Listed as occurrence in Myanmar	Kurzweil and Lwin 2014
232	*Cleisostoma aspersum* (Rchb. f.) Garay	Putao, Kachin; Alaungdaw Kathapa National Park, Sagaing	Ye Lwin Aung PT-7413, PT-7041 (PE!)
233	*Cleisostoma birmanicum* (Schltr.) Garay	Reported from Myanmar	Kress et al. 2003
234	*Cleisostoma duplicilobum* (J.J. Sm.) Garay	Falam, Chin	Daun 74 (K)
235	*Cleisostoma filiforme* (Lindl.) Garay	Falam, Chin; Kachin; PyinOoLwin (Maymyo), Mandalay; Tanintharyi	A. Samuel 13509 (K!); Daun 40 (K!); C.T. Bogg B.G. 7 (K!); s.coll. s.n. (E!); McMillen 259 (US)
236	*Cleisostoma fuerstenbergianum* Kraenzl.	Listed as occurrence in Myanmar	Kurzweil and Lwin 2014
237	*Cleisostoma linearilobatum* (Seidenf. & Smitinand) Garay	Putao Township, Kachin State	Kurzweil & Lwin 2684 (SING [spirit]), Kurzweil & Lwin 2772 (SING [spirit])
238	*Cleisostoma parishii* (Hook. f.) Garay	Putao, Kachin; Mon; Tanintharyi	Xiaohua Jin et al. PT-2052 (PE!)
239	*Cleisostoma racemiferum* (Lindl.) Garay	Mandalay; Kyaikkami(Amherst), Mon; Tanintharyi	R. C. Swinhoe R.123 & 49 (K!)
240	*Cleisostoma rolfeanum* (King & Pantl.) Garay	Taunggyi, Shan	R. C. Swinhoe R.124 (K!)
241	*Cleisostoma rostratum* (Lindl.) Garay	Putao, Kachin	Ye Lwin Aung PT-7127 (PE!); McMillen 7 (US)
242	*Cleisostoma simondii* (Gagnep.) Garay	Reported from Myanmar	Kress et al. 2003
243	*Cleisostoma subulatum* Blume	Reported from Myanmar	Kress et al. 2003
244	*Cleisostoma williamsonii* (Rchb. f.) Garay	Mon, Tanintharyi	Kress et al. 2003
245	*Coelogyne anceps* Hook.f.	Putao, Kachin; Mt. Victoria, Chin	Ye Lwin Aung PT-7033, PT-7153, PT-7575 (PE!)
246	*Coelogyne assamica* Linden & Rchb.f.	Putao, Kachin	Xiaohua Jin et al. PT-5353 (PE!)
247	*Coelogyne barbata* Griff.	valley of the Saunghku & Nwai valley, Putao, Kachin	Kingdon-Ward 7423 (K!); Kingdon-Ward 1941 (E!); Kate Armstrong 1914 (NY!)
248	*Coelogyne brachyptera* Rchb. f.	Mt. Victoria, Chin; Mandalay	Ye Lwin Aung PT-7500, PT-7501, PT-7502 (PE!); R.C.F. Swinhoe 37 (K!)
249	*Coelogyne calcicola* Kerr	Chin; Myitkyina, Kachin	U Aung Din 2902 (K!)
250	*Coelogyne corymbosa* Lindl.	Putao & Myitkyina, Kachin; Paunglaung Creek, Nay Pyi Taw-Shan Yoma; Mt. Victoria & Falam, Chin; Feng-Shiling camp, Upper Myanmar	Xiaohua Jin et al. PT-2115, PT-2204, PT-2256, PT-2266 (PE!), Ye Lwin Aung PT-7390, PT-7494, PT-7509, PT-7545 (PE!); Daun 22 (K!); Forrest 26558 (K!) & (US); Kermode 17357 (K!); Farrer 981 (E!); Kingdon-Ward 1569 & 1630 (E!)
251	*Coelogyne cristata* Lindl.	Shan, Tanintharyi	Kress et al. 2003
252	*Coelogyne ecarinata* C. Schweinf.	Kachin	Kingdon-Ward 434 (NY!) & (AMES!)
253	*Coelogyne fimbriata* Lindl.	Putao, Kachin; Paunglaung Creek, Nay Pyi Taw-Shan Yoma; Mandalay	Xiaohua Jin et al. PT-ET 257, PT-5351 (PE!), Ye Lwin Aung PT-7047, PT-7059, PT-7070, PT-7214, PT-7217, PT-7389 (PE!); R.C.F. Swinhoe 90 (K!)
254	*Coelogyne flaccida* Lindl.	Putao, Kachin; Upper Myanmar; Tanintharyi	Ye Lwin Aung PT-7169, PT-7204 (PE!); F.K.W. 6660 (K!); s.coll. s.n. (K!)
255	*Coelogyne fuscescens* Lindl.	Putao, Kachin; Kayin; Paunglaung Creek, Nay Pyi Taw-Shan Yoma; Mon; YePhyu, Tanintharyi	Ye Lwin Aung PT-7024, PT-7156, PT-7294, PT-7369, PT-7370, PT-7393 (PE!); Generla Tylter B.G. 21 (K!); Parish 195 (K!)
256	*Coelogyne gongshanensis* H.Li ex S.C.Chen	Kachin (Upper Burma)	Forrest 26558 (E!) & (NY!); Farrer 889 & 1561 (E!)
257	*Coelogyne griffithii* Hook.f.	Seinghku-taung, Upper Myanmar	Kingdon-Ward 6730 (K!)
258	*Coelogyne holochila* P.F. Hunt & Summerh.	Mt. Victoria, Chin	Ye Lwin Aung PT-7558 (PE!); Cuffe 1 (K!)
259	*Coelogyne huettneriana* Rchb. f.	Mandalay; Moulmein, Mon	Swinhoe s.n. (K!); Parish 143 (K!)
260	*Coelogyne lentiginosa* Lindl.	Mon; Tanintharyi; Mt. Popa, Mandalay; Southern Shan State	Robertson 137 (K!)
261	*Coelogyne leucantha* W.W. Sm.	Chin; Kachin; Upper Myanmar; Magway; Mandalay; Sagaing; Shan	Forrest 27098 (K!) & (E!); Kingdon-Ward 1669 (E!)
262	*Coelogyne longipes* Hook. f.	Putao, Kachin; Inle lake, Taunggyi, Shan	Xiaohua Jin et al. PT-2110, PT-5097 (PE!)
263	***Coelogyne magnifica* Y.H.Tan, S.S.Zhou & B.Yang**	Hponkanrazi Wildlife Sanctuary, Putao Township, Kachin State	Myanmar Exped. 2046 (holotype, HITBC)
264	*Coelogyne micrantha* Lindl.	Sumprabum & Putao, Kachin; Mt. Victoria, Chin; Mon; Tanintharyi	Xiaohua Jin et al. PT-5273 (PE!), Ye Lwin Aung PT-7014, PT-7026, PT-7210, PT-7572 (PE!); Keenan et al. 3821 (K!) & (E!)
265	*Coelogyne nervosa* A.Rich.	Mt. Victoria, Chin	Ye Lwin Aung PT-7493, PT-7497, PT-7498 (PE!)
266	*Coelogyne nitida* Lindl.	Seinghku taung, Myitkyina, Bhamo, Sumprabum, Putao, Kachin; Haka, Chin; Taunggyi, Shan; Moulmein, Mon; YePhyu, Tanintharyi	Xiaohua Jin et al. PT-5272 (PE!), Ye Lwin Aung PT-7136, PT-7362, PT-7364, PT-7591, PT-7175 (PE!); Maung Wy 4991 (K!); F.K.W. 6799 (K!); C.W.D. Kermode 16683 & 17018 (K!); Venning 50 (K!); J. Keenan et al. 3208 (K!); Parish 150 (K!); Forrest 852 (E!), 26567 (E!) & (P!)
267	*Coelogyne occultata* Hook. f.	Kachin (Upper Myanmar)	Forrest 27060 (K!), (E!) & (NY!); Farrer 1088 (E!); Kingdon-Ward 1767 (E!)
268	*Coelogyne ovalis* Lindl.	Paunglaung Creek, Nay Pyi Taw-Shan Yoma; Kayin; Mon; Sagaing; Bago; Mandalay	Ye Lwin Aung PT-7368, PT-7381 (PE!)
269	*Coelogyne parishii* Hook. f.	Thaton, Mon; Thaungyin, Tanintharyi	Baldwin 13568 (K!); Lace s.n. (E!)
270	***Coelogyne picta* Schltr.**	Chin, Kachin, Mandalay, Sagaing	Kress et al. 2003
271	*Coelogyne prolifera* Lindl.	Putao & Myitkyina, Kachin; Mt. Victoria, Chin; Mandalay; YePhyu, Tanintharyi	Ye Lwin Aung PT-7495, PT-7499, PT-7573, PT-7334, PT-7082 (PE!); Su Lay 10028 (K!); Swinhoe 103 (K!); Cooper 6083 (E!)
272	*Coelogyne pulchella* Rolfe	Mulayit, East Dawna	s.coll. 13527 (K!); s.coll. s.n. (K!)
273	*Coelogyne punctulata* Lindl.	Myitkyina & Putao, Kachin; Mogok, Mandalay	Ye Lwin Aung PT-7130, PT-7029, PT-7036 (PE!); Mg Mya BG 15 (K!); Bogg 13513 (K!); Keenan et al. 3208 & 3079 (E!); Kingdon-Ward 17385 & 18463 (NY!)
274	***Coelogyne putaoensis* X.H.Jin, L.A.Ye & Schuit.**	Hponkanrazi Wildlife Sanctuary, Putao Township, Kachin State	Xiaohua Jin et al. PT-2116 (Holotype, PE!)
275	*Coelogyne rigida* Parish & Rchb. f.	Putao, Kachin; Mon; YePhyu, Tanintharyi	Ye Lwin Aung PT-7106, PT-7357 (PE!); Parish 42 (K!); Kingdon-Ward 20986 & 20650 (BM!)
276	*Coelogyne sanderae* Kranz.	Chin; Putao, Kachin; Magway; Mandalay; Sagaing; Shan	Xiaohua Jin et al. PT-2546 (PE!), Ye Lwin Aung PT-7018 (PE!)
277	*Coelogyne schilleriana* Rchb. f.	Reported from Myanmar	Kress et al. 2003
278	*Coelogyne schultesii* S.K. Jain & S. Das	Mt. Victoria, Kanpetlet, Chin; Myitkyina, Kachin	Ye Lwin Aung PT-7496, PT-7523 (PE!); Dickason 8441 (K!); Kermode 17320 (K!); Kingdon-Ward s.n. (E!); Kingdon-Ward 21816 (BM!)
279	*Coelogyne speciosa* Lindl.	Bago	Kress et al. 2003
280	*Coelogyne stricta* (D. Don) Schltr.	Chin, Kachin, Magway, Mandalay, Sagaing, Shan	s.coll. s.n. (K!)
281	*Coelogyne suaveolens* (Lindl.)	Listed as occurrence in Myanmar	Kurzweil and Lwin 2014
282	*Coelogyne tenasserimensis* Seidenf.	Mandalay	R.C.F. Swinhoe 99 (K!)
283	*Coelogyne testacea* Lindl.	Reported from Myanmar	Kress et al. 2003
284	*Coelogyne tomentosa* Lindl.	Reported from Myanmar	Kress et al. 2003
285	*Coelogyne trinervis* Lindl.	Kyaingtong (Kengtun), Shan; YePhyu, Tanintharyi; Dawna hills-Tenesserim hills, Kyaikkami (Amherst) & Moulmein, Mon	Ye Lwin Aung PT-7359 (PE!); A.F.G. Kerr s.n. (K!); Myron Kimnach 57.268-1 (K!); G.E.R. Cooper 1701 (K!); Bogg B.G. 19 (K!); Keenan et al. 5466 (E!)
286	*Coelogyne triplicatula* Rchb.f.	Dawna Hill, Kayin; Moulmein, Mon	G.E.R. Cooper & C.E. Parkinson 6404 (K!); Parish 160 (K!)
287	*Coelogyne ustulata* Parish & Rchb. f.	Putao, Kachin; Mandalay; Mon; Tanintharyi	Ye Lwin Aung PT-7141 (PE!); Parish 174 (K!); Daun 27 (K!)
288	***Coelogyne victoria-reginae* Q.Liu & S.S.Zhou**	Mt. Victoria, Chin	Ye Lwin Aung PT-7546 (PE!); Qiang Liu M17-18 (holotype, HITBC, isotypes, RAF)
289	*Coelogyne viscosa* Rchb. f.	Mt. Victoria, Chin; Mon; Tanintharyi	R. Unwin 13521 (K!); Parish 252 (K!)
290	*Collabium chapaense* (Gagnep.) Seidenf. & Ormerod	Listed as occurrence in Myanmar	Kurzweil and Lwin 2014
291	*Collabium formosanum* Hayata	Putao, Kachin	Ye Lwin Aung PT-7150 (PE!)
292	*Corymborkis veratrifolia* Blume	Kyaingtong (Kengtun), Shan	Rock 1945 (E!) & (P!) & (US)
293	*Cremastra appendiculata* (D. Don) Makino	Mt. Victoria, Chin; Putao, Kachin	Xiaohua Jin et al. PT-ET 1095 (PE!); K. Fujikawa et al. 089099 (MBK, spirit collection); N. Tanaka et al. 030828 (MBK)
294	*Crepidium acuminatum* (D. Don) Szlach.	Putao, Kachin; Mindat, Chin; Mt. Popa, Mandalay; Moulmein, Mon	Xiaohua Jin et al. PT-2092 (PE!); Parish 147 (K!); Kingdon-Ward 22492 (BM!); Khin Myo Htwe 48 (spirit collection-TNS)
295	*Crepidium biauritum* (Lindl.) Szlach.	Listed as occurrence in Myanmar	Kurzweil and Lwin 2014
296	*Crepidium calophyllum* (Rchb.f.) Szlach.	Putao, Kachin; Bago; Yangon; Maymyo, Mandalay; Moulmein, Mon	Xiaohua Jin et al. PT-6964 (PE!); A. Samuel 13677 (K!); Parish 191 (K!)
297	*Crepidium khasianum* (Hook.f.) Szlach.	Chin	Fujikawa et al. 053114 (MBK), Yasuda 060072 (MBK), Tanaka & Yukawa 081194 (MBK), Ling Shein Man et al. 087417 (MBK), Mu Mu Aung et al. 092458 (MBK), Ling Shein Mang 093057 (MBK), Fujikawa et al. 094196 (MBK)
298	*Crepidium mackinnoniii* (Duthie) Szlach.	Mindat, Chin	Kingdon-Ward 22598 (BM!)
299	*Crepidium polyodon* (Hook.f.) Szlach.	Mon, Tanintharyi	s.coll. 215 (K!)
300	*Crepidium purpureum* (Lindl.) Szlach.	Reported from Myanmar	Kress et al. 2003
301	*Crepidium szemaoense* (Tang & F.T. Wang) Nuammee, Seelanan, Suddee & H.A. Pedersen	Chin	Mu Mu Aung et al. 092359 (MBK)
302	*Cryptochilus luteus* Lindl.	Putao, Kachin	Xiaohua Jin et al. PT-ET 543 & PT-2267 (PE!); s.coll.s.n. (E!); Kingdon-Ward 20984 (BM!)
303	*Cryptochilus roseus* (Lindl.) S.C.Chen & J.J.Wood	Putao, Kachin	Xiaohua Jin et al. PT-5282 (PE!), Ye Lwin Aung PT-7170 (PE!)
304	*Cryptochilus sanguineus* Wall.	Recorded from Myanmar	Kingdon-Ward 12850 (BM!)
305	*Cryptochilus strictus* (Lindl.) Schuit., Y.P.Ng & H.A.Pedersen	Putao, Kachin	Kate Armstrong 1002 (NY!); Kingdon-Ward 9068 & 13524 (BM!)
306	*Cryptostylis arachnites* (Blume) Hassk.	Putao, Kachin	Ye Lwin Aung PT-7144, PT-7196 (PE!)
307	*Cryptostylis carinata* J.J.Sm.	Putao, Kachin	Ye Lwin Aung PT-7071, PT-7100, PT-7113 (PE!)
308	*Cylindrolobus biflorus* (Griff.) Rauschert	Putao, Kachin; YePhyu & Myeik (Mergui), Tanintharyi	Ye Lwin Aung PT-7328, PT-7286, PT-7200 (PE!); Griffith 830 (K!)
309	*Cylindrolobus clavicaulis* (Lindl.) Rauschert	Putao, Kachin; Falam, Chin; Arakan Yoma, Rakhine	Xiaohua Jin et al. PT-5266 (PE!); Ye Lwin Aung PT-7096 (PE!); R.C.F. Swinhoe 78 (K!); Daun 39 (K!)
310	*Cylindrolobus cristatus* (Rolfe) S.C.Chen & J.J.Wood	Putao, Kachin; Mt. Victoria, Chin; Moulmein, Mon	Ye Lwin Aung PT-7157, PT-7503 (PE!); s.coll. s.n. (K!)
311	*Cylindrolobus foetidus* (Aver.) Schuit., Y.P.Ng & H.A.Pedersen	Putao, Kachin	Ye Lwin Aung PT-7094 (PE!)
312	*Cylindrolobus glabriflorus* X.H.Jin & J.D.Ya	Hponkanrazi Wildlife Sanctuary, Putao Township, Kachin State	Xiao-Hua Jin, Ji-Dong Ya 18HT1618 (holotype: KUN!)
313	*Cylindrolobus marginatus* (Rolfe) S.C.Chen & J.J.Wood	Kyaikkahmi (Amherst), Mon	Lace 5644 (E!)
314	*Cylindrolobus truncatus* (Lindl.) Rauschert	Mon, Tanintharyi	Kress et al. 2003
315	*Cymbidium aloifolium* (L.) Sw.	Mt. Victoria, Chin; YePhyu, Tanintharyi	Ye Lwin Aung PT-7315, PT-7354, PT-7531 (PE!); Kingdon-Ward 22138 (BM!)
316	*Cymbidium bicolor* Lindl.	Pantha drainage, Upper Chindwin; Haka & Falam, Chin	Rule s.n. (K!); Glin 5754 (K!); Venning 48 (K!); Daun 66 (K!)
317	*Cymbidium cochleare* Lindl.	Mt. Victoria, Chin	Ye Lwin Aung PT-7532 (PE!)
318	*Cymbidium crassifolium* Wall.	Putao, Kachin; Mt. Popa, Mandalay; Alaungdaw Kathapa National Park, Sagaing; Taunggyi, Shan; YePhyu, Tanintharyi	Ye Lwin Aung PT-7401, PT-7438 (PE!); Ye Lwin Aung PT-7295, PT-7297, PT-7445, PT-7602, PT-7184 (PE!)
319	*Cymbidium cyperifolium* Wall.	Mt. Victoria, Chin; PyinOoLwin (Maymyo), Mandalay	Xiaohua Jin et al. PT-5177 (PE!); R.C.F. Swinhoe 133 (K!)
320	*Cymbidium dayanum* Rchb. f.	Recorded from Myanmar	Rule s.n. (K!)
321	*Cymbidium devonianum* Paxton	Kachin	Kingdon-Ward 20808 (BM!); McMillen 200 (US)
322	*Cymbidium eburneum* Lindl.	Recorded from Myanmar	Kress et al. 2003
323	*Cymbidium elegans* Lindl.	Putao, Kachin; Falam & Haka, Chin	Forrest 27694 (K!) & (E!); Daun 60 (K!); Venning 55 (K!); Kate Armstrong 2087 (NY!); Kingdon-Ward 21617 (BM!)
324	*Cymbidium ensifolium* (L.) Sw.	Mt. Popa, Mandalay	Khin Myo Htwe 118 (spirit collection-TNS), Tanaka et al. 036213 (MBK), Tsujita et al. 036209, 036214 (spirit collection-TNS)
325	*Cymbidium erythraeum* Lindl.	Chin, Kachin, Mandalay, Sagaing	s.coll. s.n. (E!)
326	*Cymbidium faberi* Rolfe	Mt. Victoria, Chin	Cooper 6017 (E!)
327	*Cymbidium finlaysonianum* Lindl.	Listed as occurrence in Myanmar	Kurzweil and Lwin 2014
328	*Cymbidium hookerianum* Rchb. f.	11.27 km from Kangfang, Myitkyina, Kachin	Naw Mu Pa 15503 (K!); Kingdon-Ward 298 (NY!)
329	*Cymbidium iridioides* D. Don	Recorded from Myanmar	s.coll. s.n. (K!)
330	*Cymbidium lancifolium* W.J. Hook.	Putao, Kachin; Mt. Victoria, Chin; Kalaw, Shan; Delu valley	Ye Lwin Aung PT-7056, PT-7524 (PE!); Baldworth 13546 (K!); s.coll. 24 (K!); Kingdon-Ward 8491 (K!); Venning 20 (K!); Kingdon-Ward 1933 (E!)
331	*Cymbidium lowianum* (Rchb. f.) Rchb. f.	Kachin, Yangon	Kingdon-Ward 206 (E!)
332	*Cymbidium macrorhizon* Lindl.	Chin; Shan; Mt. Popa, Mandalay	Prain 12 (K!); Lace s.n. (E!); Kingdon-Ward 17499 (NY!); Kingdon-Ward 20799 & 22157 (BM!); Tanaka et al. 020495 (MBK), Khin Myo Htwe 58 (spirit collection-TNS)
333	*Cymbidium mastersii* Griff. ex Lindl.	Recorded from Myanmar	s.coll. s.n. (E!)
334	***Cymbidium parishii* Rchb. f.**	Kayin, Shan, Tanintharyi	s.coll. s.n. (K!)
335	*Cymbidium sinense* (Jacks.) Willd.	Kachin	Keenan et al. 3301 (E!)
336	*Cymbidium suavissimum* Sander	Recorded from Myanmar	s.coll. s.n. (K!)
337	*Cymbidium tigrinum* Parish ex Hook.	Mulayit, Kayin; Dawna, Kyaikkami (Amherst) & Moulmein, Mon; Tanintharyi	Parish 144 (K!); Bogg B.G. 29 (K!); Shwe Nyar Tha 5 (K!)
338	*Cymbidium tracyanum* Hort.	Chin, Kachin, Shan	Kress et al. 2003
339	*Cymbidium wilsonii* (Rolfe ex Cook) Rolfe	Kalewa, Sagaing	Prazer s.n. (K!); s.coll. s.n. (E!)
340	*Cypripedium guttatum* Sw.	Adung valley, Putao, Kachin	Kingdon-Ward 9677 (BM!)
341	*Cypripedium himalaicum* Rolfe	Adung valley, Putao, Kachin	Kingdon-Ward 9680 (BM!)
342	*Cypripedium lichiangense* S.C. Chen & Cribb	Recorded from Myanmar	Kingdon-Ward 1643 (E!)
343	*Cyrtosia javanica* Blume	Putao, Kachin	Xiaohua Jin et al. PT-6334, PT-6262 (PE!); Myanmar Exped. M4012 (HITBC, RAF)
344	*Dendrobium acerosum* Lindl.	Tanintharyi	Kress et al. 2003
345	*Dendrobium acinaciforme* Roxb.	Putao, Kachin	Xiaohua Jin et al. PT-5348 (PE!)
346	*Dendrobium aduncum* Wall. ex Lindl.	Chin; Kachin; Magway; Maymyo, Mandalay; Alaungdaw Kathapa National Park, Sagaing; YePhyu, Tanintharyi	Ye Lwin Aung PT-7448, PT-7273 (PE!); R.C.F. Swinhoe 132 (K!)
347	*Dendrobium albosanguineum* Lindl. & Paxton	Mon; Rakhine; Tanintharyi; Pyay (Prome), Bago	s.coll. s.n. (K!); Whitin s.n. (US)
348	*Dendrobium aloifolium* (Blume) Rchb. f.	Reported from Myanmar	Kress et al. 2003
349	*Dendrobium amoenum* Wall.	Alaungdaw Kathapa National Park, Sagaing	Ye Lwin Aung PT-7405 (PE!); McMillen 197 (US)
350	*Dendrobium amplum* Lindl.	Putao, Kachin	Ye Lwin Aung PT-7038 (PE!); Kingdon-Ward 21420 (BM!)
351	*Dendrobium anceps* Sw.	Mandalay; Shan; YePhyu, Tanintharyi	Ye Lwin Aung PT-7285, PT-7287, PT-7342 (PE!)
352	*Dendrobium angulatum* Lindl.	YePhyu, Tanintharyi; Eastern Tenasserim	Ye Lwin Aung PT-7327 (PE!); A.F.G. Kerr 01001 (K!) & (P!); Peche 612 (BM!)
353	*Dendrobium angustifolium* (Blume) Lindl.	Putao, Kachin; Tanintharyi	Ye Lwin Aung PT-7132 (PE!)
354	***Dendrobium aphrodite* Rchb. f.**	PyinOoLwin (Maymyo), Mandalay; Mon; Tanintharyi;	Ellison s.n. (K!)
355	*Dendrobium aphyllum* (Roxb.) Fischer	Mt. Popa & Pyin Oo Lwin (Maymyo), Mandalay; foot of YayTheiChin Hill, Upper Chindwin; Falam & Mindat, Chin; Mawlamyine, Mon	Daun 15 (K!); Glin 5821 (K!); Lace s.n. (E!); Kingdon-Ward 21824 (BM!); Lobb 9 (BM!); Khin Myo Htwe 8 (spirit collection-TNS), Khin Myo Htwe 27 (spirit collection-TNS), Khin Myo Htwe 32 (spirit collection-TNS), Khin Myo Htwe 035054 (MBK)
356	*Dendrobium appendiculatum* (Blume) Lindl.	Reported from Myanmar	Kress et al. 2003
357	*Dendrobium attenuatum* Lindl.	Putao, Kachin	Ye Lwin Aung PT-7025 (PE!)
358	*Dendrobium bellatulum* Rolfe	Mt. Victoria, Chin; Taunggyi & Kalaw, Shan	Ye Lwin Aung PT-7527, PT-7528, PT-7584 (PE!); Baldworth 13545 (K!)
359	*Dendrobium bensoniae* Rchb. f.	Mt. Victoria, Chin; Bago; Rakhine; Tanintharyi; Yangon; Mt. Popa & Maymyo, Mandalay	Ye Lwin Aung PT-7555, PT-7563 (PE!); A. Samuel 13575 (K!); s.coll. s.n. (K!); Khin Myo Htwe 35 (spirit collection-TNS), Tanaka et al. 036101, 036103 (spirit collection-MBK, TNS), Tanaka et al. 036193 (spirit collection-MBK, TNS),
360	*Dendrobium bicameratum* Lindl.	Mt. Victoria, Chin; Mandalay; YePhyu, Tanintharyi	Ye Lwin Aung PT-7482, PT-7241 (PE!); R.C.F. Swinhoe 55 & R 126 (K!)
361	*Dendrobium brymerianum* Rchb. f.	Chin; Putao, Kachin; Magway; Mandalay; Sagaing; Shan	Xiaohua Jin et al. PT-6727 (PE!); s.coll. s.n. (K!); Kingdon-Ward 20759 (BM!)
362	*Dendrobium calcariferum* Carr	Putao, Kachin	Ye Lwin Aung PT-7087 (PE!)
363	***Dendrobium calothyrsos* Schltr.**	Reported from Myanmar	Kress et al. 2003
364	*Dendrobium capillipes* Rchb. f.	Haka, Chin; Magway; Mon; Shan; Tanintharyi; Mt. Popa, Mandalay	R.C.F. Swinhoe 43 (K!); Venning 61 (K!); McMillen 203 (US); Khin Myo Htwe 57 (spirit collection-TNS)
365	*Dendrobium cariniferum* Rchb. f.	Chin; Kachin; Mt. Popa, Mandalay; Alaungdaw Kathapa National Park, Sagaing; Taunggyi, Shan	Ye Lwin Aung PT-7404, PT-7595 (PE!); Kingdon-Ward s.n. (E!); Khin Myo Htwe 95 (spirit collection-TNS), Tanaka et al. 036151 (MBK)
366	*Dendrobium chiengmaiense* Schuit. & Peter B.Adams	Reported from Myanmar	Kress et al. 2003
367	*Dendrobium chrysanthum* Lindl.	Chin; Putao, Kachin; Mt. Popa, Mandalay; Taunggyi, Shan	Ye Lwin Aung PT-7105 (PE!); F.G. Dickason 8279 (K!)
368	*Dendrobium chryseum* Rolfe	Mt. Popa & PyinOoLwin (MayMyo), Mandalay; Alaungdaw Kathapa National Park, Sagaing; YePhyu, Tanintharyi	Ye Lwin Aung PT-7423, PT-7274 (PE!); Cooper 6084 (E!); Khin Myo Htwe 5 (spirit collection-TNS); Maung Sin 13538 (K!)
369	*Dendrobium chrysocrepis* Parish & Rchb. f. ex Hook. f.	Mon; YePhyu, Tanintharyi	Ye Lwin Aung PT-7310 (PE!)
370	*Dendrobium chrysotoxum* Lindl.	Mt. Victoria, Chin; Kachin; Magway; Mt. Popa, Mandalay; Alaungdaw Kathapa National Park, Sagaing; Shan; Yangon	Ye Lwin Aung PT-7248, PT-7450, PT-7486, PT-7492, PT-7556, PT-7576, PT-7254 (PE!); Kingdon-Ward 21807 (BM!); McMillen 247 (US); Harrison s.n. (US); Khin Myo Htwe 2 (spirit collection-TNS), Tanaka et al. 036164 (MBK)
371	*Dendrobium comatum* (Blume) Lindl.	Reported from Myanmar	Kress et al. 2003
372	*Dendrobium compactum* Rolfe ex W. Hackett	PyinOoLwin (MayMyo), Mandalay	Lace 6282 (K!) & (E!); Swinhoe 70 (K!); Samuel 13515 (K!);
373	*Dendrobium conspicuum* Bakh.f.	Tanintharyi	Kress et al. 2003
374	*Dendrobium convexum* (Blume) Lindl.	Reported from Myanmar	Kress et al. 2003
375	*Dendrobium crepidatum* Lindl.	Chin, Kachin, Mandalay, Sagaing, Shan, Mt. Popa of Madalay Region	Ye Lwin Aung PT-7366, PT-7377, PT-7380, PT-7382, PT-7387, PT-7463, PT-7488, PT-7580 (PE!); s.coll. s.n. (NY!); Tanaka et al. 036184, 036186 (MBK), Khin Myo Htwe 19 (spirit collection-TNS)
376	*Dendrobium cruentum* Lindl.	Tanintharyi	Kress et al. 2003
377	*Dendrobium crumenatum* Sw.	Tanintharyi	Griffith 5149 (P!); s.coll. s.n. (NY!)
378	*Dendrobium crystallinum* Rchb. f.	Mt. Popa, Mandalay; Mon; Rakhine; Shan; Tanintharyi	Abdul Khalil s.n. (BM!) & (L!) & (P!); Khin Myo Htwe 3 (spirit collection-TNS), Khin Myo Htwe 21 (spirit collection-TNS)
379	*Dendrobium cumulatum* Lindl.	Chin; Kachin; Mandalay; Sagaing; Dawei (Tavoy), Tanintharyi	Keenan et al. 1620 (E!)
380	*Dendrobium curviflorum* Rolfe	Putao, Kachin; PyinOoLwin (MayMyo), Mandalay	Qiang Liu 440 (HITBC!); Swinhoe B.G. 2 (K!); Mg Sein 13503 (K!)
381	*Dendrobium cuspidatum* Lindl.	Dawei (Tavoy), Tanintharyi	Swinhoe 64 (K!); Keenan et al. 5309 (E!); Lobb 406 (BM!)
382	*Dendrobium dantaniense* Guillaumin	Mt. Popa, Mandalay	Khin Myo Htwe 53, 54 (spirit collection-TNS), Tanaka et al. 036154 (MBK)
383	*Dendrobium delacourii* Guill.	Mt. Popa & PyinOoLwin (Maymyo), Mandalay; Thayet, Magway; Mon; Rakhine; Tanintharyi	Lace 6262 & 6357 (E!); Buchanan-Hamilton s.n. (BM!); Khin Myo Htwe 36 (spirit collection-TNS)
384	*Dendrobium denneanum* Kerr	Recorded from Myanmar	F.G. Dickason 8365 (K!)
385	*Dendrobium densiflorum* Lindl. ex Wall.	Paunglaung Creek, Nay Pyi Taw-Shan Yoma; Alaungdaw Kathapa National Park, Sagaing; Taunggyi, Shan; YePhyu, Tanintharyi; Bamaw, Kachin; Pyin Oo Lwin, Mandalay	Ye Lwin Aung PT-7367, PT-7451, PT-7590, PT-7272 (PE!); Parkinson 8722 (RAF!); U Tha Hla 1939 (RAF!)
386	*Dendrobium denudans* D. Don	Mt. Victoria, Chin; Kachin; Alaungdaw Kathapa National Park, Sagaing	Ye Lwin Aung PT-7402, PT-7409, PT-7476 (PE!); Kingdon-Ward 108 (NY!)
387	*Dendrobium devonianum* Paxton	Chin; Kachin; Kayin; Kadaik, Thaton, Mon; Homalin stream, Upper Chindwin & Katha District, Sagaing; Tanintharyi	Glin 6575 (RAF!); Sou Po Chin 5842 (RAF!); AR 492 (RAF!)
388	*Dendrobium dickasonii* L.O. Williams	Mt. Victoria, Haka & Falam, Chin; Taunggyi, Shan; YePhyu, Tanintharyi; Madalay	Ye Lwin Aung PT-7474, PT-7585, PT-7261 (PE!); Daun 21 (K!); R.C.F. Swinhoe 36 (K!); Venning 60 (K!); Hildebrand D (K!); Dickason 8756 & 7779 (AMES!); Kingdon-Ward 22222 (BM!); Kingdon-Ward 22244 (BM!)
389	*Dendrobium dixanthum* Rchb. f.	Bhamo, Kachin; Mon; Tanintharyi	Bogg 13534 (K!); Swinhoe R 128 (K!)
390	*Dendrobium draconis* Rchb. f.	Mt. Victoria, Chin; Mt. Popa, Mandalay; Alaungdaw Kathapa National Park, Sagaing	Ye Lwin Aung PT-7394, PT-7561 (PE!), Khin Myo Htwe 34 (spirit collection-TNS)
391	*Dendrobium ellipsophyllum* Tang & F.T. Wang	Mandalay	R.C.F. Swinhoe 60 (K!); s.coll. s.n. (K!)
392	*Dendrobium eriiflorum* Griff.	Putao, Kachin; Chin; Paunglaung Creek, Nay Pyi Taw-Shan Yoma; Mandalay; Myaukhlaing reserve, Insein, Yangon	Xiaohua Jin et al. PT-5288 (PE!), Ye Lwin Aung PT-7378 (PE!); Swinhoe 84 (K!); Po Khant 696 (RAF!); Mokim s.n. (BM!)
393	*Dendrobium falconeri* Hook.	Chin; Kachin; Sagaing; Taunggyi, Shan	Ye Lwin Aung PT-7587 (PE!); s.coll.s.n. (E!); Kingdon-Ward 20951 (BM!)
394	*Dendrobium farmeri* Paxton	Mon, Tanintharyi	Kress et al. 2003
395	*Dendrobium fimbriatum* Hook.	Chin; valley of the Seinghku, Putao, Kachin; Kayah; Mon; Rakhine; Sagaing; YePhyu, Tanintharyi; Yangon	Xiaohua Jin et al. PT-2019 (PE!); Ye Lwin Aung PT-7317, PT-7318 (PE!); Kingdon-Ward 7256 (K!)
396	*Dendrobium findlayanum* Parish & Rchb. f.	Shan, Yangon	Parish 192 (K!); Hallowell s.n. (US)
397	*Dendrobium finetianum* Schltr.	Putao, Kachin	Ye Lwin Aung PT-7119, PT-7182, PT-7201 (PE!)
398	*Dendrobium formosum* Roxb. ex Lindl.	Chin, Kachin, Bago, Mon, Rakhine, Sagaing, Tanintharyi, Yangon	Wallace 136 (BM!); s.coll. s.n. (K!); Mokim S 634 (L!)
399	*Dendrobium fugax* Rchb.f.	Listed as occurrence in Myanmar	Kurzweil and Lwin 2014
400	*Dendrobium fuscescens* Griff.	valley of the Seinghku, Putao, Kachin	Kingdon-Ward 7623 (K!); Kate Armstrong 2130 (NY!)
401	*Dendrobium fytchianum* Bateman	Mon; Nothern Bago (Pegu); Rakhine; Tanintharyi; Nyanglepin reserve, Taikkyi range, Insein, Yangon	C.W.D. Kermode 16760 (K!); Ba Pe 340 (RAF!)
402	*Dendrobium gibsonii* Paxt.	Putao, Kachin; Shan; Bago; Mandalay; Myeik (Mergui), Tanintharyi	Xiaohua Jin et al. PT-2085 (PE!); Griffith 1040 (K!); Kaulback 123, 124 & 387 (BM!); Kingdon-Ward 21205 (BM!)
403	*Dendrobium grande* Hook.f.	Tanintharyi	Helfer 5065 (K!)
404	*Dendrobium gratiosissimum* Rchb. f.	Mon, Tanintharyi	R.C.F. Swinhoe R 107 (K!)
405	*Dendrobium griffithianum* Lindl.	YePhyu, Tanintharyi	Ye Lwin Aung PT-7249, PT-7321 (PE!); Griffith 1038 (P!)
406	*Dendrobium hancockii* Rolfe	Recorded from Myanmar	Forrest 29786 (BM!)
407	*Dendrobium harveyanum* Rchb. f.	Mt. Victoria, Chin; Mt. Popa, Mandalay	Ye Lwin Aung PT-7456, PT-7457, PT-7483, PT-7487 (PE!)
408	*Dendrobium hendersonii* A.D. Hawkes & A.H. Heller	Reported from Myanmar	Kress et al. 2003
409	*Dendrobium hercoglossum* Rchb.f.	Listed as occurrence in Myanmar	Kurzweil and Lwin 2014
410	*Dendrobium heterocarpum* Wall.	Chin; Kachin; Magway; Mt. Popa, Mandalay; Sagaing; Shan	Mokim s.n. (P!); Khin Myo Htwe 75 (spirit collection-MBK)
411	***Dendrobium hirtulum* Rolfe**	Haka, Chin	Daun 26 (K!); s.coll. s.n. (K!)
412	***Dendrobium hkinhumense* Ormerod & Kumar**	Kachin	Kingdon-Ward 21198 (BM!)
413	*Dendrobium hookerianum* Lindl.	Mindat, Chin; Putao, Kachin	Xiaohua Jin et al. PT-ET 487, PT-ET 669 (PE!); Farrer 1089 (RAF!); Kingdon-Ward 1776 (E!); Kingdon-Ward 21777 & 21333 (BM!)
414	*Dendrobium hymenanthum* Rchb. f.	Reported from Myanmar	Kress et al. 2003
415	*Dendrobium incurvum* Lindl.	Putao, Kachin; Shan; YePhyu, Tanintharyi	Ye Lwin Aung PT-7280, PT-7021, PT-7030, PT-7054, PT-7060, PT-7163, PT-7277 (PE!); Parish 176 (K!)
416	*Dendrobium indivisum* (Blume) Miq.	Kayin, Mon, Rakhine, Sagaing, Tanintharyi	Kerr 1002 (P!)
417	*Dendrobium indragiriense* Schltr.	YePhyu, Tanintharyi	Ye Lwin Aung PT-7331 (PE!)
418	*Dendrobium infundibulum* Lindl.	Mt. Victoria, Falam & Haka, Chin; Kayah; Kayin; Magway; Mandalay; Kyaikkami(Amherst), Mon; Rakhine; Shan	Xiaohua Jin et al. PT-5169 (PE!); J.H. Lace 6294 (K!); Daun 9 (K!); Po Khant 2408 (RAF!); Unwin 3002 & 3077 (E!); Kingdon-Ward 21999 (BM!)
419	*Dendrobium jenkinsii* Wall. ex Lindl.	Alaungdaw Kathapa National Park, Sagaing; PyinOoLwin (Maymyo), Mandalay; YePhyu, Tanintharyi	Ye Lwin Aung PT-7396, PT-7403, PT-7298 (PE!); A. Samuel 13535 (K!)
420	*Dendrobium kentrophyllum* Hook. f.	Tanintharyi	Kress et al. 2003
421	***Dendrobium koyamae* Nb. Tanaka, T. Yuakawa & J. Murata**	Mt. Victoria, Chin	Nobuyuki Tanaka et al. 20040042 (holotype-MBK, spirit collection)
422	*Dendrobium lamellatum* (Blume) Lindl.	Mon, Tanintharyi	Kress et al. 2003
423	*Dendrobium lasioglossum* Rchb. f.	Reported from Myanmar	Kress et al. 2003
424	***Dendrobium laterale* L.O. Williams**	Haka, Chin	Venning 7 (K!); Dickason 7359 & 7378 (AMES!)
425	***Dendrobium leucochlorum* Rchb. f.**	Mon, Tanintharyi	Kress et al. 2003
426	*Dendrobium lindleyi* Steud.	Kachin; Kayin; Mandalay; Mon; Rakhine; Sagaing; Shan; Tanintharyi; Yangon; Tamon reserve, Taunggoo, Bago	Ba Pe 9391 (RAF!); Griffith 1014 (P!)
427	*Dendrobium lituiflorum* Lindl.	Reported from Myanmar	Kress et al. 2003
428	*Dendrobium longicornu* Lindl.	Putao, Kachin; Mt. Victoria & Haka, Chin; Paunglaung Creek, Nay Pyi Taw-Shan Yoma; YePhyu, Tanintharyi	Xiaohua Jin et al. PT-ET 801, PT-2102, PT-2402, PT-5196 (PE!), Ye Lwin Aung PT-7031, PT-7195, PT-7339, PT-7363, PT-7376, PT-7562 (PE!); Venning 2 (K!); Kingdon-Ward 5505 & 3531 (E!); Kate Armstrong 1911 (NY!)
429	***Dendrobium luteolum* Bateman**	Chin, Mon, Tanintharyi	s.coll. s.n. (K!)
430	*Dendrobium macraei* Lindl.	Putao, Kachin	Xiaohua Jin et al. PT-2499 (PE!)
431	*Dendrobium macrostachyum* Lindl.	Kyaukpadaung Township, Mandalay Region	Saw Lwin MPO 050 (SING, SING [spirit])
432	*Dendrobium mannii* Ridl.	Mandalay	R.C.F. Swinhoe 3 (K!)
433	***Dendrobium marmoratum* Rchb. f.**	Reported from Myanmar	Kress et al. 2003
434	*Dendrobium minutiflorum* Kraenzl.	Putao, Kachin	Xiaohua Jin et al. PT-5287, PT-5352 (PE!)
435	*Dendrobium moniliforme* (L.) Sw.	Mt. Victoria, Chin; Lukpyi-Kyanyingu Rd, Myitkyina & Putao, Kachin	Ye Lwin Aung PT-7544 (PE!); Xiaohua Jin et al. PT-6142 (PE!); C.W.D. Kermode 17123 (K!); Farrer 853 (E!); Kingdon-Ward 22139 (BM!)
436	*Dendrobium monticola* P.F.Hunt & Summerh.	Putao, Kachin	Qiang Liu 307 (HITBC!)
437	*Dendrobium moschatum* (Buch.-Ham.) Sw.	Ayeyarwaddy; Bago; Chin; Kayin; Mt. Popa, Mandalay; Magway; Mon; Rakhine; Shan; Tanintharyi; Yangon	Buchanan-Hamilton s.n. (BM!); Tanaka et al. 036153 (MBK)
438	*Dendrobium nathanielis* Rchb. f.	Mon, Shan, Tanintharyi	Maung Hla 3672 (K!); Kurz 360 (BM!)
439	***Dendrobium naungmungense* Q.Liu & X.H.Jin**	Naungmung Township, Putao County, Kachin State	Xiaohua Jin et al. PT-ET 133, PT-ET 1232 (PE!); Qiang Liu 430 (Holotype, HITBC)
440	*Dendrobium nobile* Lindl.	Chin; Putao, Kachin; Sagaing	Ye Lwin Aung PT-7091 (PE!); Forrest 26605 (E!) & (US); Kingdon-Ward 1553 (E!); McMillen 204 (US)
441	*Dendrobium numaldeorii* C. Deori, Hynn. & Phukan	Chipwi Township, Kachin State	Kingdon-Ward 108 (AMES, NY)
442	*Dendrobium ochreatum* Lindl.	Mt. Victoria & Falam, Chin; Mt. Popa, Mandalay	Ye Lwin Aung PT-7455, PT-7473, PT-7481, PT-7484, PT-7491 (PE!), Tanaka et al. 036156 (MBK); Daun 23 (K!)
443	*Dendrobium pachyglossum* Parish & Rchb. f.	Mon, Tanintharyi, Mandalay	R.C.F. Swinhoe 63 (K!)
444	*Dendrobium pachyphyllum* (Kuntze) Bakh. f.	Ayeyarwaddy, Bago, Kayin, Mon, Rakhine, Tanintharyi, Yangon	Kress et al. 2003
445	*Dendrobium palpebrae* Lindl.	Moulmein, Mon	s.coll. s.n. (K!)
446	*Dendrobium panduriferum* Hook. f.	Bago, Yangon	Kress et al. 2003
447	*Dendrobium parcum* Rchb. f.	Chin; Kachin; Magway; Mt. Popa, Mandalay; Paunglaung Creek, Nay Pyi Taw-Shan Yoma; Sagaing; Shan; Tanintharyi	Ye Lwin Aung PT-7388 (PE!); Swinhoe 92 (K!); Forrest 12608 (E!); Khin Myo Htwe 18 (spirit collection-TNS), Khin Myo Htwe 035056 (MBK);
448	*Dendrobium parishii* Rchb. f.	Mt. Victoria, Chin; Kachin; Mt. Popa, Mandalay; Alaungdaw Kathapa National Park, Sagaing; Shan; Mon; Tanintharyi	Ye Lwin Aung PT-7447, PT-7554, PT-7540 (PE!), Khin Myo Htwe 7 (spirit collection-TNS), Khin Myo Htwe 30 (spirit collection-TNS)
449	***Dendrobium pedilochilum* Schltr.**	Reported from Myanmar	Kress et al. 2003
450	*Dendrobium peguanum* Lindl.	Bago, Mon, Tanintharyi	Beddome 8183 (BM!)
451	*Dendrobium pendulum* Roxb.	Chin, Mon, Rakhine, Shan	Kress et al. 2003
452	*Dendrobium plicatile* Lindl.	Mt. Victoria, Chin; Mandalay; Alaungdaw Kathapa National Park, Sagaing; Tanintharyi	Ye Lwin Aung PT-7449, PT-7564 (PE!)
453	*Dendrobium polyanthum* Lindl.	Mt. Popa, Mandalay; Taunggyi, Shan	Ye Lwin Aung PT-7586 (PE!); Kingdon-Ward 22062 (BM!); McMillen 198 (US); Khin Myo Htwe 49 (spirit collection-TNS), Khin Myo Htwe 94 (spirit collection-MBK, TNS)
454	*Dendrobium porphyrochilum* Lindl.	Kalaw & Taunggyi, Shan	Xiaohua Jin et al. PT-5100 (PE!); Ye Lwin Aung PT-7588, PT-7589 (PE!); Kingdon-Ward 20985 & 22670 (BM!)
455	*Dendrobium praecinctum* Rchb.f.	Ho Pone Township, Shan State	Xiaohua Jin et al. PT-6738 (PE!); Saw Lwin SL 49 (herbarium of Myanmar Floriculturist Association)
456	***Dendrobium praetermissum* Seidenf.**	Listed as occurrence in Myanmar	Kurzweil and Lwin 2014
457	*Dendrobium pulchellum* Roxb. ex Lindl.	Bago; Mt. Popa, Mandalay; Rakhine; Tanintharyi; Yangon	Mokim s.n. (BM!); Khin Myo Htwe 11 (spirit collection-TNS), Tanaka 036159 (MBK)
458	*Dendrobium pycnostachyum* Lindl.	Tanintharyi	Beddome 8124 (BM!)
459	*Dendrobium revolutum* Lindl.	Recorded from Myanmar	Peche s.n. (BM!)
460	***Dendrobium rhodocentrum* Rchb. f.**	Reported from Myanmar	Kress et al. 2003
461	*Dendrobium rotundatum* (Lindl.) Hook.f.	Chin; Myitkyina, Kachin	Kermode 17164 (K!); Kingdon-Ward 20686 (BM!)
462	*Dendrobium ruckeri* Lindl.	Nogmung, Kachin	Kingdon-Ward 6645 (K!);
463	*Dendrobium salaccense* (Blume) Lindl.	Reported from Myanmar	Kress et al. 2003
464	***Dendrobium sarmentosum* Rolfe**	Recorded from Myanmar	s.coll. s.n. (K!)
465	*Dendrobium scabrilingue* Lindl.	Bago; Thaton & Moulmein, Mon; Tanintharyi; Ywathit Salween valley, Karmai	Glin 6440 (K!); s.coll. s.n. (K!); Parish 134 (K!)
466	*Dendrobium secundum* (Blume) Lindl.	Rakhine; Thaungyin, Tanintharyi; Yangon; Mandalay	Bogg 13536 (K!); R.C.F. Swinhoe 26 (K!)
467	*Dendrobium senile* Parish ex Rchb. f.	Mon, Sagaing	s.coll. s.n. (K!)
468	*Dendrobium signatum* Rchb. f.	Southern Shan	W. A. Robertson 272 (K!); Hildebrand A, B & C (K!); s.coll. s.n. (K!)
469	*Dendrobium spatella* Rchb. f.	Chin; Kachin; Magway; Mandalay; Alaungdaw Kathapa National Park, Sagaing; Shan; Thaton, Mon	Ye Lwin Aung PT-7417, PT-7418 (PE!); L. T. Bogg 13553 (K!)
470	*Dendrobium strongylanthum* Rchb. f.	Recorded from Myanmar	Kingdon-Ward 22843 (BM!)
471	*Dendrobium stuposum* Lindl.	Moulmein, Mon; Tanintharyi	Parish 358 (K!)
472	*Dendrobium sulcatum* Lindl.	Mt. Victoria, Chin	Ye Lwin Aung PT-7485 (PE!)
473	*Dendrobium sutepense* Rolfe ex Downie	Kalaw, Shan	Xiaohua Jin et al. PT-5098 (PE!)
474	*Dendrobium terminale* Parish & Rchb. f.	Mandalay; Alaungdaw Kathapa National Park, Sagaing; Ye Phyu & Dawei (Tavoy), Tanintharyi	Ye Lwin Aung PT-7323, PT-7421 (PE!); Keenan et al. 5467 (K!); Kerr 1006 (P!)
475	*Dendrobium thyrsiflorum* Rchb. f.	Mt. Popa, Mandalay	Xiaohua Jin et al. PT-5347 (PE!), Khin Myo Htwe 103 (spirit collection-MBK, TNS)
476	*Dendrobium tortile* Lindl.	YePhyu, Tanintharyi	Ye Lwin Aung PT-7319 (PE!); Helfer 5050 (K!)
477	*Dendrobium transparens* Wall.	Bago; Mt. Victoria, Chin; Putao, Kachin; PyinOoLwin (Maymyo), Mandalay; Taunggyi, Shan	Xiaohua Jin et al. PT-2458 (PE!), Ye Lwin Aung PT-7596 (PE!); A. Samuel 13567 (K!); Daun 8 (K!); Cooper 6090 (E!)
478	*Dendrobium treutleri* (Hook.f.) Schuit. & Adams	Putao, Kachin	Kate Armstrong 1918 (NY!); Kingdon-Ward 21446 (BM!)
479	*Dendrobium trigonopus* Rchb. f.	Recorded from Myanmar	s.coll. s.n. (K!)
480	*Dendrobium unicum* Seidenf.	Listed as occurrence in Myanmar	Kurzweil and Lwin 2014
481	*Dendrobium venustum* Teijsm. & Binn.	Mt. Popa & PyinOoLwin (Maymyo), Mandalay	A. Samuel 13562 (K!); J.H. Lace 6262, 6357 (K!); Tanaka et al. 036163 (MBK)
482	*Dendrobium virgineum* Rchb. f.	Reported from Myanmar	Kress et al. 2003
483	*Dendrobium wardianum* Warner	Chin, Rakhine	Kingdon-Ward 21778 (BM!)
484	*Dendrobium wattii* (Hook. f.) Rchb. f.	Mt. Victoria, Chin	Ye Lwin Aung PT-7475 (PE!); Kimnach B - 16 (US)
485	*Dendrobium wightii* A.D. Hawkes & A.H. Heller	Sagaing	Kress et al. 2003
486	*Dendrobium williamsonii* Day & Rchb. f.	Black Rock, Myitkyina & Putao, Kachin; Pyin Oo Lwin, Mandalay; Upper Myanmar	Ye Lwin Aung PT-7135, PT-7028, PT-7133, PT-7137, PT-7155 (PE!); Forrest 26608 (K!), (E!) & (US); Kermode 17330 (K!); Kingdon-Ward 6659 (K!); Tha Hla 1942 (RAF!); Kingdon-Ward 20771 (BM!)
487	*Dendrobium xanthophlebium* Lindl.	Mon, Tanintharyi	Kress et al. 2003
488	*Dendrochilum longifolium* Rchb. f.	Kachin, “Birma: Bhamo”	Rchb.f. 13763 W
489	*Dendrochilum pallidiflavens* Blume	Tanintharyi	Kress et al. 2003
490	*Dendrolirium laniceps* (Rchb.f.) Schuit., Y.P.Ng & H.A.Pedersen	Kyaikkahmi (Amherst), Mon	Lace 5594 (E!)
491	*Dendrolirium lasiopetalum* (Willd.) S.C.Chen & J.J.Wood	Putao, Kachin; Mt. Popa, Mandalay; Alaungdaw Kathapa National Park, Sagaing; YePhyu, Tanintharyi	Xiaohua Jin et al. PT-5277 (PE!), Ye Lwin Aung PT-7098, PT-7229, PT-7230, PT-7092, PT-7185, PT-7234, PT-7243, PT-7332, PT-7442, PT-7454 (PE!)
492	*Dendrolirium tomentosum* (J.Koenig) S.C.Chen & J.J.Wood	Bago, Tanintharyi	Abdul Huk s.n. (BM!)
493	*Dickasonia vernicosa* L.O.Williams	Kyaukkut Chaung, Falam & Haka, Chin	Venning 4 & 5 (K!); F.G. Dickason 7377 & 7524 (K!), 8576 (K!) & (AMES!) & (US); Daun 13 (K!)
494	*Didymoplexis pallens* Griff.	Mandalay; YePhyu, Tanintharyi	Ye Lwin Aung PT-7338 (PE!)
495	*Dienia cylindrostachya* Lindl.	Chin	Kingdon-Ward 9758a (BM!); Matsumoto 053614 (MBK), Mu Mu Aung et al. 092798 (MBK), Fujikawa et al. 094517 (MBK), Fujikawa et al. 094853 (MBK), Thet Yu Nwe TY 61 (RAF)
496	*Dienia ophrydis* (Koenig) Seidenf.	Mt. Popa, Mandalay; NyaungShwe, Shan	Xiaohua Jin et al. PT-5080 (PE!); Kingdon-Ward 12791 (BM!); Tanaka et al. 036192 (TNS); Khin Myo Htwe 43 (spirit collection-TNS)
497	*Diglyphosa latifolia* Blume	Putao, Kachin	Xiaohua Jin et al. PT-5236 (PE!)
498	***Dilochia subsessilis* (Rolfe) S. Thomas**	Recorded from Myanmar	s.coll. s.n. (K!)
499	*Diplomeris hirsuta* (Lindl.) Lindl.	Putao, Kachin	Kingdon-Ward 13089 (BM!)
500	*Diplomeris pulchella* D. Don	Kachin; Mandalay; Upper Chidwin, Sagaing	Boxall s.n. (K!); Toppin 4226 (E!); Rogers 1024 (E!); Kingdon-Ward 1926 (E!); Keenan et al. 3984 (E!); Kingdon-Ward 9030 (BM!)
501	*Diploprora championii* Hook. f.	Putao, Kachin; Tanintharyi	Xiaohua Jin et al. PT-ET 1323, PT-ET 1379 (PE!)
502	*Diploprora truncata* Rolfe ex Downie	Chin	Srisanga et al. 97741 (US)
503	*Disperis neilgherrensis* Wight	Ywangan, Taunggyi, Shan	Kang et al. 2019 [photographic record from Shan State]
504	*Epipactis flava* Seidenf.	Pyin Oo Lwin (Maymyo), Mandalay; Muse, Shan	H. Collett s.n. (K!); F.G. Dickason 6122 (Holotype: AMES)
505	*Epipactis helleborine* (L.) Crantz	Mandalay	Kress et al. 2003
506	*Epipactis mairei* Schltr.	Upper Myanmar	Kingdon-Ward 1667 (E!)
507	*Epipactis royleana* Lindl.	Upper Myanmar	Farrer 1078 (E!)
508	*Epipogium aphyllum* Sw.	Listed as occurrence in Myanmar	Kurzweil and Lwin 2014
509	*Epipogium roseum* (D. Don) Lindl.	Mt. Popa, Mandalay; Mt. Victoria, Kanpetlet, Chin; Putao, Kachin	Xiaohua Jin et al. PT-6483 & PT-6938 (PE!); Cooper 5951A (E!); Kuroiwa et al. 051335 MBK
510	*Eria albiflora* Rolfe	Putao, Kachin	Ye Lwin Aung PT-7055, PT-7090, PT-7101 (PE!)
511	*Eria clausa* King & Pantl.	Putao, Kachin	Ye Lwin Aung PT-7084 (PE!)
512	*Eria coronaria* (Lindl.) Rchb. f.	Mt. Victoria & Haka, Chin	Ye Lwin Aung PT-7525 (PE!); Venning 54 (K!)
513	*Eria javanica* (Sw.) Blume	Mon; YePhyu, Tanintharyi	Ye Lwin Aung PT-7262A, PT-7314 (PE!)
514	*Eria laniceps* Rchb. f.	Tanintharyi	Kress et al. 2003
515	*Eria scabrilinguis* Lindl.	YePhyu, Tanintharyi	Ye Lwin Aung PT-7353 (PE!)
516	***Eria sicaria* Lindl.**	Tanintharyi	Kress et al. 2003
517	*Eria vittata* Lindl.	Listed as occurrence in Myanmar	Kurzweil and Lwin 2014
518	*Eriodes barbata* (Lindl.) Rolfe	Reported from Myanmar	Kress et al. 2003
519	*Erythrodes blumei* (Lindl.) Schltr.	Putao, Kachin	Xiaohua Jin et al. PT-5354 (PE!)
520	*Erythrodes hirsuta* (Griff.) Ormerod	Chin	Kress et al. 2003
521	*Erythrorchis altissima* (Blume) Blume	YePhyu, Tanintharyi	Ye Lwin Aung PT-7268 (PE!)
522	*Eulophia andamanensis* Rchb. f.	Mon; Tanintharyi; Mt. Popa, Mandalay	Khin Myo Htwe 9 (spirit collection-MBK, TNS), Khin Myo Htwe 035063 (MBK)
523	*Eulophia bicallosa* (D. Don) P.F. Hunt & Summerh.	Chin, Kachin, Magway, Mandalay, Sagaing, Shan	Kress et al. 2003
524	*Eulophia bracteosa* Lindl.	Tanintharyi	Kress et al. 2003
525	*Eulophia dabia* (D. Don) Hochr.	Listed as occurrence in Myanmar	Kurzweil and Lwin 2014
526	*Eulophia flava* (Lindl.) Hook. f.	PyinOoLwin (Maymyo), Mandalay; Lwelin loihonmein Taung & Taunggyi Reserve, Shan	Maung Po Khant 16310 & 16319 (E!); Lace s.n. (E!)
527	*Eulophia graminea* Lindl.	Kachin; Tanintharyi; Mt. Popa, Mandalay	Khin Myo Htwe 44, 100 (spirit collection-TNS)
528	*Eulophia herbacea* Lindl.	Chin; Kachin; Magway; PyinOoLwin (Maymyo), Mandalay; Sagaing; Lwelin lymonmein Taung & Taunggyi Reserve, Shan	Khan 24 (K!); English 160 (E!); Lace s.n. (E!); Maung Po Khant 16307 & 16315 (E!); Abdul Kholil s.n. (BM!)
529	*Eulophia macrobulbon* (Parish & Rchb. f.) Hook. f.	Kachin; Mt. Victoria & Mindat, Chin; Mon	Cooper 6077 (E!); Kingdon-Ward 22186 (BM!)
530	*Eulophia nuda* Lindl.	Bago; Mt. Popa & PyinOoLwin (Maymyo), Mandalay; Mon; Lwelin Lymon Mein Taung & Taunggyi Reserve, Shan; Tanintharyi	Maung Po Khant 16305 & 16317 (E!); Lace s.n. (E!); Prazer s.n. (E!); Kingdon-Ward s.n. (E!); Kingdon-Ward 22228 & 22207 (BM!); Khin Myo Htwe 107 (spirit collection-MBK)
531	*Eulophia promensis* Lindl.	Hmaing-Yee, Shan; Bago	Maung Po Khant 16352 (E!)
532	*Eulophia pulchra* (Thouars) Lindl.	Reported from Myanmar	Kress et al. 2003
533	*Eulophia sooi* Chun & Tang ex Chen	Listed as occurrence in Myanmar	Kurzweil and Lwin 2014
534	*Eulophia zollingeri* (Rchb. f.) J.J.Sm.	Mt. Popa, Mandalay	Tanaka et al. 036244 (MBK)
535	*Eulophia siamensis* Rolfe ex Downie	Mt. Victoria, Chin	X.H. Jin 24553 (PE!)
536	*Galearis spathulata* (Lindl.) Hunt	Listed as occurrence in Myanmar	Kurzweil and Lwin 2014
537	*Galearis tschiliensis* (Schltr.) P.J.Cribb, S.W.Gale & R.M.Bateman	Valley of Dichu, Kachin	F.K.W. 7197 (K!)
538	*Galearis wardii* (W.W. Sm.) Hunt	Nogmung, Kachin; Seinghku taung, Northern Myanmar	Kingdon-Ward 7007 (K!), Kingdon-Ward 9678 (BM)
539	*Galeola lindleyana* Rchb. f.	Putao, Kachin; Chin	Xiaohua Jin et al. PT-2293 (PE!); Forrest 26846 & 29734 (E!); Farrer 1107 (E!)
540	*Galeola nudifolia* Lour.	Mon, Tanintharyi	Kress et al. 2003
541	*Gastrochilus acutifolius* (Lindl.) Kuntze	Reported from Myanmar	Kress et al. 2003
542	*Gastrochilus bellinus* (Rchb. f.) Kuntze	Recorded from Myanmar	s.coll. s.n. (E!)
543	*Gastrochilus calceolaris* D. Don	Putao, Kachin; Chin; Rakhine; Tanintharyi; Yangon	Xiaohua Jin et al. PT-6800 (PE!)
544	*Gastrochilus distichus* (Lindl.) Kuntze	Mt. Victoria, Chin; Kachin	Xiaohua Jin et al. PT-5397 (PE!), Ye Lwin Aung PT-7478 (PE!); Unwin 3068 (E!); Kingdon-Ward 21930 (BM!)
545	*Gastrochilus intermedius* (Griff. ex Lindl.) Kuntze	Putao, Kachin; Tanintharyi	Xiaohua Jin et al. PT-6917 (PE!)
546	*Gastrochilus obliquus* (Lindl.) Kuntze	Putao, Kachin; Kyaukme, Shan	Kate Armstrong 2282 & 3077 (NY!); McMillen 144 (US); Wallich 7304 (AMES!)
547	***Gastrochilus pechei* Schltr.**	Mawlamyaing (Moulmein), Mon; Tanintharyi	Peché 40811 (W!); s.coll. s.n. (K!)
548	*Gastrochilus platycalcaratus* (Rolfe) Schltr.	Upper Myanmar	Sander & Sono 100-09 (K!); s.coll. s.n. (K!)
549	*Gastrochilus pseudodistichus* (King & Pantl.) Schltr.	Listed as occurrence in Myanmar	Kurzweil and Lwin 2014
550	*Gastrochilus sukhakulii* Seidenf.	Reported from Myanmar	Kress et al. 2003
551	*Gastrodia elata* Blume	Listed as occurrence in Myanmar	Kurzweil and Lwin 2014
552	***Gastrodia kachinensis* X.H.Jin & L.A.Ye**	Hponkanrazi Wildlife Sanctuary, Putao Township, Kachin State	Xiaohua Jin et al. PT -6897 (Putao expedition team 6897) (Holotype, PE!)
553	*Gastrodia menghaiensis* Z.H.Tsi & S.C.Chen	Listed as occurrence in Myanmar	Kurzweil and Lwin 2014
554	***Gastrodia putaoensis* X.H.Jin**	Hkakaborazi National Park, Putao District, Kachin State	Xiaohua Jin et al. PT-2275 (holotype: PE!; isotypes: PE!, IBSC!)
555	*Geodorum densiflorum* (Lam.) Schltr.	Mt. ZweKaBin, Kayin; Bago; Mt. Popa, Mandalay	Xiaohua Jin et al. PT-2566 (PE!); Kingdon-Ward 17464 (NY!); Tanaka et al. 020328 (MBK)
556	*Geodorum eulophioides* Schltr.	Mt. Popa, Mandalay	Khin Myo Htwe 55 (spirit collection-TNS), Tsujita 036208 (spirit collection-TNS)
557	*Geodorum recurvum* (Roxb.) Alston	Kachin; Mt. Popa, Mandalay; Shan	Mokim s.n. (BM!); Tanaka et al. 020437 (MBK); Khin Myo Htwe 41 & 42 (spirit collection-TNS); Khin Myo Htwe 109 (spirit collection-MBK, TNS)
558	*Geodorum siamense* Rolfe ex Downie	Upper Myanmar	Forrest 9172 (E!); Rogers s.n. (E!)
559	*Geodorum terrestre* (L.) Garay	PyinOoLwin (Maymyo), Mandalay; Moulmein, Mon	Parish 180 & 275 (K!); Lace s.n. (E!)
560	*Goodyera foliosa* (Lindl.) Benth. ex C.B.Clarke	Mountain at the East of Fort Hertz, Putao, Kachin	Xiaohua Jin et al. PT-ET 835, PT-ET 841, PT-ET 569, PT-ET 530, PT-2220 (PE!); Kate Armstrong 2094 (NY!); Griffith s.n. (K!); s.coll. s.n. (K!); Kingdon-Ward 10138 & 13271 (BM!)
561	*Goodyera fumata*Thwaites	Keng Tung, Shan	Robertson 306 (K!)
562	*Goodyera fusca* (Lindl.) Hook.f.	Northeast Upper Myanmar	Forrest 27452 (K!) & (E!) & (BM!)
563	*Goodyera hemsleyana* King & Pantl.	Chipwi, Kachin	Kingdon-Ward 1897 (E!)
564	*Goodyera marginata* Lindl.	Recorded from Myanmar	Kingdon-Ward 1879 (E!)
565	***Goodyera myanmarica* Ormerod & C.S.Kumar**	Mountains at the East of Fort Hertz, Putao Township, Kachin State	Kingdon-Ward 7368 (holotype, K)
566	*Goodyera pendula* Maxim.	Putao, Kachin	Xiaohua Jin et al. PT-ET 842 & PT-2287 (PE!)
567	*Goodyera procera* Hook.	Bago; Chin; Putao, Kachin; Shan	Xiaohua Jin et al. PT-6401 (PE!); Kingdon-Ward 24127 (E!); Kingdon-Ward 20593 & 11239 (BM!)
568	*Goodyera repens* (L.) R.Br.	Putao, Kachin	Xiaohua Jin et al. PT-ET 863, PT-2181 (PE!); Kingdon-Ward 13407 & 13161 (BM!)
569	*Goodyera schlechtendaliana* Rchb. f.	Putao, Kachin	Kate Armstrong 1943 (NY!)
570	*Goodyera viridiflora* (Blume) Lindl. ex Dietr.	Listed as occurrence in Myanmar	Kurzweil and Lwin 2014
571	*Grammatophyllum speciosum* Blume	Tanintharyi	Griffith 5318 (P!)
572	*Grosourdya appendiculata* (Blume) Rchb. f.	Reported from Myanmar	Kress et al. 2003
573	*Gymnadenia orchidis* Lindl.	Upper Myanmar	Farrer 1118 (E!); Kingdon-Ward 3294 (E!)
574	*Habenaria acuifera* Wall.	Chin; Shan; Tanintharyi	MacGregor 814 (E!); Prazer 169 (BM!)
575	*Habenaria aitchisonii* Rchb. f.	Recorded from Myanmar	s.coll. s.n. (E!)
576	*Habenaria arietina* Hook. f.	Chin, Shan	MacGregor 817 (E!)
577	*Habenaria avana* Hook. f.	Reported from Myanmar	Kress et al. 2003
578	*Habenaria chlorina* Parish & Rchb. f.	Yangon; Moulmein, Mon	Parish 124 & 245 (K!)
579	*Habenaria commelinifolia* Wall.	Kachin; Shan; Yangon	MacGregor 690 (E!); Mokim 131 (US)
580	***Habenaria corticicola* W.W. Sm.**	Reported from Myanmar	Kress et al. 2003
581	*Habenaria corymbosa* Parish & Rchb. f.	Recorded from Myanmar	Parish 329 (K!)
582	*Habenaria delavayi* Finet	Reported from Myanmar	Kress et al. 2003
583	*Habenaria dentata* (Sw.) Schltr.	Chin; Mt. Popa & PyinOoLwin (Maymyo), Mandalay; Kalaw, Shan	Xiaohua Jin et al. PT-5114 (PE!); Lace 4322 (E!); MacGregor 815 (E!); Keenan et al. 1582 (E!); Kingdon-Ward 3556 (E!); Roger 648 (E!); Kingdon-Ward 22707 (BM!); Khin Myo Htwe 71 (spirit collection-MBK, TNS)
584	*Habenaria dentirostrata* Tang & F.T. Wang	Taunggyigon Reserve, Meikhtila, Mandalay	Maung Tha Myaing 263 (K!) & (E!)
585	*Habenaria digitata* Lindl.	Mt. Popa, Mandalay; Tanintharyi	Khin Myo Htwe 50 (spirit collection-TNS)
586	*Habenaria diphylla* (Nimmo) Dalzell	Shan; Dawei (Tavoy), Tanintharyi	MacGregor 681 (E!); Keenan et al. 933, 758 & 905 (E!)
587	***Habenaria ditricha* Hook. f.**	Putao, Kachin; Moulmein, Mon	Lobb 350 (K!); Kate Armstrong 1649 (NY!)
588	*Habenaria fulva* Tang & F.T. Wang	Reported from Myanmar	Kress et al. 2003
589	*Habenaria furcifera* Lindl.	Chin, Yangon	Kress et al. 2003
590	*Habenaria humidicola* Rolfe	Listed as occurrence in Myanmar	Kurzweil and Lwin 2014
591	*Habenaria intermedia* D. Don	Chin	U Mg Gale-2 & U Chit Ko Ko 5595 (RAF), Yasuda 060067 (MBK), Tanaka & Yukawa 081134 (MBK), Tanaka & Yukawa 081279, 081398 (MBK), Mu Mu Aung et al. 092340 (MBK)
592	*Habenaria limprichtii* Schltr.	Recorded from Myanmar	Kingdon-Ward 1668 (E!)
593	*Habenaria lindleyana* Steud.	Reported from Myanmar	Kress et al. 2003
594	***Habenaria linearis* King & Pantl.**	Reported from Myanmar	Kress et al. 2003
595	*Habenaria linguella* Lindl.	Loilem District, Shan State	Robertson 7 (K)
596	*Habenaria lucida* Wall. ex Lindl.	Bago, Mon, Tanintharyi, Yangon, Mt. Popa of Mandalay Region	Murata et al. 021000 (MBK), Khin Myo Htwe 61 (spirit collection-TNS)
597	*Habenaria malintana* (Blanco) Merr.	Mon, Tanintharyi, Mt. Popa of Mandalay Region	Tanaka et al. 020764 (MBK)
598	*Habenaria malleifera* Hook. f.	Reported from Myanmar	Kress et al. 2003
599	*Habenaria mandersii* Collett & Hemsl.	Upper Myanmar	Manders s.n. (K!)
600	*Habenaria marginata* Colebr.	Inle, Shan	Robertson 428 (K!)
601	***Habenaria massoniana* King & Pantl.**	Reported from Myanmar	Kress et al. 2003
602	*Habenaria medioflexa* Turrill	Mt. Popa, Mandalay	Tanaka et al. 020761 (MBK, TNS)
603	***Habenaria mientienensis* Tang & F.T. Wang**	Sakangyi, Mandalay	Mya 3658 (K!)
604	*Habenaria myriotricha* Gagnep.	Dawei (Tavoy), Tanintharyi	Keenan et al. 771, 887, 1379 & 841 (E!)
605	*Habenaria pantlingiana* Kraenzl.	Mindat, Chin; Shan	Kingdon-Ward 22580 (BM!); Seo et al. 100743 (MBK)
606	*Habenaria pectinata* D. Don	Mindat, Chin; Mandalay	Kingdon-Ward 22357 (BM!)
607	*Habenaria plurifoliata* Tang & F.T.Wang	Putao, Kachin	Xiaohua Jin Jin-14581 (PE!)
608	***Habenaria prazeri* King & Pantl.**	Reported from Myanmar	Kress et al. 2003
609	*Habenaria reflexa* Blume	Mt. Popa, Mandalay	Khin Myo Htwe 67 (spirit collection-MBK)
610	*Habenaria reniformis* (D.Don) Hook.f.	Kyaukpadaung Township, Mandalay Region	Saw Lwin et al. MPO 020 (SING)
611	*Habenaria rhodocheila* Hance	Mindat & Falam, Chin; Pyin Oo Lwin (Maymyo), Mogok & Mt. Popa, Mandalay	Rodger 201 (K!); Kingdon-Ward 22700 (BM!); Daun 98 (K); Samuel 13582 (K); Khin Myo Htwe 024088A (MBK)
612	*Habenaria rostrata* Wall. ex Lindl.	Bago, Tanintharyi	Wallich s.n. (GH!) & (P!)
613	***Habenaria shweliensis* W.W. Sm. & Banerji**	Mogok, Mandalay	Rodger 387 (K!); Lace s.n. (E!)
614	***Habenaria spatulifolia* Parish & Rchb. f.**	Tanintharyi	Parish 217 (K!)
615	*Habenaria stenopetala* Lindl.	Mt. Popa, Mandalay	Khin Myo Htwe 66 (spirit collection-TNS)
616	*Habenaria tonkinensis* Seidenf.	Putao, Kachin	Xiaohua Jin et al. PT-2545 (PE!)
617	*Habenaria trichosantha* Wall.	Mon	Kress et al. 2003
618	***Habenaria triquetra* Rolfe**	Reported from Myanmar	Kress et al. 2003
619	*Habenaria vidua* Parish & Rchb. f.	Mon, Tanintharyi	Parish 223 (K!)
620	*Habenaria viridiflora* (Rottler ex Sw.) Lindl.	Mt. Popa, Mandalay	Kress et al. 2003
621	***Habenaria yomensis* Gage**	Reported from Myanmar	Kress et al. 2003
622	*Habenaria yuana* Tang & Wang	Mt. Victoria, Chin; Mt. Popa, Mandalay	Tanaka & Yukawa 081279 (MBK), Khin Myo Htwe 65 (spirit collection-TNS)
623	*Hemipilia calophylla* Parish & Rchb. f.	Putao, Kachin; PyinOoLwin (Maymyo), Mandalay; Gokteik, Lashio, Shan; Moulmein, Mon	Xiaohua Jin et al. PT-2569 (PE!); s.coll. 13507 (K!); J.H. Lace s.n. (K!); Parish 348 (K!); Kingdon-Ward 22358 (BM!)
624	*Hemipilia cordifolia* Lindl.	Mt. Victoria & Haka, Chin; Shan	Venning 18 (K!); Cooper 6048 (E!)
625	*Hemipilia limprichtii* Schltr.	PyinOoLwin (Maymyo), Mandalay	R.C.F. Swinhoe 67 (K!)
626	*Herminium edgeworthii* (Hook.f. ex Collett) X.H.Jin, Schuit., Raskoti & Lu Q.Huang	Listed as occurrence in Myanmar	Kurzweil and Lwin 2014
627	*Herminium elisabethae* (Duthie) Tang & F.T.Wang	Listed as occurrence in Myanmar	Kurzweil and Lwin 2014
628	*Herminium fallax* (Lindl.) Hook.f.	Chin	Yasuda 053610A (MBK), Yasuda 060088 (spirit collection-MBK), Yasuda 053613 (MBK, spirit collection MBK), Mu Mu Aung et al. 092824 (MBK), Fujikawa et al. 094119 (MBK)
629	*Herminium forceps* (Finet) Schltr.	Shan	Kilgour et al. m-760 (MBK)
630	*Herminium kamengense* A.N. Rao	Chin	Kress et al. 2003
631	*Herminium lanceum (Thunb. ex Sw.) Vuijk*	Chin; Kachin; Mt. Popa, Mandalay	Ye Lwin Aung PT-7224 (PE!); Forrest 27084 (E!); Farrer 995 (E!); Kingdon-Ward 1662 (E!); Kingdon-Ward 22612 (BM!); Murata et al. 021048 (MBK)
632	*Herminium latilabre* (Lindl.) X.H.Jin, Schuit., Raskoti & Lu Q.Huang	Haka, Chin	Venning 62 (K!); Kingdon-Ward 22671 (BM!)
633	*Herminium mannii* (Rchb.f.) Tang & F.T.Wang	Reported from Myanmar	Kress et al. 2003
634	*Herminium souliei* (Finet) Rolfe	Shan	Seo et al. 100687 (MBK)
635	*Herminium quinquelobum* King & Pantl.	Mt. Victoria, Chin	X.H. Jin 24588 (PE!)
636	*Herpysma longicaulis* Lindl.	Putao, Kachin	Xiaohua Jin et al. PT-ET 215, PT-2075, PT-2263 (PE!); Ye Lwin Aung PT-7039 (PE!); Keenan et al. 3308 (E!); Kate Armstrong 2114 (NY!); Kingdon-Ward 13525 (BM!)
637	*Hetaeria affinis* (Griff.) Seidenf. & Ormerod	Chin; Kachin; Magway; Mandalay; Uyu, Upper Chindwin, Sagaing; Shan; Thaton, Mon	Lulay 9040 (K!); Lace 4606 (K!)
638	*Hetaeria anomala* Lindl.	Recorded from Myanmar	Kingdon-Ward 20633 (BM!)
639	*Hetaeria finlaysoniana* Seidenf.	Listed as occurrence in Myanmar	Kurzweil and Lwin 2014
640	*Hetaeria oblongifolia* (Blume) Blume	Tanintharyi	Helfer 464 (K!)
641	*Holcoglossum amesianum* (Rchb. f.) Christenson	Putao, Kachin; Sagaing; Shan; Mt. Popa, Mandalay	Xiaohua Jin et al. PT-5101 (PE!), Khin Myo Htwe 76 (spirit collection-MBK, TNS)
642	*Holcoglossum himalaicum* (Deb, Sengupta & Malick) Aver.	Putao, Kachin; Valley of the Sunghku, Northernmost Myanmar	Xiaohua Jin et al. PT-2101 (PE!); Kingdon-Ward 7620 (K!); Kingdon-Ward 21528 (BM!)
643	*Holcoglossum kimballianum* (Rchb. f.) Garay	Chin; Kachin; Taunggyi & Kalaw, Shan	Xiaohua Jin et al. PT-5099 (PE!); Ye Lwin Aung PT-7599, PT-7600 (PE!); s.coll. s.n. (E!)
644	*Holcoglossum subulifolium* (Rchb. f.) Christenson	Chin, Kachin, Sagaing, Shan	Kress et al. 2003
645	*Lecanorchis javanica* Blume	Putao, Kachin	Xiaohua Jin et al. PT-ET 1185 B (PE!)
646	*Lecanorchis nigricans* Honda	Putao, Kachin	Ye Lwin Aung PT-7151 (PE!)
647	*Lecanorchis sikkimensis* Pearce & Cribb	Putao, Kachin	Xiaohua Jin et al. PT-2091, PT-2109 (PE!)
648	*Liparis barbata* Lindl.	Lawa, 85.30 km west of Myitkyina & Putao, Kachin	Xiaohua Jin et al. PT-6953 (PE!); H.S.M. Lee 6284 (K!)
649	*Liparis bistriata* Parish & Rchb. f.	Putao & Myitkyina, Kachin; Tanintharyi	Ye Lwin Aung PT-7074 (PE!); Su Koe 10109 (K!); Parish 80 (K!)
650	*Liparis bootanensis* Griff.	YePhyu, Tanintharyi	Ye Lwin Aung PT-7352 (PE!); Parish 233 (K!)
651	*Liparis cespitosa* (Lam.) Lindl.	Putao, Kachin; YePhyu, Tanintharyi	Xiaohua Jin et al. PT-ET 567 (PE!); Ye Lwin Aung PT-7266, PT-7089 (PE!); Kingdon-Ward 3338 & 1771 (E!)
652	*Liparis chapaensis* Gagnep.	Reported from Myanmar	Kress et al. 2003
653	*Liparis condylobulbon* Rchb. f.	Mon	Kress et al. 2003
654	*Liparis deflexa* Hook. f.	Falam & Mindat, Chin; Upper Chindwin, Sagaing	Daun 92 (K!); Lace 4301 (K!) & (E!); Abdul Huk s.n. (K!); Kingdon-Ward 22563 (BM!)
655	*Liparis distans* C.B.Clarke	border regions of Putao and Nogmung Townships, Kachin State	Xiaohua Jin et al. PT-5270 (PE!), Kurzweil H & Saw Lwin KL 2460 (herbarium of Myanmar Floriculturist Association)
656	*Liparis downii* Ridl.	Reported from Myanmar	Kress et al. 2003
657	*Liparis elliptica* Wight	Putao, Kachin	Xiaohua Jin et al. PT-6481 (PE!); Kingdon-Ward 9174, 9127 & 10178 (BM!)
658	***Liparis forrestii* Rolfe**	Reported from Myanmar	Kress et al. 2003
659	*Liparis grossa* Rchb. f.	Reported from Myanmar	Kress et al. 2003
660	*Liparis jovispluvii* Parish & Rchb. f.	Mindat, Chin; Mon; Paungdaw Power station, Tanintharyi	Keenan et al. 885 (K!) & (E!); Parish 323 (K!); Kingdon-Ward 22517 & 22466 (BM!)
661	*Liparis lacerata* Ridl.	Tanintharyi	Griffith 5073 (K!)
662	*Liparis luteola* Lindl.	Chin, Kachin, Magway, Mandalay, Mon, Sagaing, Shan, Tanintharyi	Kress et al. 2003
663	*Liparis mannii* Rchb.f.	Putao & Tanai Townships, Kachin State	Xiaohua Jin et al. PT-6295 (PE!); Murata et al. 041223 (MBK)
664	*Liparis nervosa* (Thunb.) Lindl.	Putao, Kachin; Magway; Mon; Shan; Tanintharyi	Ye Lwin Aung PT-7125, PT-7145 (PE!); s.coll. 01007 (K!);
665	*Liparis odorata* (Willd.) Lindl.	Mindat, Chin; Kachin; Mandalay; Sagaing; Lwelin Hlaing Hokmein taung, Shan; Tanintharyi	Swinhoe 58 (K!); Mokim 23 (K!) & (BM!) & (US); Parish 317 (K!); Maung Po Khant 16327 & 16350 (E!); Kingdon-Ward 22516 & 22517 (BM!)
666	*Liparis petiolata* (D. Don) P.F. Hunt & Summerh.	Chipwi Township, Kachin State	Kingdon-Ward 1838 (E!)
667	*Liparis plantaginea* Lindl.	Nogmung or Putao Township, Kachin State	Kress et al. 2003
668	***Liparis popaensis* X.H.Jin, A.T.Mu & L.A.Ye**	Mt. Popa, Mandalay	Xiaohua Jin et al. 19984 (holotype, PE!; isotype, RAF!)
669	*Liparis regnieri* Finet	Mt. Popa & PyinOoLwin (Maymyo), Mandalay; Upper Myanmar	Tsujita et al. 036188 (spirit collection-TNS); J.H. Lace s.n. (K!); D. Burlee s.n. (K!); s.coll. 13576 (K!)
670	*Liparis resupinata* Ridl	Waingmaw Township, Kachin State	Stephen Lasi Bawk Naw BW 32 (herbarium of the Myanmar Floriculturist Association)
671	*Liparis siamensis* Rolfe ex Downie	Mt. Popa, Mandalay	Ye Lwin Aung PT-7225 (PE!)
672	***Liparis stenoglossa* Parish & Rchb. f.**	Recorded from Myanmar	Parish 154 (K!)
673	*Liparis stricklandiana* Rchb.f.	Putao, Kachin	Ye Lwin Aung PT-7016, PT-7075, PT-7083 (PE!)
674	*Liparis torta* Hook. f.	Reported from Myanmar	Kress et al. 2003
675	*Liparis tschangii* Schltr.	Lwelin Hlaing Hokmein taung, Shan; Pyay, Bago	Maung Po Khant 16326 (E!); Sugawara et al. 036384 (MBK)
676	*Liparis viridiflora* (Blume) Lindl.	Ayeyarwaddy; Zamani Reserve, Bago; Putao, Kachin; Kayah; Kayin; Mandalay; Mon; Hkamti, Sagaing; Shan; YePhyu, Tanintharyi	Xiaohua Jin et al. PT-5261 (PE!); Ye Lwin Aung PT-7173, PT-7176, PT-7262, PT-7340, PT-7013 (PE!); Lace s.n. (E!); Kate Armstrong 2230 & 3193 (NY!); Kingdon-Ward 288 (NY!); Egerod B-109 (US)
677	*Ludisia discolor* (Ker Gawl.) Lindl.	Reported from Myanmar	Kress et al. 2003
678	***Luisia amesiana* Rolfe**	Shan	Garden Collector 583 (K!)
679	*Luisia brachystachys* Blume	Mt. Victoria, Chin; Paunglaung Creek, Nay Pyi Taw-Shan Yoma	Ye Lwin Aung PT-7385, PT-7489, PT-7543 (PE!); Batten s.n. (BM!)
680	***Luisia cantharis* Rolfe**	PyinOoLwin (Maymyo), Mandalay; Shan	Bogg B.G. 8 (K!); s.coll. s.n. (K!)
681	*Luisia filiformis* Hook.f.	Mt. Victoria, Chin; Alaungdaw Kathapa National Park, Sagaing	Ye Lwin Aung PT-7422, PT-7568 (PE!)
682	*Luisia hancockii* Rolfe	Putao, Kachin; Alaungdaw Kathapa National Park, Sagaing	Ye Lwin Aung PT-7399, PT-7057 (PE!)
683	*Luisia macrotis* Rchb.f.	Mt. Popa, Mandalay	Khin Myo Htwe 26 (spirit collection-TNS)
684	*Luisia magniflora* Tsi & Chen	Listed as occurrence in Myanmar	Kurzweil and Lwin 2014
685	***Luisia primulina* Parish & Rchb. f.**	Moulmein, Mon; Tanintharyi	Peche s.n. (K!)
686	*Luisia psyche* Rchb. f.	Mon; Tanintharyi; Mt. Popa, Mandalay	Maxwell JF 98- 1303 (L!); s.coll. s.n. (K!); Tanaka et al. 036142 (MBK, spirit collection-TNS), Tsujita et al. 036167 (spirit collection-TNS)
687	*Luisia thailandica* Seidenf.	Reported from Myanmar	Kress et al. 2003
688	*Luisia trichorrhiza* (Hook) Blume	PyinOoLwin (Maymyo), Mandalay	A. Samuel 13558 (K!)
689	*Luisia volucris* Lindl.	Reported from Myanmar	Kress et al. 2003
690	*Luisia zeylanica* Lindl.	Mt. Popa, Mandalay; Bago; Kachin; Tanintharyi; Yangon	Khin Myo Htwe 17 (spirit collection-TNS), Khin Myo Htwe 035060 (MBK), Tanaka et al. 036242 (spirit collection-MBK, TNS) & 036252 (spirit collection-TNS), Khin Myo Htwe 93 (spirit collection-MBK)
691	*Luisia zollingeri* Rchb. f.	Reported from Myanmar	Kress et al. 2003
692	*Malaxis muscifera* (Lindl.) Kuntze	Chin, Tanintharyi	Farrer 1117 (E!); Kingdon-Ward 1769 (E!); Kingdon-Ward 9758 (BM!)
693	*Malaxis versicolor* (Lindl.) Abeywickr.	Reported from Myanmar	Kress et al. 2003
694	*Micropera obtusa* (Lindl.) Tang & F.T. Wang	Putao, Kachin; Mon; Tanintharyi	Ye Lwin Aung PT-7053 (PE!)
695	*Micropera pallida* (Roxb.) Lindl.	Putao, Kachin; Tanintharyi	Xiaohua Jin et al. PT-2072, PT-2378 (PE!); A.F.G. Kerr 01000 (K!) & (P!)
696	*Micropera rostrata* (Roxb.) N.P. Balakr.	Reported from Myanmar	Kress et al. 2003
697	***Micropera secunda* (Rolfe) T. Tang & F.T. Wang**	Recorded from Myanmar	s.coll. s.n. (K!)
698	*Micropera thailandica* (Seidenf. & Smitinand) Garay	YePhyu, Tanintharyi	Saw Lwin et al. TNRO 61 (SING, SING [spirit], herbarium of Myanmar Floriculturist Association)
699	*Microsaccus griffithii* (Parish & Rchb. f.) Seidenf.	Reported from Myanmar	Kress et al. 2003
700	*Mycaranthes floribunda* (D.Don) S.C.Chen & J.J.Wood	Putao, Kachin; Naga hill, Sagaing; Mandalay	Xiaohua Jin et al. PT-2069 (PE!), Ye Lwin Aung PT-7103, PT-7126 (PE!); D. Prain 67 (K!); Kingdon-Ward 10208 (BM!)
701	*Mycaranthes oblitterata* Blume	Putao, Kachin	Ye Lwin Aung PT-7081 (PE!)
702	*Mycaranthes pannea* (Lindl.) S.C.Chen & J.J.Wood	Putao, Kachin; Mt. Victoria, Chin; Upper Myanmar; Tanintharyi	Xiaohua Jin et al. PT-2049 (PE!), Ye Lwin Aung PT-7049, PT-7121, PT-7183, PT-7191, PT-7566 (PE!); Daun 67 (K!); G. Forrest 25121 (K!)
703	*Myrmechis drymoglossifolia* Hayata	Putao, Kachin	Xiaohua Jin et al. PT-2096 & PT-2224 (PE!)
704	*Myrmechis pumila* (Hook.f.) Tang & Wang	Laktang, Putao, Kachin	Kingdon-Ward 3337 & 1722 (E!); Kingdon-Ward 21107 (BM!)
705	*Neogyna gardneriana* (Lindl.) Rchb.f.	Putao, Kachin	Xiaohua Jin et al. PT-ET 1026 (PE!); s.coll. s.n. (E!); Kingdon-Ward 9109 (BM!)
706	*Neottia dentata* (King & Pantl.) Szlach.	Tama Bum, North Triangle of Kachin State	Kingdon-Ward 21058 (AMES, BM)
707	***Neottia flabellata* (W.W.Sm.) Szlach.**	Valley of the Chawng-maw-hka, Upper Myanmar	Kingdon-Ward 3414 (E!) & (BM!); Kingdon-Ward 21085 (BM!)
708	*Neottia karoana* Szlach.	Putao & Nogmung, Kachin	Xiaohua Jin et al. PT-2122, PT-2223 (PE!), Kingdon-Ward 9961 (AMES), Kingdon-Ward 3359 (E)
709	*Neottia longicaulis* (King & Pantl.) Szlach.	Listed as occurrence in Myanmar	Kurzweil and Lwin 2014
710	*Neottia pinetorum* (Lindl.) Szlach.	Chipwi Township, Kachin State	Kingdon-Ward 1631 & 1652 (E!)
711	***Neottia unguiculata* (W.W.Sm.) Szlach.**	Hpimaw Pass & Laktang, Kachin	Kingdon-Ward 3350 & 1875 (E!)
712	*Nephelaphyllum cordifolium* (Lindl.) Blume	Nogmung Township, Kachin State	Kingdon-Ward 7314 (K)
713	*Nephelaphyllum pulchrum* Blume	Kachin	Keenan et al. 3015 (E!)
714	*Nephelaphyllum tenuiflorum* Blume	Putao, Kachin	Ye Lwin Aung PT-7120 (PE!)
715	*Nervilia concolor* (Blume) Schltr.	Mt. Popa, Mandalay	Kingdon-Ward 22208 (BM!); Than Than Aye & Khin Myo Htwe 020725 (MBK), Khin Myo Htwe 024044 (MBK)
716	*Nervilia juliana* (Roxb.) Schltr.	Listed as occurrence in Myanmar	Kurzweil and Lwin 2014
717	*Nervilia khasiana* (King & Pantl.) Schltr.	Reported from Myanmar	Kress et al. 2003
718	*Nervilia macroglossa* (Hook. f.) Schltr.	Kachin	Kress et al. 2003
719	*Nervilia maculata* (Parish & Rchb. f.) Schltr.	PyinOoLwin (Maymyo), Mandalay; Mon; Tanintharyi	Parish 165 (K!); Rodger 572 (E!)
720	*Nervilia plicata* (Andrews) Schltr.	Mt. Zwekabin, Kayin; Mandalay; Mon; Tanintharyi	Xiaohua Jin et al. PT-2565 (PE!); Kingdon-Ward 486 (NY!); Kingdon-Ward 22166 (BM!)
721	*Oberonia acaulis* Griff.	Putao, Kachin; YePhyu, Tanintharyi	Xiaohua Jin et al. PT-2028, PT-5252 (PE!); Ye Lwin Aung PT-7347 (PE!); Kingdon-Ward 1862 (E!)
722	*Oberonia angustifolia* Lindl.	YePhyu, Tanintharyi	Ye Lwin Aung PT-7344 (PE!)
723	*Oberonia anthropophora* Lindl.	Putao, Kachin; Mt. Victoria, Chin; Dawei (Tavoy), Tanintharyi	Xiaohua Jin et al. PT-5220 (PE!); Ye Lwin Aung PT-7066, PT-7079, PT-7102 (PE!); Keenan et al. 1457 (E!)
724	*Oberonia bantaengensis* J.J.Sm.	YePhyu, Tanintharyi	Ye Lwin Aung PT-7289 (PE!)
725	*Oberonia brachystachys* Lindl.	Tanintharyi	s.coll. s.n. (GH!)
726	*Oberonia brunoniana* Wight	Reported from Myanmar	Kress et al. 2003
727	*Oberonia caulescens* Lindl.	Valley of Dichu, Kachin	Kingdon-Ward 7169 (K!); Kingdon-Ward 22783 (BM!)
728	*Oberonia cavaleriei* Finet	Chin, Mon	Kress et al. 2003
729	*Oberonia ensiformis* Lindl.	Mt. Popa & Headwater of Satthwa Chaung, Mandalay	Maung Hla 3638 (K!); Khin Myo Htwe 74 (spirit collection-TNS)
730	*Oberonia evrardii* Gagnep.	Shan	MacGregor 822 (E!)
731	*Oberonia falcata* King & Pantl.	YePhyu, Tanintharyi	Ye Lwin Aung PT-7282 (PE!)
732	*Oberonia falconeri* Hook. f.	Mt. Victoria, Chin; Alaungdaw Kathapa National Park, Sagaing; PyinOoLwin (Maymyo), Mandalay	Ye Lwin Aung PT-7406, PT-7514 (PE!); A. Samuel 13516 (K!)
733	*Oberonia ferruginea* Parish	Putao, Kachin; Mandalay; Mon; Tanintharyi	Xiaohua Jin et al. PT-2379 (PE!); s.coll. 288 (K!)
734	*Oberonia forcipata* Lindl.	Mt. Victoria, Chin	Ye Lwin Aung PT-7511 (PE!)
735	*Oberonia gammiei* King & Pantl.	Mt. Victoria, Chin	Ye Lwin Aung PT-7510 (PE!)
736	*Oberonia griffithiana* Lindl.	Putao, Kachin; Bago; Mon; Tanintharyi	Xiaohua Jin et al. PT-2259 (PE!); s.coll. 826 (K!); Griffith 5090 (K!)
737	*Oberonia helferi* Hook.f.	Tanintharyi	Helfer 5088 (K!)
738	*Oberonia heliophila* Rchb.f.	YePhyu, Tanintharyi	Ye Lwin Aung PT-7343 (PE!)
739	*Oberonia insectifera* Hook.f.	Mt. Victoria, Chin	Ye Lwin Aung PT-7513 (PE!)
740	*Oberonia jenkinsiana* Griff. ex Lindl.	Listed as occurrence in Myanmar	Kurzweil and Lwin 2014
741	*Oberonia lycopodioides* (Koenig) Ormerod	Tanintharyi	Rottler s.n. (K!)
742	*Oberonia mannii* Hook. f.	Putao, Kachin	Xiaohua Jin et al. PT-2107, PT-2278 (PE!), Ye Lwin Aung PT-7023 (PE!)
743	*Oberonia maxima* Parish	Mon; Alaungdaw Kathapa National Park, Sagaing; Tanintharyi	Ye Lwin Aung PT-7446 (PE!); Parish 287 (K!)
744	*Oberonia mucronata* (D. Don) Ormerod & Seidenf.	Mon, Shan, Tanintharyi	Kress et al. 2003
745	*Oberonia obcordata* Lindl.	Putao Township, Waingmaw Township, Kachin State	Kurzweil, H. & Saw Lwin KL 2664 (SING), Stephen Lasi Bawk Naw BW 8 (RAF, herbarium of the Myanmar Floriculturist Association).
746	*Oberonia pachyrachis* Rchb.f. ex Hook.f.	Mt. Victoria, Chin	Ye Lwin Aung PT-7512 (PE!)
747	*Oberonia pyrulifera* Lindl.	Chin	Kress et al. 2003
748	*Oberonia rufilabris* Lindl.	Mon, Tanintharyi	Griffith s.n. (P!); s.coll. s.n. (E!)
749	*Oberonia seidenfadenii* (H.J.Su) Ormerod	Alaungdaw Kathapa National Park, Sagaing	Ye Lwin Aung PT-7414 (PE!)
750	*Odontochilus clarkei* Hook.f.	Listed as occurrence in Myanmar	Kurzweil and Lwin 2014
751	*Odontochilus crispus* (Lindl.) Hook.f.	Listed as occurrence in Myanmar	Kurzweil and Lwin 2014
752	*Odontochilus elwesii* Clarke ex Hook.f.	Listed as occurrence in Myanmar	Kurzweil and Lwin 2014
753	*Odontochilus grandiflorus* (Lindl.) Benth. ex Hook.f.	Putao, Kachin	Ye Lwin Aung PT-7062 (PE!)
754	*Odontochilus lanceolatus* (Lindl.) Blume	Myitkyina & Putao, Kachin	Kaulback 75 & 404 (BM!); Kingdon-Ward 13062 & 21255 (BM!)
755	*Odontochilus poilanei* (Gagnep.) Ormerod	Putao, Kachin	Ye Lwin Aung PT-7063 (PE!)
756	***Odontochilus putaoensis* X.H. Jin, L.A. Ye & A.T. Mu**	Hponkanrazi Wildlife Sanctuary, Putao Township, Kachin State	Xiaohua Jin et al. PT-ET 959 (Holotype, PE!)
757	*Odontochilus tortus* King & Pantl.	Kachin	Kingdon-Ward 21255 (BM!)
758	***Oreorchis aurantiaca* Pearce & Gibbs**	Kachin	Kingdon-Ward 13334 (BM!)
759	*Oreorchis discigera* W.W. Sm.	Chin, Kachin, Magway, Mandalay, Sagaing, Shan	Kingdon-Ward 1640 (E!) & (BM!)
760	*Oreorchis foliosa* (Lindl.) Lindl.	Kachin	Kingdon-Ward 9596 (BM!)
761	*Oreorchis micrantha* Lindl.	Recorded from Myanmar	Forrest 26809 (E!); Kingdon-Ward 1633 (E!)
762	*Oreorchis oligantha* Schltr.	Nogmung Township, Kachin State	Kingdon-Ward 7076 (K)
763	*Otochilus albus* Lindl.	Putao, Kachin; Chin; YePhyu, Tanintharyi	Xiaohua Jin et al. PT-ET 1038 (PE!); Ye Lwin Aung PT-7300, PT-7301 (PE!); Keenan et al. 3556 (E!)
764	*Otochilus fusca* Lindl.	Putao, Kachin; Chin; Kyaikkami (Amherst), Mon	Ye Lwin Aung PT-7085, PT-7097, PT-7109, PT-7154, PT-7207 (PE!); Forrest 26566 (E!) & (US); Kingdon-Ward 22095 (E!) & (BM!); Lace 5648 (E!); Keenan et al. 3816 (E!)
765	*Otochilus lancilabius* Seidenf.	Putao, Kachin; Paunglaung Creek, Nay Pyi Taw-Shan Yoma	Ye Lwin Aung PT-7374 (PE!); Xiaohua Jin et al. PT-ET 574 (PE!); Kate Armstrong 1917 (NY!)
766	*Otochilus porrectus* Lindl.	Mt. Victoria, Chin; YePhyu, Tanintharyi	Ye Lwin Aung PT-7345, PT-7520, PT-7521, PT-7522 (PE!); Kingdon-Ward 9081 & 22245 (BM!)
767	*Oxystophyllum carnosum* Blume	Mon, Tanintharyi	Kress et al. 2003
768	*Pachystoma pubescens* Blume	Myitkyina, Kachin	Lace 5237 (E!); Kingdon-Ward 21786 (BM!)
769	*Panisea apiculata* Lindl.	Mon, Tanintharyi	Kress et al. 2003
770	*Panisea demissa* (D. Don) Pfitzer	Recorded from Myanmar	Kingdon-Ward 21682 (BM!)
771	*Panisea distelidia* Lund	Listed as occurrence in Myanmar	Kurzweil and Lwin 2014
772	*Panisea tricallosa* Rolfe	Putao, Kachin; Mt. Victoria, Chin	Ye Lwin Aung PT-7479 (PE!), Kurzweil & Lwin 2442 (SING [spirit]), Kurzweil & Lwin 2573 (SING)
773	*Panisea uniflora* (Lindl.) Lindl.	Mon; Mt. Popa, Mandalay; Paunglaung Creek, Nay Pyi Taw-Shan Yoma; YePhyu, Tanintharyi	Ye Lwin Aung PT-7284, PT-7349, PT-7371, PT-7379 (PE!), Tanaka et al. 036190 (MBK, TNS), Tanaka et al. 036185 (spirit collection-TNS)
774	*Panisea yunnanensis* S.C.Chen & Z.H.Tsi	Mt. Victoria, Chin	Ye Lwin Aung PT-7547 (PE!)
775	*Paphiopedilum bellatulum* Pfitzer	Meikhtila, Mandalay	Prazer s.n. (K!); Lace s.n. (E!)
776	*Paphiopedilum callosum* (Rchb.f.) Stein	Listed as occurrence in Myanmar	Kurzweil and Lwin 2014
777	*Paphiopedilum charlesworthii* (Rolfe) Pfitzer	Shan, Yangon	s.coll. s.n. (K!)
778	*Paphiopedilum concolor* (Bateman) Pfitzer	Kayin; Kyaikkami (Amherst), Mon; Shan; Tanintharyi	Parish 57 (K!); Bogg B.G. 20 (K!)
779	*Paphiopedilum godefroyae* (Godef.) Pfitzer	Bago, Kayah, Kayin, Mon, Rakhine, Tanintharyi, Yangon	Kress et al. 2003
780	*Paphiopedilum hirsutissimum* (Lindl.) Pfitzer	Reported from Myanmar	Kress et al. 2003
781	*Paphiopedilum insigne* (Wall.) Pfitzer	Chin, Kachin, Mandalay, Sagaing, Shan	Kress et al. 2003
782	***Paphiopedilum myanmaricum* Koop., Iamwir. & S.Laohap.**	Southern Myanmar	Koopowitz et al. 2017
783	*Paphiopedilum niveum* (Rchb.) Pfitzer	Reported from Myanmar	Kress et al. 2003
784	*Paphiopedilum parishii* (Rchb.) Pfitzer	Moulmein, Mon; Kalaw, Shan	Parish 198 (K!); Maymyo Botanic Garden 13512 (K!)
785	*Paphiopedilum spicerianum* (Rchb. f.) Pfitzer	Chin, Kachin	Keenan et al. 3994 (K!) & (E!)
786	*Paphiopedilum tigrinum* Koop. & Haseg.	Listed as occurrence in Myanmar	Kurzweil and Lwin 2014
787	*Paphiopedilum villosum* (Lindl.) Pfitzer	Mt. Victoria, Chin; Kachin; Moulmein & Kyaikkami (Amherst), Mon; Sagaing; Tanintharyi	Ye Lwin Aung PT-7526 (PE!); Lobb 179 (K!); Parish 92 (K!); J.H. Lace 5645 (K!) & (E!); Kingdon-Ward 33 (NY!)
788	*Paphiopedilum wardii* Summerh.	Chin; Putao, Kachin; Mandalay; Sagaing	Xiaohua Jin et al. PT-6388 (PE!); Kingdon Ward 9070 (K!) & (BM!)
789	*Papilionanthe biswasiana* (Ghose & Mukerjee) Garay	Putao, Kachin	Xiaohua Jin et al. PT-6136 (PE!); Kingdon-Ward 21903 (E!); Kingdon-Ward 21903 (BM!)
790	***Papilionanthe sillemiana* (Rchb. f.) Garay**	Reported from Myanmar	Kress et al. 2003
791	*Papilionanthe teres* (Roxb.) Schltr.	Mt. Victoria, Chin; thaton, Mon; YePhyu, Tanintharyi	Xiaohua Jin et al. PT-5179 (PE!); Ye Lwin Aung PT-7239, PT-7326 (PE!); Lace s.n. (E!)
792	*Papilionanthe uniflora* (Lindl.) Garay	Reported from Myanmar	Kress et al. 2003
793	*Papilionanthe vandarum* (Rchb. f.) Garay	Chin	Srisanga et al. 97605 (US)
794	*Pecteilis hawkesiana* (King & Pantl.) Sathish Kumar	Upper Myanmar	Prazer s.n. (K!)
795	*Pecteilis henryi* Schltr.	Naunghkio, Shan	Lace 4854 (K!) & (E!)
796	*Pecteilis ophiocephala* (W.W.Sm.) Ormerod	Laktang, Kachin State	Kingdon-Ward 3336 (E!);
797	*Pecteilis susannae* (L.) Raf.	Kachin; Lwelin Loiyang Main Taung & Kyay Si ManSan, Shan; Mt. Popa, Mandalay	Lace 67 (E!); Maung Po Khant 16320 & 16345 (E!); Mokim 91 (E!); Kingdon-Ward 22695 (BM!); Murata et al. 020761 (MBK)
798	*Pelatantheria ctenoglossum* Ridl.	ALaungdaw Kathapa National Park, Sagaing	Ye Lwin Aung PT-7416 (PE!)
799	*Pelatantheria insectifera* (Rchb. f.) Ridl.	Bago, Tanintharyi	Kress et al. 2003
800	*Pelatantheria rivesii* (Guillaumin) Tang & F.T.Wang	ALaungdaw Kathapa National Park, Sagaing	Ye Lwin Aung PT-7411, PT-7420, PT-7426 (PE!)
801	*Peristylus affinis* (D. Don) Seidenf.	PyinOoLwin (Maymyo), Mandalay	English 160 (E!) & Lace 3227 (E!)
802	*Peristylus constrictus* (Lindl.) Lindl.	Kachin; Chin; Mt. Popa & PyinOoLwin (Maymyo), Mandalay; Sagaing; Shan; Mon; Dawei (Tavoy), Tanintharyi	Swinhoe 45 (K!); Lace 4907(E!); Keenan et al. 574 & 712 (E!); Mokim 14 & 130 (E!) & (US); Rodger 616 (E!); Kaulback 251 (E!); Maung Po Khant 16313 (E!); Tanaka et al. 020246 (MBK); Khin Myo Htwe 68 (spirit collection-TNS)
803	*Peristylus densus* (Lindl.) Santap. & Kapad.	Haka, Chin; Loilan, Shan; PyinOoLwin (Maymyo), Mandalay; Moulmein, Mon; Tanintharyi	Venning 58 (K!); Robertson 380 (K!); Lobb 363 (K!); Lace 3227 (K!); MacGregor 821 (E!)
804	*Peristylus goodyeroides* (D. Don) Lindl.	Mt. Zwekabin, Kayin; Shan; Chin; Mt. Popa & PyinOoLwin (Maymyo), Mandalay; Yangon; Dawei (Tavoy), Tanintharyi	Xiaohua Jin et al. PT-2567 (PE!); Robertson 5 (K!); Daun 93 (K!); Keenan et al. 593 (K!); Lace 4907 & 5297 (E!)
805	*Peristylus gracilis* Blume	Reported from Myanmar	Kress et al. 2003
806	*Peristylus lacertifer* (Lindl.) J.J. Sm.	Tanintharyi	Keenan et al. 811, 1028 & 1814 (E!)
807	*Peristylus parishii* Rchb. f.	Mt. Popa, Mandalay; Shan; Mon	Parish 216 (K!); MacGregor 6802 (E!); Tanaka et al. 036191 (MBK, spirit collection-TNS)
808	*Peristylus prainii* (Hook. f.) Krzl.	Chin; Kachin; MongPaw Loihka, Shan; PyinOoLwin (Maymyo), Mandalay; Sagaing; Tanintharyi; Yangon; Munghoo & Delu valley, Upper Myanmar	Robertson 361 (K!); Swinhoe 50 (K!); Pantling 167 (K!); Kingdon-Ward 8532 (K!); Lace 3227 (E!)
809	*Peristylus tentaculatus* (Lindl.) J.J. Sm.	Reported from Myanmar	Kress et al. 2003
810	*Peristylus tipulifer* (Parish & Rchb. f.) Mukerjee	Dawei (Tavoy), Tanintharyi	Keenan et al. 1191, 1192, 1177, 886 & 985 (E!); Kingdon-Ward 13102 (BM!)
811	*Phaius flavus* (Blume) Lindl.	Upper Myanmar	Kingdon-Ward 1739 (E!)
812	*Phaius mishmensis* (Lindl. & Paxton) Rchb. f.	Chin; Putao, Kachin; Mandalay; Sagaing	Xiaohua Jin et al. PT-ET 1185 A, PT-ET 250 (PE!); Boxall s.n. (K!)
813	*Phaius nanus* Hook.f.	Myitkyina, Kachin	Lace 5236 (E!)
814	*Phaius takeoi* (Hayata) Su	Taunggyi, Shan State	Nyan Tun s.n. (SING)
815	*Phaius tankervilleae* (Aiton) Blume	Khaiyang, Upper Myanmar	Kingdon-Ward 17324 (NY!); Griffith 5292 (P!)
816	*Phaius wallichii* Lindl.	Mt. Popa, Mandalay	Kingdon-Ward 11315 (BM!); Khin Myo Htwe 29 (spirit collection-TNS)
817	*Phalaenopsis cornu-cervi* (Breda) Blume & Rchb. f.	Mandalay, Tanintharyi, Yangon	Mokim 640 (BM!)
818	*Phalaenopsis deliciosa* (Rchb. f.) Sweet	YePhyu, Tanintharyi	Ye Lwin Aung PT-7312 (PE!)
819	*Phalaenopsis difformis* (Wall. ex Lindl.) Kocyan & Schuit.	Putao, Kachin; Mt. Victoria, Chin; PyinOoLwin (Maymyo), Mandalay; Shan; Hkamti, Sagaing; Mon; Dawei (Tavoy), Tanintharyi	Xiaohua Jin et al. PT-5256 (PE!); C.E.P 6155 (K!); Keenan et al. 973 (E!); Kingdon-Ward 1561 (E!); Cooper 6085 (E!); Kate Armstrong 2716 (NY!)
820	*Phalaenopsis hygrochila* J.M.H.Shaw	Mt. Victoria, Chin; Alaungdaw Kathapa National Park, Sagaing; Taunggyi, Shan	Ye Lwin Aung PT-7400, PT-7560, PT-7597 (PE!); s.coll. s.n. (E!)
821	*Phalaenopsis kunstleri* Hook.f.	Listed as occurrence in Myanmar	Kurzweil and Lwin 2014
822	*Phalaenopsis lobbii* (Rchb.) Sweet	Nampakom Drainage, Upper Chindwin; Tanintharyi	Rule(?) 5777 (K!)
823	*Phalaenopsis lowii* Rchb. f.	Mon, Tanintharyi	Parish 125 (AMES!); s.coll. s.n. (AMES!)
824	*Phalaenopsis mannii* Rchb.f.	Listed as occurrence in Myanmar	Kurzweil and Lwin 2014
825	***Phalaenopsis natmataungensis* (T.Yukawa, Nob.Tanaka & J.Murata) Dalstrom & Ormerod**	Mt. Victoria, Chin	Yukawa et al. 029689 (Holotype: TNS; Isotype: MBK, TI)
826	*Phalaenopsis parishii* Rchb.f.	Tanintharyi	Griffith 5236 (K!); Parish 110 (AMES)
827	*Phalaenopsis pulcherrima* (Lindl.) J.J. Sm.	Reported from Myanmar	Kress et al. 2003
828	*Phalaenopsis stobartiana* Rchb. f.	Putao, Kachin	Xiaohua Jin et al. PT-2093 (PE!)
829	*Phalaenopsis sumatrana* Korth. & Rchb.f.	Listed as occurrence in Myanmar	Kurzweil and Lwin 2014
830	*Phalaenopsis taenialis* (Lindl.) Christenson & U.C. Pradhan	Reported from Myanmar	Kress et al. 2003
831	***Pholidota advena* Parish & Rchb. f.**	Mon; Dawei (Tavoy), Tanintharyi	Parish 296 (K!); Keenan et al. 5348 (E!)
832	*Pholidota aidiolepis* Seidenf. & de Vogel	Putao, Kachin	Ye Lwin Aung PT-7058 (PE!)
833	*Pholidota articulata* Lindl.	Putao, Kachin; Mt. Victoria, Chin; Mt. Popa, Mandalay; Paunglaung Creek, Nay Pyi Taw-Shan Yoma; Taunggyi, Shan; Dawei (Tavoy), Tanintharyi	Ye Lwin Aung PT-7069, PT-7086, PT-7138, PT-7386, PT-7533, PT-7548, PT-7549, PT-7550, PT-7581, PT-7582, PT-7583, PT-7534 (PE!); Lugard s.n. (K!); Hooker s.n. (K!); Clarke 11810 (K!); Griffith 5045 (K!); Keenan et al. 1896 (E!); Khin Myo Htwe 24 (spirit collection-TNS), Khin Myo Htwe 45 (MBK), Tanaka et al. 036158 (MBK)
834	*Pholidota chinensis* Lindl.	below Laugyang (Hlawgaw), Myitkyina, Kachin	Lulay 10016 (K!)
835	*Pholidota convallariae* (Parish & Rchb. f.) Hook. f.	Putao, Kachin; Mt. Victoria, Chin; Mandalay; Mulayit & W. Dawna, Kayin; Mon; Tanintharyi	Xiaohua Jin et al. PT-5271 (PE!); F.G. Dickason 7479, 8442 & 8602 (K!); R.C.F. Swinhoe 101 & R 119 (K!)
836	*Pholidota imbricata* Lindl.	Putao, Kachin; Mt. Victoria, Chin; Mt. Popa, Mandalay; Sagaing; Shan; YePhyu, Tanintharyi; Yangon	Xiaohua Jin et al. PT-ET 132 (PE!); Ye Lwin Aung PT-7292, PT-7336, PT-7365, PT-7529 (PE!); Egerod B-20 (US); Rock 7457 (US); Khin Myo Htwe 69 (spirit collection-MBK, TNS)
837	*Pholidota missionariorum* Gagnep.	Listed as occurrence in Myanmar	Kurzweil and Lwin 2014
838	*Pholidota pallida* Lindl.	Kyaikkahmi (Amherst), Mon	Kingdon-Ward 20946 (BM!); Lace s.n. (E!)
839	*Pholidota protracta* Hook. f.	Putao, Kachin; Sagaing	Xiaohua Jin et al. PT-2261, PT-2262 (PE!); Swinhoe K 75 (K!); Kingdon-Ward 21426 (BM!)
840	*Pholidota recurva* Lindl.	Putao, Kachin; Mt. Victoria, Chin; Paungdaw Power Station, Dawei (Tavoy), Tanintharyi	Xiaohua Jin et al. PT-5276, PT-5382 (PE!), Ye Lwin Aung PT-7017, PT-7108, PT-7122, PT-7574, PT-7078 (PE!); Keenan et al. 5389 (K!) & (E!)
841	*Pholidota rubra* Lindl.	Chin, Kachin, Mandalay; Sagaing; Kyaikkami (Amherst), Mon	Xiaohua Jin et al. PT-5253, PT-5343 (PE!); Ye Lwin Aung PT-7019 (PE!); Lace s.n. (E!); Kingdon-Ward 10179 & 13509 (BM!)
842	*Pinalia acervata* (Lindl.) Kuntze	Chin, Mandalay, Tanintharyi	Daun 37 (K!); Forrest 176 (E!)
843	*Pinalia affinis* (Griff.) Ormerod	Listed as occurrence in Myanmar	Kurzweil and Lwin 2014
844	*Pinalia amica* (Rchb.f.) Kuntze	Mt. Victoria, Chin; Kachin; Alaungdaw Kathapa National Park, Sagaing; Tanintharyi	Ye Lwin Aung PT-7429, PT-7504, PT-7507, PT-7430, PT-7431, PT-7559 (PE!); R.C.F. Swinhoe K 74 (K!); Kingdon-Ward 1557 (E!)
845	*Pinalia apertiflora* (Summerh.) A.N.Rao	Listed as occurrence in Myanmar	Kurzweil and Lwin 2014
846	*Pinalia bicolor* (Lindl.) Kuntze	Mt. Victoria, Chin	Ye Lwin Aung PT-7505 (PE!)
847	*Pinalia bractescens* (Lindl.) Kuntze	Bhamo, Kachin; Mandalay; Tanintharyi	Swinhoe 20 & 87 (K!); McMillen 248 (NY!) & (US)
848	***Pinalia brownei* (Braid) Ormerod**	Mt. Popa, Mandalay; Alaungdaw Kathapa National Park, Sagaing; Chin	Ye Lwin Aung PT-7444, PT-7466 (PE!); s.coll. s.n. (K!)
849	*Pinalia concolor* (E.C.Parish & Rchb.f.) Kuntze	Putao, Kachin; Kayin; Mt. Popa, Mandalay; Alaungdaw Kathapa National Park, Sagaing; Taunggyi, Shan; Tanintharyi	Xiaohua Jin et al. PT-5258 (PE!), Ye Lwin Aung PT-7443, PT-7459, PT-7460, PT-7461, PT-7462, PT-7465, PT-7467, PT-7468, PT-7470, PT-7471, PT-7472, PT-7046, PT-7232, PT-7233, PT-7235, PT-7592, PT-7593 (PE!)
850	***Pinalia dasypus* (Rchb.f.) Kuntze**	Upper Myanmar; Tanintharyi	Forrest 26606 (E!); s.n. (K!)
851	*Pinalia densa* (Ridl.) W.Suarez & Cootes	Tanintharyi	Kress et al. 2003
852	*Pinalia eriopsidobulbon* (E.C.Parish & Rchb.f.) Kuntze	Mt. Victoria & Haka, Chin; Mt. Popa, Mandalay; Mon; Alaungdaw Kathapa National Park, Sagaing; Tanintharyi	Ye Lwin Aung PT-7408, PT-7432, PT-7433, PT-7434, PT-7435, PT-7464, PT-7469, PT-7508, PT-7567 (PE!); Venning 17 (K!); Parish 281 (K!); C.E. Parkinson 2459 (K!); R.C.F. Swinhoe 56 (K!)
853	*Pinalia excavata* (Lindl.) Kuntze	Mt. Victoria, Chin	Cooper 6088 (E!)
854	*Pinalia floribunda* (Lindl.) Kuntze	Dawei (Tavoy), Tanintharyi	Keenan et al. 5021 (E!)
855	*Pinalia globulifera* (Seidenf.) A.N. Rao	Putao, Kachin; Mt. Popa, Mandalay	Ye Lwin Aung PT-7011, PT-7012, PT-7022, PT-7037 (PE!)
856	*Pinalia graminifolia* (Lindl.) Kuntze	Upper Myanmar; Kachin	Forrest 26955 & 27242 (K!) & (E!) & (US); Farrer 1109 (E!); Kingdon-Ward 1822 (E!); Forrest 24920 (NY!)
857	*Pinalia lineoligera* (Rchb.f.) Ormerod	Mogok, Mandalay	Kress et al. 2003
858	*Pinalia merguensis* Kuntze	YePhyu & Myeik (Mergui), Tanintharyi	Ye Lwin Aung PT-7250, PT-7257, PT-7258 (PE!); Griffith 1034, 5120 & 5381 (K!); Parish 52 (K!)
859	*Pinalia myristiciformis* (Hook.) Kuntze	Mt. Victoria, Chin; West Dawna, Mon; YePhyu, Tanintharyi	Ye Lwin Aung PT-7541, PT-7251 (PE!); Bogg B.G. 6 (K!); Parish 113 (K!)
860	*Pinalia mysorensis* (Lindl.) Kuntze	Ayeyarwaddy, Rakhine, Tanintharyi, Yangon	Kress et al. 2003
861	*Pinalia obesa* (Lindl.) Kuntze	Bago; Thaton & Kyaikkami (Amherst), Mon; Tanintharyi	Swinhoe 81 (K!); Lace 5595 (K!) & (E!)
862	*Pinalia ovata* (Lindl.) W. Suarez & Cootes	Mt. Victoria, Chin	Ye Lwin Aung PT-7506 (PE!)
863	*Pinalia pachyphylla* (Aver.) S.C.Chen & J.J.Wood	Putao, Kachin	Xiaohua Jin et al. PT-6173, PT-6934 (PE!)
864	*Pinalia praecox* (Aver.) Schuit., Y.P.Ng & H.A.Pedersen	Putao, Kachin	Xiaohua Jin et al. PT-6779 (PE!)
865	*Pinalia pumila* (Lindl.) Kuntze	Mon, Tanintharyi	Kress et al. 2003
866	***Pinalia rimannii* (Rchb.f.) Kuntze**	Reported from Myanmar	Kress et al. 2003
867	***Pinalia shanensis* (King & Pantl.) Ormerod**	Listed as occurrence in Myanmar	Kurzweil and Lwin 2014
868	***Pinalia shiuyingiana* Ormerod & Wood**	Hkrang Hka, Kachin State	Kingdon-Ward 20970 (Holotype: AMES; Isotype: BM[photograph])
869	*Pinalia spicata* (D.Don) S.C.Chen & J.J.Wood	Putao, Kachin; Bago; Chin; PyinOoLwin (Maymyo), Mandalay; Tanintharyi	Xiaohua Jin et al. PT-5355 (PE!); Bogg B.G. 9 (K!); Kingdon-Ward 1847 (E!)
870	*Pinalia stricta* (Lindl.) Kuntze	Putao, Kachin; Mon; Sagaing; Tanintharyi	Ye Lwin Aung PT-7072, PT-7104 (PE!)
871	*Pinalia sutepensis* (Rolfe ex Downie) Schuit., Y.P.Ng & H.A.Pedersen	Mt. Popa, Mandalay	R.C.F. Swinhoe 114 (K!); Khin Myo Htwe 46 (spirit collection-TNS), Tsujita et al. 036172, 036175 (spirit collection-TNS)
872	*Pinalia tenuiflora* (Ridl.) J.J.Wood	Mon, Tanintharyi	Kress et al. 2003
873	*Pinalia trilophota* (Lindl. ex Jackson) Ormerod	Listed as occurrence in Myanmar	Kurzweil and Lwin 2014
874	*Pinalia xanthocheila* (Ridl.) Suarez & Cootes	Recorded from Myanmar	s.n. (K!)
875	*Platanthera angustilabris* Seidenf.	Listed as occurrence in Myanmar	Kurzweil and Lwin 2014
876	*Platanthera bakeriana* (King & Pantl.) Kraenzl.	Adung valley, Putao, Kachin	Kingdon Ward 9806 (AMES), Kingdon Ward 9931 (AMES); Kingdon-Ward 9931 (BM!)
877	*Platanthera bhutanica* K.Inoue	Listed as occurrence in Myanmar	Kurzweil and Lwin 2014
878	*Platanthera calceoliformis* (W.W.Sm.) X.H.Jin, Schuit. & W.T.Jin	Putao, Kachin	Xiaohua Jin et al. PT-2176, PT-2182 (PE!)
879	*Platanthera dulongensis* X.H.Jin & Efimov	Putao, Kachin	Xiaohua Jin et al. PT-2222 (PE!)
880	*Platanthera leptocaulon* (Hook.f.) Soó	Putao, Kachin	Kingdon-Ward 9911 (BM!)
881	***Platanthera longibracteata* Lindl.**	Reported from Myanmar	Kress et al. 2003
882	*Platanthera minutiflora* Schltr.	Putao, Kachin	Kingdon-Ward 9713 & 9803 (BM!)
883	*Platanthera nematocaulon* (Hook.f.) Kraenzl.	Listed as occurrence in Myanmar	Kurzweil and Lwin 2014
884	*Platanthera orbicularis* (Hook.f.) X.H.Jin, Schuit. & Raskoti	Listed as occurrence in Myanmar	Kurzweil and Lwin 2014
885	*Platanthera pachycaulon* (Hook.f.) Soó	Adung valley, Putao, Kachin	Kingdon Ward 9918 (AMES) & (BM!)
886	*Platanthera roseotincta* (W.W. Sm.) Tang & Wang	Kachin	Forrest 26959 (K!) & (E!) & (BM!) & (P!); Kingdon-Ward 3376 (E!); Forrest 25036 (E!); Farrer 1150 (E!); Kingdon-Ward 9764 & 9834 (BM!)
887	*Platanthera stenantha* (Hook.f.) Soo	Putao, Kachin	Forrest 25092 (E!); Farrer 1245 (E!); Kingdon-Ward 1874 (E!); Kingdon-Ward 21458 (BM!)
888	*Platanthera superantha* (J.J.Wood) X.H.Jin, Schuit., Raskoti & Lu Q.Huang	Upper Myanmar; Kachin	Farrer 1125 (E!); Kingdon-Ward 1816 (E!)
889	*Platanthera uniformis* Tang & F.T.Wang	Laktang & Putao, Kachin	Farrer 1910 (E!); Kingdon-Ward 3566 & 1902 (E!); Kate Armstrong 1969 (NY!)
890	*Platanthera urceolata* (Hook.f.) Bateman	Putao, Kachin; valley of Seinghku, Upper Myanmar	Xiaohua Jin et al. PT-2225 (PE!); Kingdon-Ward 7524 (K!); Farrer 1314 (E!); Kingdon-Ward 21427 (E!); Kingdon-Ward 9949, 21427 & 13445 (BM!)
891	*Pleione albiflora* Cribb & C.Z. Tang	Reported from Myanmar	Kress et al. 2003
892	*Pleione forrestii* Schltr.	Chin, Kachin, Sagaing, Shan	Forrest 26656 (NY!) & (BM!) & (P!); Kingdon-Ward 443 (NY!)
893	*Pleione grandiflora* (Rolfe) Rolfe	Recorded from Myanmar	Rock 7512 (US)
894	*Pleione hookeriana* (Lindl.) B.S. Williams	Reported from Myanmar	Kress et al. 2003
895	*Pleione humilis* (J.E. Sm.) D. Don	Putao, Kachin; Mt. Victoria, Chin	Xiaohua Jin et al. PT-ET 862, PT-2120, PT-2265 (PE!); Ye Lwin Aung PT-7515 (PE!); Kingdon-Ward 21966 (BM!)
896	*Pleione limprichtii* Schltr.	Chin	Kress et al. 2003
897	*Pleione maculata* (Lindl.) Lindl.	Putao, Kachin; Chin; Yangon	Keenan et al. 3367 (E!); Kate Armstrong 2172 (NY!); Kingdon-Ward 21515 (BM!)
898	*Pleione praecox* (J.E. Sm.) D. Don	Putao, Kachin; Mt. Victoria, Chin	Xiaohua Jin et al. PT-5205 (PE!); Ye Lwin Aung PT-7490 (PE!); Kate Armstrong 1910 (NY!); s.coll. s.n. (E!); Kingdon-Ward 21508 (BM!)
899	*Pleione scopulorum* W.W. Sm.	Putao, Kachin	Xiaohua Jin et al. 10951 (PE!); Kingdon-Ward 9541 & 21065 (BM!)
900	*Pleione yunnanensis* (Rolfe) Rolfe	Kachin	Farrer 849 (E!)
901	*Podochilus cultratus* Lindl.	Putao, Kachin; Mon; Tanintharyi	Xiaohua Jin et al. PT-ET 135 (PE!), Xiaohua Jin et al. PT-5350 (PE!)
902	*Podochilus khasianus* Hook. f.	Putao, Kachin; Bago; Mon	Xiaohua Jin et al. PT-ET 1233, PT-5249 (PE!), Ye Lwin Aung PT-7172 (PE!)
903	*Podochilus lucescens* Blume	Tanintharyi	Kerr 1004 (P!)
904	*Podochilus microphyllus* Lindl.	Tanintharyi	Kress et al. 2003
905	*Pogonia yunnanensis* Finet	Nogmung, Kachin	Kingdon-Ward 9766 (BM!)
906	*Polystachya concreta* (Jacq.) Garay & Sweet	Mt. Popa, Mandalay	Tanaka et al. 036162 (MBK)
907	*Pomatocalpa diffusum* Breda	Recorded from Myanmar	Griffith 5235 (P!)
908	*Pomatocalpa spicatum* Breda, Kuhl & Hasselt	Tanintharyi	Kress et al. 2003
909	*Ponerorchis chusua* (D.Don) Soó	Myitkyina, Kachin; Seinghku taung, Northern Myanmar	Kingdon-Ward 7060 & 7230 (K!); Naw Mu Pa 17425 (K!); Farrer 1119 (E!); Kingdon-Ward 3448 (E!); Kingdon-Ward 9835 (BM!)
910	*Ponerorchis monantha* (Finet) X.H. Jin, Schuit. & W.T. Jin	Kachin	Kingdon-Ward 21604 (AMES)
911	*Ponerorchis puberula* (King & Pantl.) Verm.	Putao, Kachin	Kingdon-Ward 9826 & 21064 (BM!)
912	*Ponerorchis secundiflora* (Kraenzl.) X.H.Jin, Schuit. & W.T.Jin	Recorded from Myanmar	Kingdon-Ward 9978 (AMES!)
913	*Ponerorchis tibetica* (Schltr.) X.H.Jin, Schuit. & W.T.Jin	Kachin	Forrest 26907 & 24656 (E!) & (US); Forrest 26907 (P!)
914	*Porpax conica* (Summerh.) Schuit., Y.P.Ng & H.A.Pedersen	Recorded from Myanmar	Parkinson 5288 (AMES)
915	*Porpax dickasonii* (Ormerod) Schuit., Y.P.Ng & H.A.Pedersen	Haka District, Chin State	F.G. Dickason 7359 (Holotype: AMES)
916	*Porpax elwesii* (Rchb.f.) Rolfe	Thet Kal Kwet, YePhyu, Tanintharyi	Tanaka et al. MY1731 & MY1732 (RAF, TNS)
917	*Porpax extinctoria* (Lindl.) Schuit., Y.P.Ng & H.A.Pedersen	Mon; Tanintharyi	R.C.F. Swinhoe R 114 (K!)
918	*Porpax gigantea* Deori	Listed as occurrence in Myanmar	Kurzweil and Lwin 2014
919	*Porpax lacei* (Summerh.) Schuit., Y.P.Ng & H.A.Pedersen	Dawna Range, Kyaikkami (Amherst), Mon	Lace 4751 (K!) & (E!)
920	*Porpax meirax* (N.E. Br.) King & Pantl.	Mon, Tanintharyi	Kress et al. 2003
921	*Porpax muscicola* (Lindl.) Schuit., Y.P.Ng & H.A.Pedersen	Tanintharyi	Kress et al. 2003
922	*Porpax parishii* (Lindl. & Rchb.f.) Rolfe	Mon, Tanintharyi	Kress et al. 2003
923	*Porpax pusilla* (Griff.) Schuit., Y.P.Ng & H.A.Pedersen	Listed as occurrence in Myanmar	Kurzweil and Lwin 2014
924	*Porpax summerhayesiana* (A.D.Hawkes & A.H.Heller) Schuit., Y.P.Ng & H.A.Pedersen	Recorded from Myanmar	Kress et al. 2003
925	*Porpax ustulata* (Parish & Rchb. f.) Rolfe	Putao, Kachin; Mon; Mt. Popa, Mandalay	Xiaohua Jin et al. PT-2042 (PE!), Parish 62 (K!); Tanaka et al. 036155 (MBK), Tanaka et al. 036187 (MBK, spirit collection-TNS)
926	*Pteroceras compressum* (Blume) Holtt.	Sagaing, Tanintharyi	Kress et al. 2003
927	*Pteroceras leopardinum* (Parish & Rchb. f.) Seidenf. & Smitin.	Mon, Tanintharyi	Parish 269 (K!)
928	*Pteroceras teres* (Blume) Holttum	Mon, Tanintharyi	Kress et al. 2003
929	*Renanthera coccinea* Lour.	Chin; Alaungdaw Kathapa National Park, Sagaing; YePhyu, Tanintharyi	Ye Lwin Aung PT-7320, PT-7436 (PE!)
930	***Renanthera hennisiana* Schltr.**	Reported from Myanmar	Kress et al. 2003
931	*Renanthera imschootiana* Rolfe	Mindat, Chin; Mandalay	Kingdon-Ward 22182 (BM!)
932	*Renanthera isosepala* Holttum	Listed as occurrence in Myanmar	Kurzweil and Lwin 2014
933	***Renanthera pulchella* Rolfe**	Recorded from Myanmar	Peeters s.n. (K!)
934	*Rhomboda abbreviata* (Lindl.) Ormerod	Listed as occurrence in Myanmar	Kurzweil and Lwin 2014
935	*Rhomboda moulmeinensis* (Parish & Rchb. f.) Ormerod	Moulmein, Mon; Tanintharyi	Parish 237 (K!); Lobb s.n. (K!)
936	***Rhomboda wardii* Ormerod**	Kachin	Kingdon-Ward 12889 (BM!)
937	*Rhynchostylis gigantea* Ridley	Mt. Victoria, Chin; Mt. Popa, Mandalay; Paunglaung Creek, Nay Pyi Taw-Shan Yoma; Alaungdaw Kathapa National Park, Sagaing; Kalaw, Shan	Xiaohua Jin et al. PT-5113 (PE!); Ye Lwin Aung PT-7391, PT-7425, PT-7427, PT-7571 (PE!); Lace s.n. (E!); White 245 (US); Khin Myo Htwe 72 (spirit collection-MBK, TNS), Tanaka et al. 036139 (MBK)
938	*Rhynchostylis retusa* Blume	Mt. Popa & PyinOoLwin (Maymyo), Mandalay; YePhyu, Tanintharyi	Ye Lwin Aung PT-7246 (PE!); Lace s.n. (E!); Kaulback 249 (BM!); Mokim 6 (P!) & (US); Kurz 3243 (US); Khin Myo Htwe 032884 (MBK); Tanaka 036102 (MBK, spirit collection-TNS); Tanaka et al. 036139 (MBK, spirit collection-TNS)
939	*Risleya atropurpurea* King & Pantl.	Upper Myanmar	Kingdon-Ward 1780 (E!)
940	*Robiquetia pachyphylla* (Rchb. f.) Garay	Recorded from Myanmar	s.coll. s.n. (K!)
941	*Robiquetia spathulata* (Blume) J.J. Sm.	Mon; YePhyu, Tanintharyi	Ye Lwin Aung PT-7355 (PE!)
942	*Robiquetia succisa* (Lindl.) Seidenf. & Garay	Reported from Myanmar	Kress et al. 2003
943	*Saccolabiopsis pusilla* (Lindl.) Seidenf. & Garay	Reported from Myanmar	Kress et al. 2003
944	*Sarcoglyphis flava* (Hook. f.) Garay	Mon, Tanintharyi	Kress et al. 2003
945	*Sarcoglyphis mirabilis* (Rchb. f.) Garay	Reported from Myanmar	Kress et al. 2003
946	*Sarcoglyphis smithiana* (Kerr) Seidenf.	Alaungdaw Kathapa National Park, Sagaing	Ye Lwin Aung PT-7424 (PE!)
947	*Satyrium nepalense* D. Don	Mt. Victoria, Chin; Shan; Mt. Popa, Mandalay	Xiaohua Jin et al. PT-5221 (PE!); Kingdon-Ward 1870 (E!); Farrer 1261 (E!); Maung Gale 9164 (E!)
948	*Schoenorchis fragrans* (Parish & Rchb. f.) Seidenf. & Smitin.	Mon, Tanintharyi	Kress et al. 2003
949	*Schoenorchis gemmata* (Lindl.) J.J. Sm.	Putao, Kachin	Xiaohua Jin et al. PT-2081 (PE!); Forrest 26607 (E!) & (US); Kingdon-Ward 1556 (E!)
950	*Seidenfadenia mitrata* (Rchb.f.) Garay	Tanintharyi	Kress et al. 2003
951	*Sirindhornia monophylla* (Collett & Hemsl.) H.A.Pedersen & Suksathan	Chin; Kachin; Magway; Mandalay; Sagaing; Nawngtaya, Shan	Collett s.n. (K!); Collett 766 (CAL)
952	*Smitinandia helferi* (Hook. f.) Garay	Mon, Tanintharyi	Kress et al. 2003
953	*Smitinandia micrantha* (Lindl.) Holtt.	Alaungdaw Kathapa National Park, Sagaing	Ye Lwin Aung PT-7439 (PE!)
954	*Spathoglottis affinis* de Vriese	Mt. Popa, Mandalay; Dawei (Tavoy), Tanintharyi; Rakhine	Keenan et al. 929 (E!); Murata et al. 021040 (MBK, TNS)
955	*Spathoglottis hardingiana* Parish & Rchb.f.	Listed as occurrence in Myanmar	Kurzweil and Lwin 2014
956	*Spathoglottis plicata* Blume	Mt. Popa, Mandalay	Khin Myo Htwe 4 (spirit collection-TNS), Tanaka et al. 036160 (MBK)
957	*Spathoglottis pubescens* Lindl.	Bago; Chin; Kachin; Mt. Popa & PyinOoLwin (Maymyo), Mandalay; Rakhine; Shan; Tanintharyi	Ye Lwin Aung PT-7237, PT-7238 (PE!); Farrer 1262 (E!); Lace 5515 (E!); MacGregor 819 & 820 (E!); Kingdon-Ward 1848 (E!); Kingdon-Ward 22567 (BM!); Belcher 628 & 816 (US)
958	*Spiranthes sinensis* (Pers.) Ames	Putao & Sumpra Bum, Kachin; Mindat, Chin	Xiaohua Jin et al. PT-ET 988 (PE!); Lace 3141 (E!); Kingdon-Ward 1735 & 22035 (E!); Kingdon-Ward 22848, 22656 & 22035 (BM!)
959	*Stereochilus erinaceus* (Rchb. f.) Garay	Mon, Tanintharyi	Kress et al. 2003
960	*Stereochilus hirtus* Lindl.	Tanintharyi	Kress et al. 2003
961	***Stereochilus laxus* (Rchb. f.) Garay**	Tanintharyi	Kress et al. 2003
962	*Stereosandra javanica* Blume	YePhyu, Tanintharyi	Saw Lwin et al. TNRO 5 (SING, SING [spirit])
963	*Stichorkis gibbosa* (Finet) Wood	Mon, Tanintharyi	Kress et al. 2003
964	*Strongyleriapannea (Lindl.)* Schuit., Y.P.Ng & H.A.Pedersen	PyinOoLwin (Maymyo), Mandalay; Mt. Victoria, Chin	Forrest 25121 & 27028 (E!) & (NY!) & (BM!) & (US); Kingdon-Ward 1552 (E!); Cooper 6087 (E!); s.n. (K!)
965	*Taeniophyllum glandulosum* Blume	InnDawGyi Wildlife Sanctuary, Kachin State	Kurzweil & Lwin 2790 (SING spirit)
966	***Tainia hennisiana* (Schltr.) P.F.Hunt**	Reported from Myanmar	Kress et al. 2003
967	*Tainia latifolia* (Lindl.) Rchb. f.	Chin; Putao, Kachin; Mandalay; Sagaing; Upper Chindwin	Xiaohua Jin et al. PT-2106 (PE!), Ye Lwin Aung PT-7142 (PE!); Glin 5761 (K!); Parish 253 (K!); Keenan et al. 3429 (E!)
968	*Tainia minor* Hook.f.	Laktang, Kachin	Kingdon-Ward 3229 (E!); Kingdon-Ward 20938 (BM!)
969	*Tainia wrayana* (Hook.f.) J.J.Sm.	Reported from Myanmar	Kress et al. 2003
970	*Thecostele alata* (Roxb.) Parish & Rchb. f.	Mon, Tanintharyi	Kress et al. 2003
971	*Thelasis carinata* (Blume) Rchb. f.	Reported from Myanmar	Kress et al. 2003
972	*Thelasis khasiana* Hook.f.	Putao, Kachin	Xiaohua Jin et al. PT-5274 (PE!), Ye Lwin Aung PT-7065, PT-7077, PT-7095, PT-7110 (PE!)
973	*Thelasis micrantha* (Brongn.) J.J. Sm.	Listed as occurrence in Myanmar	Kurzweil and Lwin 2014
974	*Thelasis perpusilla* (C.S.P.Parish & Rchb.f.) Schuit.	Hpa-an, Kayin; Mon; Tanintharyi; Pyu Kyoon, Bago; Mandalay	Xiaohua Jin et al. PT-2552 (PE!); R.C. Swinhoe 22 (K!); Brandis s.n. (K!)
975	*Thelasis pygmaea* (Griff.) Lindl.	Chin, Mon, Tanintharyi	Kress et al. 2003
976	*Thrixspermum calceolus* (Lindl.) Rchb. f.	Reported from Myanmar	Kress et al. 2003
977	*Thrixspermum centipeda* Lour.	Putao, Kachin; Dawei (Tavoy), Tanintharyi	Xiaohua Jin et al. PT-ET 1322 (PE!); Keenan et al. 5450 (E!)
978	*Thrixspermum merguense* (Hook. f.) Kuntze	Mon; YePhyu, Tanintharyi	Ye Lwin Aung PT-7348 (PE!)
979	*Thrixspermum scortechinii* (Hook. f.) Ridl.	Mon	Kress et al. 2003
980	*Thrixspermum trichoglottis* (Hook. f.) Kuntze	YePhyu, Tanintharyi	Ye Lwin Aung PT-7329 (PE!)
981	*Thunia alba* Rchb. f.	Mt. Victoria & Mindat, Chin; Putao, Kachin; Mt. Popa, Mandalay; Rakhine; Kalaw, Shan; YePhyu, Tanintharyi; Mon	Xiaohua Jin et al. PT-ET 109 (PE!), Xiaohua Jin et al. PT-5116 (PE!); Ye Lwin Aung PT-7010, PT-7288 (PE!); Cooper 6068 (E!); Kingdon-Ward 22320 (BM!); Khin Myo Htwe 40 (spirit collection-TNS)
982	*Thunia bensoniae* Hook. f.	Chin, Mon	Kress et al. 2003
983	***Thunia brymeriana* Rolfe**	Recorded from Myanmar	s.coll. s.n. (K!)
984	***Thunia candidissima* Rchb. f.**	Reported from Myanmar	Kress et al. 2003
985	*Thunia pulchra* Rchb.f.	Putao, Kachin; Mon	Qiang Liu 455 (HITBC!); Parish 5 & 199 (K!)
986	*Thuniopsis cleistogama* L.Li, D.P.Ye & Shi J.Li	Mt. Popa, Mandalay; Kanpetlet, Chin	Cho et al. MM-0507 (HHU); Kang et al. 2019 [photographic record from Chin State]
987	*Tipularia josephi* Rchb.f. ex Lindl.	Kachin	Kingdon-Ward 12991 & 13242 (BM!)
988	*Trachoma coarctatum* (King & Pantl.) Garay	Reported from Myanmar	Kress et al. 2003
989	*Trichoglottis bipunctata* (Parish & Rchb. f.) Tang & F.T. Wang	Mon, Tanintharyi	Kress et al. 2003
990	*Trichoglottis dawsoniana* Rchb.f.	Mon, Tanintharyi	Kress et al. 2003
991	*Trichoglottis fasciata* Rchb.f.	Tanintharyi	Kress et al. 2003
992	*Trichoglottis ramosa* (Lindl.) Senghas	Mt. Popa, Mandalay	Khin Myo Htwe 15 (spirit collection-TNS)
993	*Trichoglottis ventricularis* Kocyan & Schuit.	Reported from Myanmar	Kress et al. 2003
994	*Trichotosia aurea* (Ridl.) Carr	Putao, Kachin	Ye Lwin Aung PT-7146 (PE!)
995	*Trichotosia crassicaulis* (Hook.f.) Kraenzl.	Putao, Kachin	Xiaohua Jin et al. PT-2400 (PE!)
996	*Trichotosia dasyphylla* (Parish & Rchb. f.) Krzl.	Mt. Victoria, Chin; Bago; Tanintharyi	Ye Lwin Aung PT-7569 (PE!); Kurz 1432 (K!)
997	*Trichotosia pauciflora* Blume	Tanintharyi	Kress et al. 2003
998	*Trichotosia pulvinata* (Lindl.) Krzl.	Putao, Kachin; Bago; Tanintharyi	Xiaohua Jin et al. PT-5345 (PE!)
999	*Trichotosia rotundifolia* (Ridl.) Krzl.	Mon, Tanintharyi	Kress et al. 2003
1000	*Trichotosia velutina* (Lodd. ex Lindl.) Krzl.	Tanintharyi	Kress et al. 2003
1001	*Trichotosia vestita* (Wall. ex Lindl.) Kraenzl.	Tanintharyi	Kress et al. 2003
1002	*Tropidia angulosa* Blume	Putao, Kachin; Sagaing; Tanintharyi	Xiaohua Jin et al. PT-2094 (PE!); McMillen 130 (US); Belcher B-C-956 (US)
1003	*Tropidia curculigoides* Lindl.	Putao, Kachin; Mt. Popa, Mandalay	Xiaohua Jin et al. PT-ET 1378, PT-ET 458 (PE!), Than Than Aye & Khin Myo Htwe 020642 (MBK), Murata et al. 020892 (MBK), Than Than Aye & Khin Myo Htwe 021361 (MBK, TNS)
1004	*Tuberolabium elobe (Seidenf.)* Seidenf.	Putao, Kachin	Xiaohua Jin et al. PT-ET 245 (PE!)
1005	*Uncifera obtusifolia* Lindl.	Putao, Kachin; YePhyu, Tanintharyi	Ye Lwin Aung PT-7304, PT-7027, PT-7032, PT-7168 (PE!)
1006	***Uncifera verrucosa* Summerh.**	Recorded from Myanmar	Kingdon-Ward 21171 (BM!)
1007	*Vanda ampullacea* (Roxb.) L.M.Gardiner	Falam, Chin; Mandalay; Mon	Daun 20 (K!); Swinhoe 44 (K!); Kurz 328 (BM!)
1008	*Vanda bensonii* Bateman	Mt. Victoria & Haka, Chin; Bago; Mt. Popa & PyinOoLwin (Maymyo), Mandalay; Mon; Alaungdaw Kathapa National Park, Sagaing; Tanintharyi	Ye Lwin Aung PT-7395, PT-7415, PT-7428, PT-7570 (PE!); Benson s.n. (K!); Venning 51 (K!); Lace s.n. (E!); Kingdon-Ward 21803 (BM!); Khin Myo Htwe 12 (spirit collection-TNS); Tanaka et al. 036152 (MBK)
1009	*Vanda brunnea* Rchb. f.	Falam, Chin; Putao, Kachin; Mt. Popa, Mandalay; Shan	Xiaohua Jin et al. PT-5346 (PE!), Daun 3 (K!); Khin Myo Htwe 97 (spirit collection-MBK), Khin Myo Htwe 16 (spirit collection-TNS)
1010	*Vanda coerulea* Griff. ex Lindl.	Chin; Kachin; PyinOoLwin (Maymyo), Mandalay; Shan; Yangon	Bogg B (K!); Lace s.n. (E!)
1011	*Vanda coerulescens* Griff.	Pyay (Prome), Bago; Falam, Chin; Kachin; Mt. Popa & PyinOoLwin (Maymyo), Mandalay; Alaungdaw Kathapa National Park, Sagaing; Shan	Ye Lwin Aung PT-7397, PT-7412, PT-7419, PT-7437 (PE!); Benson s.n. (K!); Collett 544 (K!); Daun 24 (K!); Samuel 13523 & 13559 (K!); Forrest 7450 (E!); Khin Myo Htwe 81 (spirit collection-MBK) & 89 (spirit collection-TNS)
1012	*Vanda cristata* Lindl.	Putao, Kachin; Paunglaung Creek, Nay Pyi Taw-Shan Yoma; YePhyu, Tanintharyi	Ye Lwin Aung PT-7322, PT-7372, PT-7373 (PE!); Xiaohua Jin et al. PT-6869 (PE!)
1013	*Vanda curvifolia* (Lindl.) L.M.Gardiner	Bago; Mandalay; Yangon; Dawei (Tavoy), Tanintharyi	Griffith s.n. (K!); Mokim 454 (BM!) & (P!)
1014	*Vanda denisoniana* Benson & Rchb. f.	Reported from Myanmar	Kress et al. 2003
1015	*Vanda flabellata* (Rolfe ex Downie) Christenson	Taunggyi, Shan State	Ye Lwin Aung PT-7598 (PE!)
1016	*Vanda garayi* (Christenson) L.M.Gardiner	Alaungdaw Kathapa National Park, Sagaing	Ye Lwin Aung PT-7410 (PE!)
1017	*Vanda liouvillei* Finet	Mt. Popa, Mandalay	s.coll. s.n. (K!); Khin Myo Htwe 035062 (MBK), Khin Myo Htwe 6 (spirit collection-TNS), Khin Myo Htwe 20 (spirit collection-TNS), Khin Htwe 106 (spirit collection-MBK, TNS)
1018	***Vanda longitepala* D.L.Roberts, L.M.Gardiner & Mote**	Myitkyina District, Kachin State	Kermode 17331 (Holotype: K!)
1019	*Vanda petersiana* Schltr.	Reported from Myanmar	Kress et al. 2003
1020	*Vanda rubra* (Lindl.) L.M.Gardiner	Recorded from Myanmar	Griffith s.n. (P!)
1021	*Vanda tessellata* Hook. ex G. Don	Chin, Tanintharyi	Kress et al. 2003
1022	*Vanda testacea* (Lindl.) Rchb. f.	Mt. Popa, Mandalay; Bago; Mon; Rakhine; Tanintharyi	Khin Myo Htwe 79 (spirit collection-MBK), Khin Myo Htwe 78 & 83 (spirit collection-MBK, TNS), Khin Myo Htwe 82 (spirit collection-MBK, TNS)
1023	***Vanda vipanii* Rchb. f.**	Falam, Chin; Taunggyi, Shan	Kingdon-Ward 22961 (BM!); Abdul Khalil s.n. (BM!); s.coll. s.n. (K!)
1024	*Vandopsis gigantea* (Lindl.) Pfitzer	Reported from Myanmar	Kress et al. 2003
1025	***Vandopsis shanica* (Phillimore & W.W. Sm.) Garay**	Reported from Myanmar	Kress et al. 2003
1026	*Vandopsis undulata* (Lindl.) J.J.Sm.	Recorded from Myanmar	Forrest 13729 (E!)
1027	*Vanilla aphylla* Blume	Recorded from Myanmar	Parish 286 (K!)
1028	*Vanilla planifolia* Andrews	Listed as occurrence in Myanmar	Kurzweil and Lwin 2014
1029	*Vanilla siamensis* Rolfe ex Downie	Listed as occurrence in Myanmar	Kurzweil and Lwin 2014
1030	*Vrydagzynea albida* (Blume) Blume	Reported from Myanmar	Kress et al. 2003
1031	*Vrydagzynea nuda* Blume	Putao, Kachin	Xiaohua Jin et al. PT-6469 (PE!)
1032	*Zeuxine affinis* (Lindl.) Trimen	Mt. Popa, Mandalay	Kingdon-Ward 21758 (BM!); s.coll. s.n. (E!); Akiyama et al. 030199 (MBK); Ohi-Toma 035001 (MBK)
1033	*Zeuxine flava* (Lindl.) Benth.	Myitkyina, Kachin; Kanpetlet, Chin; Kalaw, Shan; Mt. Popa, Mandalay; Tanintharyi	Dickason 8448 (K!); Kermode 16609 (K!)
1034	*Zeuxine goodyeroides* Lindl.	Bago; Dawna range, Kayin	Lace 4606 (E!)
1035	*Zeuxine gracilis* (Breda) Blume	Mon, Tanintharyi	Kress et al. 2003
1036	*Zeuxine longilabris* (Lindl.) Trimen	Galon Reserve, Katha, Sagaing	Lace s.n. (E!)
1037	*Zeuxine membranacea* Lindl.	Mogok, Mandalay	Lace 5070 (E!)
1038	*Zeuxine nervosa* (Wall. ex Lindl.) Trimen	Hpa-an or Myawaddy District, Kayin State	Burkill 24458 p.p. (K)
1039	*Zeuxine parvifolia* (Ridl.) Seidenf.	Listed as occurrence in Myanmar	Kurzweil and Lwin 2014
1040	*Zeuxine strateumatica* (L.) Schltr.	PyinOoLwin (Maymyo), Mandalay; Shan	Swinhoe s.n. (K!); White 206 & 207 (US)

1. Accepted taxon name and its author (s). Endemic species are mentioned in bold text, 76 species in total.

2. Local Distribution in Myanmar, usually Region/State (Provincial) level. In Myanmar, there are 15 administrative provinces including Nay Pyi Taw Union Territory, seven Regions (Ayeyarwaddy, Bago, Magway, Mandalay, Sagaing, Tanintharyi and Yangon) and seven States (Chin, Kachin, Kayah, Kayin, Mon, Rakhine and Shan). Specific localities are provided if known.

3. Specimen Citations: Collector (s), Collection number (s) and Herbarium codes (Thiers 2019, http://sweetgum.nybg.org/ih/). All specimens cited with exclamation mark (!) have been examined. Some specimens without exclamation mark (!) are studied and known from literature of new species description or new species records.
